# L1CAM Promotes Human Endometrial Cancer Via NF-κB Activation

**DOI:** 10.3390/cancers18020198

**Published:** 2026-01-08

**Authors:** Hiroyuki Kurosu, Hiroshi Asano, Alaa-eldin Salah-eldin, Kazuya Hamada, Shugo Tanaka, Asuka Ishii, Issei Kawakita, Kentaro Kumagai, Kensuke Nakazono, Yuko Katayama, Rino Saito, Chihiro Terasaka, Sari Iwasaki, Satoshi Tanaka, Atsushi Niida, Hidemichi Watari, Koji Taniguchi

**Affiliations:** 1 Department of Pathology, Faculty of Medicine and Graduate School of Medicine, Hokkaido University, Sapporo 060-8638, Japan; 2 Department of Obstetrics and Gynecology, Faculty of Medicine and Graduate School of Medicine, Hokkaido University, Sapporo 060-8638, Japan; 3 Department of Zoology, Faculty of Science, Aswan University, Sahary, Aswan P.O. Box 81528, Egypt; 4 Laboratory of Molecular Medicine, Human Genome Center, The Institute of Medical Science, The University of Tokyo, Tokyo 106-8639, Japan

**Keywords:** endometrial cancer, L1CAM, NF-κB, cell cycle, drug resistance

## Abstract

**Background/Objectives**: Endometrial cancer is one of the most common gynecological malignancies, with increasing incidence and mortality rates, particularly in developed countries. L1 cell adhesion molecule (L1CAM) has been identified as a poor prognostic factor for human endometrial cancer; however, the molecular mechanisms underlying its role in tumor progression remain unclear. **Methods**: We investigated the biological significance of L1CAM in human endometrial cancer using multiple cell lines. Functional analyses, including cell proliferation, cell cycle, and apoptosis assays, were performed after *L1CAM* knockdown or overexpression. **Results**: L1CAM promoted the transition of endometrial cancer cells from the G0/G1 phase and enhanced cell proliferation. *L1CAM* knockdown inhibited NF-κB signaling by reducing NF-κB (p65) phosphorylation and downregulating the expression of downstream targets such as *TNF*. Overexpression of constitutively active IKKβ restored the proliferation defect caused by *L1CAM* knockdown, supporting the role of NF-κB as a key downstream effector of L1CAM. Immunohistochemical analysis revealed a significant correlation between L1CAM expression and nuclear NF-κB (p65) positivity rates in human patient samples. Furthermore, combination therapy with cisplatin and an IKK inhibitor enhanced the anti-proliferative effect. **Conclusions**: Our study demonstrated that L1CAM promotes proliferation and chemotherapy resistance in human endometrial cancer through activation of the NF-κB signaling pathway. Therapeutic strategies targeting the L1CAM-NF-κB pathway may represent a promising treatment option for improving prognosis in L1CAM-positive human endometrial cancer.

## Simple Summary

The incidence and mortality rates of endometrial cancer are increasing, particularly in developed countries, and the prognosis for advanced cases has not improved. The L1 cell adhesion molecule (L1CAM) has been identified as a poor prognostic factor in human endometrial cancer, but the molecular mechanisms underlying its role in tumor progression remain unclear. We demonstrated that *L1CAM* knockdown and overexpression in human endometrial cancer cell lines promote progression from the G0/G1 phase and enhance cell proliferation. Furthermore, we showed that the L1CAM-NF-κB pathway is involved in these effects. Immunohistochemical staining confirmed a significant correlation between L1CAM expression and nuclear NF-κB positivity in human patient specimens. Additionally, combination therapy with cisplatin and an IKK inhibitor enhanced the antiproliferative effect, suggesting the involvement of this pathway in chemotherapy resistance. Our findings suggest that therapeutic strategies targeting the L1CAM-NF-κB pathway represent a promising treatment option for improving prognosis in L1CAM-positive human endometrial cancer.

## 1. Introduction

Endometrial cancer is one of the most common gynecological malignancies worldwide. According to recent epidemiological data, the incidence of endometrial cancer is increasing due to lifestyle factors such as aging, obesity, and metabolic syndrome, especially in developed countries [1]. By 2022, it is predicted that approximately 420,242 new cases will be diagnosed worldwide, making it the sixth most common cancer in women. The number of cases and deaths is also increasing, and the increase is particularly noticeable in developed countries [2].

Endometrial cancer has been broadly classified into type I, which is typified by endometrial cancer, and type II, which is typified by serous carcinoma and clear cell carcinoma. In recent years, whole exome sequencing analysis by The Cancer Genome Atlas (TCGA) has led to the classification of endometrial cancer into four new clusters (POLE ultramutated, microsatellite instability (MSI) hypermutated, copy-number low, and copy-number high) [3]. In 2023, the International Federation of Gynecology and Obstetrics (FIGO) staging system for endometrial cancer was revised based on the TCGA classification [4]. Subsequently, the ProMisE and Trans-PORTEC classifications were proposed as clinical applications of the TCGA classification, and we have entered an era in which molecular evaluation is considered important for improving the prognosis of endometrial cancer [5,6,7,8,9,10,11].

L1 cell adhesion molecule (L1CAM) is a transmembrane glycoprotein that belongs to the immunoglobulin superfamily and is involved in various cellular functions, including cell adhesion, migration, and signal transduction [12]. Structurally, L1CAM is composed of an extracellular domain containing six immunoglobulin-like structures and five fibronectin type III-like repeats, a transmembrane domain, and a cytoplasmic domain that mediates intracellular signal transduction. This molecule plays a crucial role in neural development and has also been implicated in tumor progression and metastasis in various cancers [12,13,14,15,16].

L1CAM has not been detected in either the proliferative or secretory phases of normal endometrial epithelium [17]. L1CAM expression has been observed in some endometrial cancers, and its expression has been reported as a poor prognostic factor [15,16]. Recently, there have been increasing reports that adding L1CAM to the ProMisE classification will enable more accurate prognosis prediction, and it is being incorporated into clinical trials [18,19,20,21].

Clinical data have shown that L1CAM is a poor prognostic factor in many cases of endometrial cancer, but the mechanisms involved in poor prognosis remain unclear. In particular, the relationship between L1CAM expression and therapeutic resistance has not been fully elucidated. Therefore, this study aims to focus on and elucidate the mechanisms involved in poor prognosis of L1CAM in human endometrial cancer.

## 2. Materials and Methods

### 2.1. Cell Lines and Culture

The human uterine endometrial cancer cell lines, HHUA and HOUA was obtained from Institute of Physical and Chemical Research (Saitama, Japan), SPAC-1-L and SPAC-1-S were provided by the laboratory of Dr. Y. Hirai from the Department of Gynecology, Cancer Institute Hospital (Tokyo, Japan), HEC-1 was provided by National Institute of Health Sciences (Kanagawa, Japan), Ishikawa was provided by the laboratory of Dr. M. Nishida from the Department of Gynecology, Tsukuba University (Ibaraki, Japan). HEK 293T was obtained from ATCC (Manassas, VA, USA). SPAC-1-L, SPAC-1-S, HEC-1, Ishikawa, and HEK293T cell lines were cultured in DMEM (Nacalai Tesque, Kyoto, Japan) supplemented with 10% FBS, and 1% penicillin/streptomycin. HHUA and HOUA cell lines were cultured in Ham’s F-12 with L-Gln (FUJIFILM Wako Pure Chemical Corp, Osaka, Japan) supplemented with 15% FBS, and 1% penicillin/streptomycin. Mycoplasma testing was performed using MycoAlert Mycoplasma Detection Kit (Lonza, Basel, Switzerland) or BioMycoX Mycoplasma PCR Detection Kit (CellSafe, Yongin-si, Korea), and all the cell lines used in this study were found to be Mycoplasma negative. Cells were used within ten cell passages, and the assays were conducted using cells within two months of thawing. Cell observation and imaging were performed using the BZ-X800 (Keyence, Osaka, Japan).

### 2.2. Lentiviral Production

The lentiviral packaging plasmids psPAX2 (Addgene, Watertown, MA, USA; #12260) and pMD2.G (Addgene; #12259), which were gifts from Dr. Didier Trono (Ecole Polytechnique Federale de Lausanne (EPFL), Lausanne, Switzerland), were cotransfected for lentiviral production with plasmids, which were human L1CAM-specific pLKO_005 shRNA plasmids, FLAG-tagged human L1CAM-expressing plasmid, and FLAG-tagged I kappa B kinase (IKK)β(EE)-expressing plasmid. shL1CAM was obtained from MISSION shRNA Library (Merck, Darmstadt, Germany). Each sequence is shown in Supplementary Table S1.

Control shRNA (shCtrl) was used as a nontargeting shRNA. Transfection into HEK293T cells was performed using polyethylenimine “MAX” (PEI MAX, Polysciences, Warrington, PA, USA). Within 24 h of transfection, the medium was changed to remove the transfection reagents. The virus-containing medium was harvested 48 and 72 h after the initial transfection.

For transduction into cells, the virus-containing medium was spun down at 800× *g* for 10 min at room temperature, and the supernatant was carefully collected. For knockdown experiments, the collected supernatant was used directly. For overexpression experiments, the viral solution was concentrated before use. The viral concentrate was prepared by dissolving 80 g of PEG-8000 and 14.0 g of NaCl in 80 mL of MillQ water and 20 mL of 10 × PBS (pH 7.4). The final pH was adjusted to 7.0–7.2, and the solution was sterilized by autoclaving at 121 °C for 15 min. For virus concentration, one volume of the concentrator was added to three volumes of the virus supernatant. After mixing thoroughly, the mixture was incubated at 4 °C for at least 4 h. Subsequently, it was centrifuged at 1600× *g* for 60 min at 4 °C, and the supernatant was removed. The virus pellet was resuspended in 1/10 of its original volume of medium (serum-free, antibiotic-free) to obtain a concentrated virus solution. When infecting cell lines, 8 µg/mL polybrene (Nacalai Tesque) was added. Before use, the virus-infected cells were selected using puromycin (InvivoGen, San Diego, CA, USA).

### 2.3. mRNA Preparation and Real-Time PCR

Total RNA was extracted using RNAiso Plus (Takara Bio, Kusatsu, Japan), and reverse transcription was performed using GoTaq 2-Step RT-qPCR System (Promega, Madison, WI, USA). Real-time PCR analysis was performed using the CFX Opus 96 Real-time PCR System and the CFX Connect Real-Time PCR System (Bio-Rad, Hercules, CA, USA). All primer sets yielded a single product of the expected size. Relative expression levels were normalized to *GAPDH*. The sequences of the primers used in this study are shown in Supplementary Table S2. Relative mRNA expression level was calculated as Fold Change = 2^−[(CTtarget, sample − CTref, sample) − (CTtarget, control − CTref, control)]^. For comparisons involving endometrial carcinoma cell lines, HEC-1 values were set as 1; for other comparisons, shCtrl or empty values were set as 1.

### 2.4. Western Blotting

Protein was collected using a lysis buffer consisting of RIPA buffer supplemented with protein inhibitor and sodium orthovanadate.

The collected proteins were measured for absorbance at 562 nm using Infinite 200 PRO M Plex (TECAN), following the protocol of the Takara BCA Protein Assay Kit (Takara Bio). The measured values were compared to a standard curve to calculate the sample concentration. After dilution with the lysis buffer, the final concentration was adjusted to 2.0 µg/µL. Equal amounts of protein from each sample were separated using SDS-PAGE and blotted onto polyvinylidene difluoride membranes. Protein blots were hybridized with the indicated primary antibody of interest and followed by HRP-conjugated secondary antibody, followed by detection with Chemi-Lumi One Super (Nacalai Tesque) or Chemi-Lumi One Ultra (Nacalai Tesque) and Amersham Imager 680 (GE Healthcare Life Science, Chicago, IL, USA). Primary antibodies included antibodies against L1CAM (BioLegend, San Diego, CA, USA; clone 14.10, 1:1000), Phospho-NF-κB p65(Ser536) (Cell Signaling Technology, Danvers, MA, USA; #3033, 1:1000), NF-κB p65(D14E12)XP^®^ (Cell Signaling Technology, #8242, 1:1000), Phospho-P44/42 MAPK (ERK1/2) (Thr202/Tyr204) (Cell Signaling Technology, #9101, 1:1000), ERK2 (Santa Cruz Biotechnology, Dallas, TX, USA; sc-1647, 1:1000), OctA-Probe(H-5) (Santa Cruz Biotechnology, sc-166355, 1:1000) and GAPDH (FUJIFILM Wako Pure Chemical Corp, #015-25473, 1:10,000).

### 2.5. Cell Proliferation Assay

Cell proliferation ability was measured by seeding cells into 96-well plates at an initial density of 2000 cells per well. Every 24 h after seeding for 4 days, 10 µL of the Cell Counting Kit-8 (Dojindo, Kumamoto, Japan) was added to the cell culture medium for 2 h, and the absorbance for each well was measured at a 450-nm wavelength using Infinite 200 PRO M Plex (TECAN, Männedorf, Switzerland).

### 2.6. Cell Cycle Analysis Assay

Cells were harvested by trypsinization, washed twice with PBS, and mixed with Cell Cycle Assay Solution Blue (C549, Dojindo) according to the manufacturer’s instructions. After incubation, the stained cells were analyzed using a Spectral Analyzer SA3800 (SONY, Tokyo, Japan). SPAC-1-L was collected and measured 48 h after shRNA transduction. Selected overexpressing cell lines (HHUA and Ishikawa) were collected 48 h after reseeding. The analysis was performed using FlowJo^TM^ software (version 10.10) (BD Biosciences, Ashland, OR, USA) with the Dean-Jett-Fox model.

### 2.7. Apoptosis Assay

Apoptosis was analyzed using an Annexin V-FITC Apoptosis Detection Kit (Nacalai Tesque, Japan). Cells were double-stained with FITC-conjugated Annexin V and propidium iodide (PI). Annexin V binds to apoptotic cells with exposed phosphatidylserine, whereas PI binds to the late apoptotic and necrotic cells with damaged membranes. Staining was performed according to the manufacturer’s instructions. Stained cells were analyzed using a Spectral Analyzer SA3800 (SONY). The analysis was performed using FlowJo^TM^ software (version 10.10) (BD Biosciences).

### 2.8. RNA-Seq Analysis

Total RNA was extracted using RNAiso Plus (Takara Bio) according to the manufacturer’s protocol. The quality and integrity of the extracted RNA were evaluated using an Agilent 2100 Bioanalyzer (Agilent Technologies, Santa Clara, CA, USA). Only samples with an RNA Integrity Number (RIN) ≥ 9 were used for library preparation.

RNA-seq library preparation and sequencing were outsourced to Rhelixa Inc. (Tokyo, Japan). Poly(A)-based mRNA enrichment was performed using the NEBNext Poly(A) mRNA Magnetic Isolation Module (Cat No. E7490, New England Biolabs, Ipswich, MA, USA). Strand-specific libraries were prepared using the NEBNext Ultra II Directional RNA Library Prep Kit (Cat No. E7760, New England Biolabs).

Sequencing was conducted on an Illumina NovaSeq 6000 (Illumina, San Diego, CA, USA) platform with paired-end 150 bp reads (PE150). Each sample generated approximately 4 Gbp of sequencing data, corresponding to an average of 26.7 million reads per sample (13.3 million read pairs). Sequencing reads were mapped to the human reference genome (hg38) using proprietary pipelines provided by Rhelixa Inc. Downstream analyses, including quality control, differential gene expression analysis, and data normalization, were also conducted by Rhelixa Inc. Genes with log2FC < −1 were analyzed for functional enrichment using DAVID (Database for Annotation, Visualization, and Integrated Discovery, v6.8). KEGG (Kyoto Encyclopedia of Genes and Genomes) pathway enrichment results were referenced to identify significantly enriched pathways associated with the dataset.

### 2.9. Luciferase Reporter Assay

Luciferase reporter gene luc2P (pGL4.32 [luc2P/NF-κB-RE/Hygro]) was obtained from Promega (Madison, WI, USA). The promoter region of human *tumor necrosis factor* *(TNF*) (−2000 to 0 bp) was amplified by genomic DNA PCR and cloned into the luciferase reporter plasmid pGL4.10 [luc2] vector (Promega). 1 µg of reporter gene plasmid, 1 μg human L1CAM-expressing plasmid, and 1 μg of β-galactosidase plasmid were cotransfected into HHUA, SPAC-1-L, and Ishikawa cells using PEI MAX (Polysciences). After 72 h of incubation, the cells were lysed with reporter lysis buffer (Promega). Luciferase activity was measured using the Infinite 200 PRO M Plex (TECAN) with the Luciferase Assay System (Promega). β-galactosidase activity was detected using 2-nitrophenyl β-D-galactopyranoside (FUJIFILM Wako Pure Chemical Corp) as a substrate and Infinite 200 PRO M Plex. Luciferase activity was normalized to β-galactosidase enzyme activity, which is an internal control for transfection efficiency. Each experiment was repeated at least three times. The plasmid sequences used in this study are shown in Supplementary Table S3.

### 2.10. Immunohistochemistry

The tissue microarray (TMA) specimens used in immunohistochemistry are as described in the preceding paper [22]. The preceding paper also describes the specimen processing before immunohistochemical staining [22]. This TMA specimen was prepared from 190 cases of endometrial carcinoma, including carcinosarcomas, that underwent surgical therapy, including lymph node dissection, at our institution between 2003 and 2015 and were pathologically staged. Two spots per case were evaluated. Twenty spots with insufficient tissue were excluded from evaluation. Sections were stained with anti-L1CAM antibody (clone 14.10, BioLegend) diluted 1:50. Another section was stained with NF-κB p65 (D14E12) XP^®^(Cell Signaling Technology, #8242) diluted 1:1200. The specimens were evaluated using digital pathology images with NDP.view2 software (version 2.9.29; Hamamatsu Photonics K.K., Hamamatsu, Japan). For L1CAM, staining 10% or more was judged to be positive. For NF-κB (p65), those that showed nuclear staining were considered positive. The results were evaluated using the chi-square test. This study was approved by the institutional review board of Hokkaido University Hospital (protocol code 017-0269; 17 January 2018) and was conducted according to the principles of the Declaration of Helsinki.

### 2.11. Drug Administration Assay

IKK inhibitor (Selleck, S2882, Tokyo, Japan) and cisplatin (CDDP) (Selleck, S1166, Tokyo, Japan) were used to investigate their effects on cell viability. The drugs were dissolved in appropriate solvents as recommended by the manufacturer and diluted to the desired concentrations in a cell culture medium. Cells were seeded into 6-well or 96-well plates and allowed to adhere overnight before drug treatment. After 48 h of drug treatment, cells were harvested for protein extraction. Western blotting was performed as described above. Cell viability was evaluated using the same method as the cell proliferation assay described above.

### 2.12. Statistical Analysis

All statistical analyses, data processing, and graph generation were performed using R-4.4.0. All experiments were conducted in triplicate, and data are presented as means ± SEM (*n* = 3). Student *t*-test was used to compare the two groups. For comparisons among the three groups, Dunnett’s test was performed. Statistical significance was determined as *p* < 0.05.

## 3. Results

### 3.1. L1CAM Expression Levels Vary Among Human Endometrial Cancer Cell Lines

**Figure 1:**
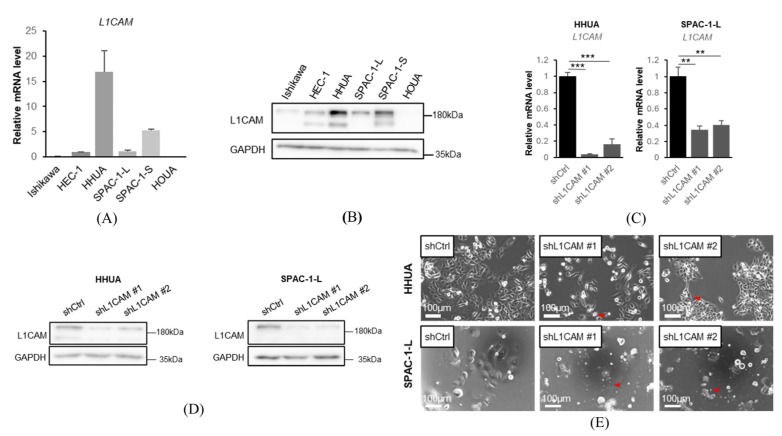
L1CAM is expressed at various levels in human endometrial cancer cells. (**A**) mRNA levels of *L1CAM* in Ishikawa, HEC-1, HHUA, SPAC-1-L, SPAC-1-S, HOUA. *GAPDH* was used as an internal control. (**B**) Western blotting of L1CAM and GAPDH in Ishikawa, HEC-1, HHUA, SPAC-1-L, SPAC-1-S, and HOUA. GAPDH was used as a loading control. (**C**) mRNA levels of *L1CAM* in HHUA and SPAC-1-L with control or *L1CAM* knockdown. *GAPDH* was used as an internal control. (**D**) Western blotting of L1CAM and GAPDH in HHUA and SPAC-1-L with control or L1CAM knockdown. GAPDH was used as a loading control. (**E**) Pictures of HHUA and SPAC-1-L with control or *L1CAM* knockdown. Red arrowheadshows morphological changes (*p* < 0.01 **, *p* < 0.001 ***). The uncropped blots are shown in Supplementary Figure S1.

We first evaluated the expression of L1CAM in human endometrial cancer cell lines. A correlation was observed between mRNA expression and protein expression. L1CAM expression was detected in SPAC-1-L and HEC-1, with high expression in HHUA and SAPC-1-S and low expression in Ishikawa and HOUA (Figure 1A,B). For knockdown experiments, we used HHUA cells, which exhibited high L1CAM expression, and SPAC-1-L cells, which showed moderate expression. For overexpression experiments, we used HHUA cells, which already expressed L1CAM, and Ishikawa cells, which exhibited low expression. HEC-1 cells were excluded from functional assays due to the presence of *PMS2* and *MSH6* mutations, which may classify them as MSI-positive according to the TCGA classification algorithm and potentially place them in a favorable prognosis group unaffected by L1CAM expression. A summary table of mutations in the cell lines is provided in Supplementary Table S4.

To evaluate the function of L1CAM on human endometrial cancer cells, we generated *L1CAM* knockdown cell lines using shRNAs against *L1CAM* in HHUA and SPAC-1-L. SPAC-1-L and SPAC-1-S were cell lines of the same origin, but SPAC-1-S grew slowly and was not used in this study [23]. *L1CAM* knockdown was confirmed by real-time PCR and Western blotting (Figure 1C,D). In the L1CAM knockdown cells, a reduction in cell proliferation accompanied by morphological changes, such as spindle-shaped cells, was observed (Figure 1E).

### 3.2. L1CAM Enhances Cell Proliferation and Cell Cycle, but Does Not Induce Apoptosis in Human Endometrial Cancer Cell Lines

**Figure 2:**
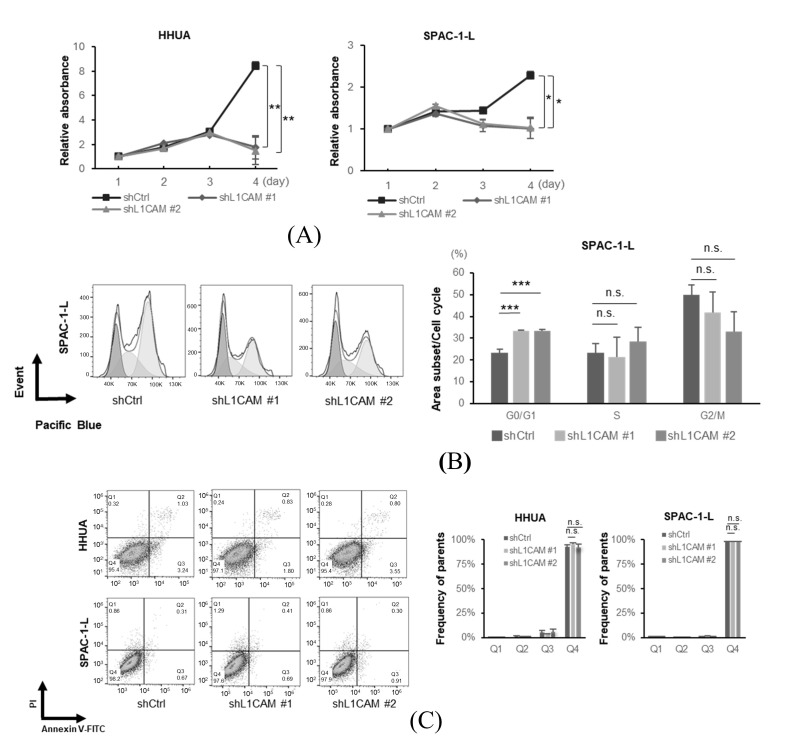
L1CAM promotes cell proliferation by regulating the cell cycle, but does not affect apoptosis in human endometrial cancer cells. (**A**) MTT assay of HHUA and SPAC-1-L with control or *L1CAM* knockdown. (**B**) Cell cycle assay of SPAC-1-L with control or *L1CAM* knockdown. (**C**) Apoptosis assay of HHUA and SPAC-1-L with control or *L1CAM* knockdown (*p* < 0.05 *, *p* < 0.01 **, *p* < 0.001 ***, n.s.: not significant).

In a cell proliferation assay, a significant decrease was observed in the *L1CAM* knockdown group on day 4 in HHUA and SPAC-1-L (Figure 2A). These results suggest that L1CAM promotes cell proliferation in human endometrial cancer cells.

Next, we performed flow cytometry experiments to elucidate the mechanisms underlying the differences in proliferation. Cell cycle analysis revealed that *L1CAM* knockdown in SPAC-1-L cells induced accumulation in the G0/G1 phase (Figure 2B). The percentage of cells in the G0/G1 phase in the control group was about 23.2%, whereas in the *L1CAM* knockdown group, it was increased to about 33.4% and 33.4% (*p* < 0.001). These results suggest that L1CAM affects the cell cycle and alters cell proliferation in human endometrial cancer. In HHUA cells, *L1CAM* knockdown tended to reduce the G2/M population; however, no apparent changes were observed in the G0/G1 phase (Supplementary Figure S3). Notably, no increase in apoptotic cells was observed upon *L1CAM* knockdown in either HHUA or SPAC-1-L (Figure 2C).

**Figure 3:**
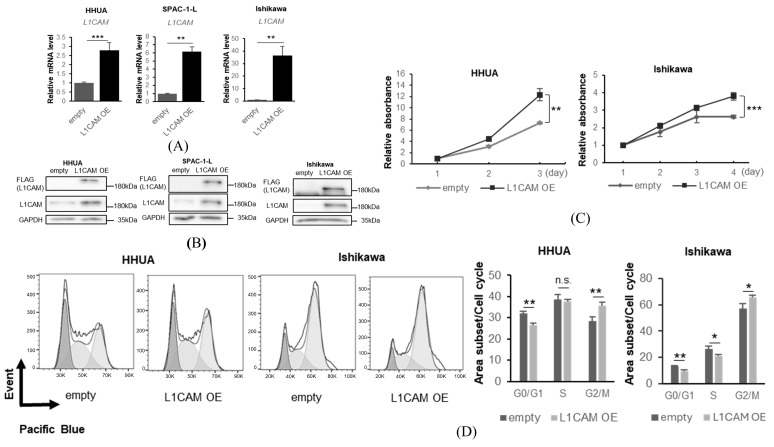
L1CAM overexpression induces the opposite effects of knockdown. (**A**) mRNA levels of *L1CAM* in HHUA, SPAC-1-L, and Ishikawa with control or *L1CAM* overexpression. *GAPDH* was used as an internal control. (**B**) Western blotting of FLAG, L1CAM, and GAPDH in HHUA, SPAC-1-L, and Ishikawa with control or *L1CAM* overexpression. GAPDH was used as a loading control. (**C**) MTT assay of HHUA and Ishikawa with control or *L1CAM* overexpression. (**D**) Cell cycle assay of HHUA and Ishikawa with control or *L1CAM* overexpression (*p* < 0.05 *, *p* < 0.01 **, *p* < 0.001 ***, n.s.: not significant). The uncropped blots are shown in Supplementary Figure S1.

Next, we used a lentiviral vector expressing *L1CAM* to generate cell lines that overexpressed FLAG-tagged L1CAM. Real-time PCR and Western blotting confirmed overexpression of both mRNA and protein (Figure 3A,B). Overexpression of *L1CAM* resulted in increased proliferation in HHUA and Ishikawa cell lines, with significant differences detected on day 3 in HHUA and day 4 in Ishikawa (Figure 3C). Cell cycle assays also revealed that overexpression of *L1CAM* promoted progression from the G0/G1 phase (HHUA 32.2% vs. 26.3%, Ishikawa 10.1% vs. 7.6%) (Figure 3D).

These results indicate that L1CAM expression in human endometrial cancer cells increases cell number by affecting the cell cycle but not apoptosis.

### 3.3. L1CAM Affects the NF-κB Signaling Pathway in Human Endometrial Cancer Cell Lines

To elucidate the mechanism by which L1CAM affects cell cycle and cell proliferation, bulk RNA-seq was performed on HHUA cells with knockdown of *L1CAM* to extract signaling pathways that were thought to be affected by KEGG pathway analysis (Table 1).

**Figure 4:**
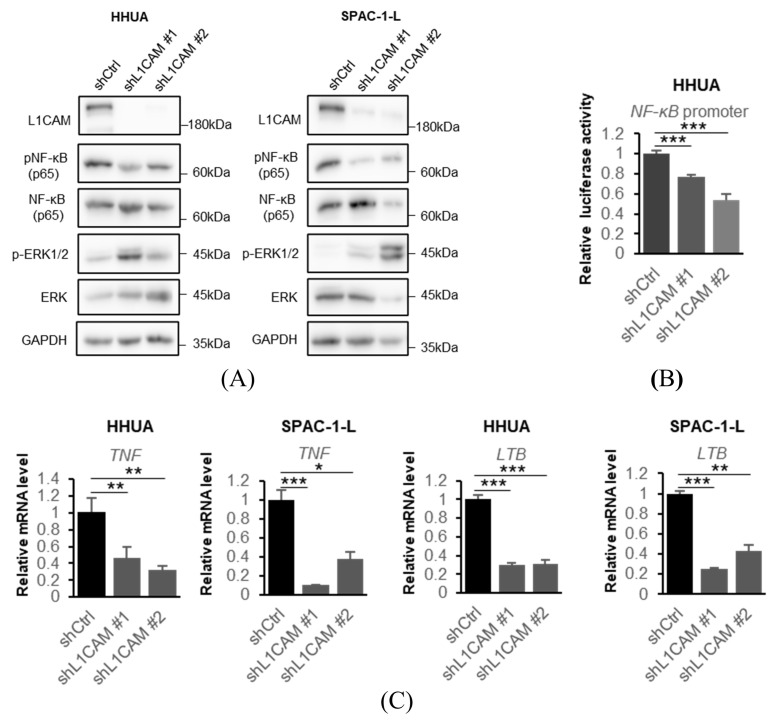
L1CAM knockdown suppresses the NF-κB signaling pathway. (**A**) Western blotting of L1CAM, pNF-κB (p65), NF-κB (p65), p-ERK1/2, ERK and GAPDH in HHUA and SPAC-1-L with control or L1CAM knockdown. GAPDH was used as a loading control. (**B**) Relative luciferase activity driven by the NF-κB promoter in HHUA cells with control or *L1CAM* knockdown. (**C**) Relative mRNA levels of *TNF* and *LTB* in HHUA and SPAC-1-L with control or *L1CAM* knockdown. *GAPDH* was used as an internal control. (*p* < 0.05 *, *p* < 0.01 **, *p* < 0.001 ***). The uncropped blots are shown in Supplementary Figure S1.

Based on the results of our RNA-seq data and previous studies conducted in other types of cancers [14,24,25], we focused our investigation on the NF-κB signaling pathway which is a very important signaling pathway in cancer [26]. We confirmed that the knockdown of L1CAM decreased phosphorylation of NF-κB (p65) in both HHUA and SPAC-1-L cell lines. (Figure 4A). On the other hand, unlike previous reports in pancreatic cancer, no correlation with ERK1/2 phosphorylation was observed [27] (Figure 4A). Luciferase reporter assays confirmed that knockdown of *L1CAM* reduced *NF-κB* promoter activity in HHUA cells (Figure 4B).

To investigate the relationship between L1CAM and the NF-κB signaling pathway, we evaluated NF-κB pathway-related mRNAs by real-time PCR. In HHUA and SPAC-1-L, knockdown of *L1CAM* resulted in decreased mRNA expression of *TNF* and *lymphotoxin beta* (*LTB)*, both target genes of NF-κB (Figure 4C) [28,29].

**Figure 5:**
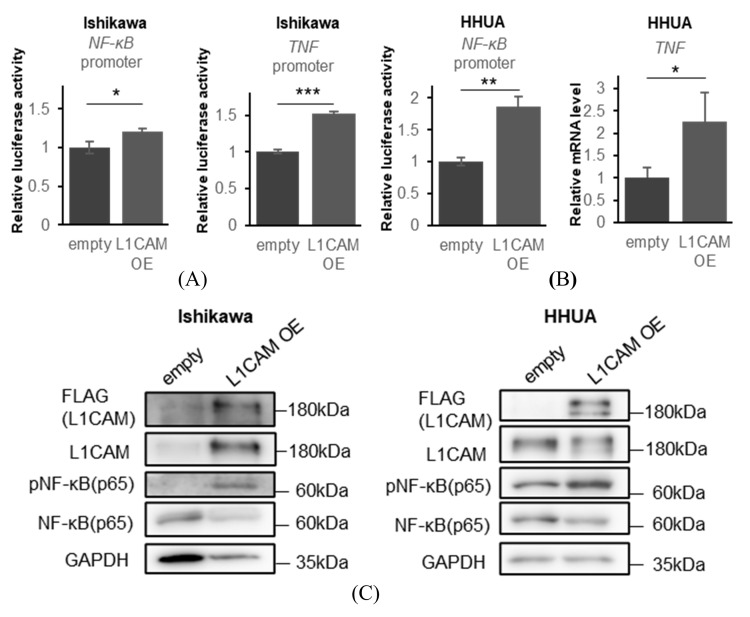
L1CAM overexpression activates the NF-kB signaling pathway. (**A**) Relative luciferase activities driven by the *NF-κB* and *TNF* promoters in Ishikawa cells. (**B**) Relative luciferase activities driven by the *NF-κB* promoter and relative *TNF* mRNA levels in HHUA cells. *GAPDH* was used as an internal control. (**C**) Western blotting of FLAG, L1CAM, pNF-κB (p65), NF-κB (p65) and GAPDH in Ishikawa and HHUA with control or L1CAM overexpression.GAPDH was used as a loading control. (*p* < 0.05 *, *p* < 0.01 **, *p* < 0.001 ***). The uncropped blots are shown in Supplementary Figure S1.

In contrast, overexpression of *L1CAM* increased expression of the *NF-κB* promoter-driven luciferase in Ishikawa and HHUA (Figure 5A,B). Increased expression of the *TNF* promoter-driven luciferase was also observed in Ishikawa (Figure 5A), whereas elevated *TNF* mRNA was confirmed by real-time PCR in HHUA cells (Figure 5B). We confirmed that overexpression of L1CAM enhanced phosphorylation of NF-κB (p65) in both Ishikawa and HHUA (Figure 5C).

These results suggest that L1CAM activates the NF-κB pathway and induces *TNF* mRNA expression in human endometrial cancer cell lines.

### 3.4. L1CAM Is Involved in the Proliferation of Human Endometrial Cancer Cells via NF-κB (p65) Activation

**Figure 6:**
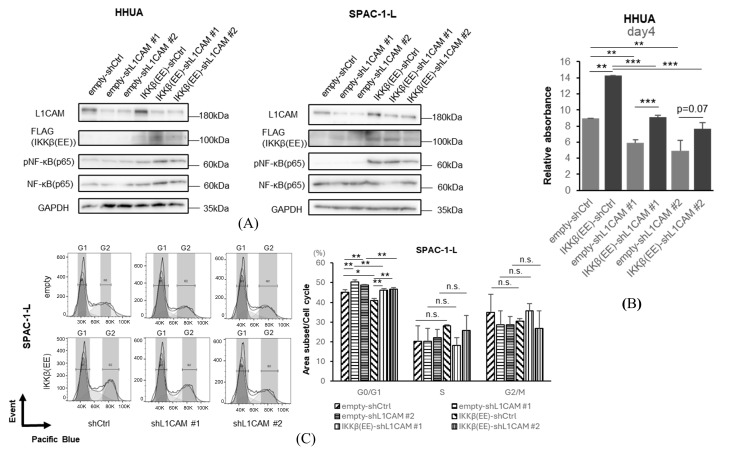
Activation of NF-κB rescues the phenotype induced by L1CAM knockdown. (**A**) Western blotting of L1CAM, FLAG, pNF-κB (p65), NF-κB (p65), and GAPDH in HHUA and SPAC-1-L with control or L1CAM knockdown with or without IKKβ(EE) overexpression. GAPDH was used as a loading control. (**B**) MTT assay of HHUA on day 4 with control or *L1CAM* knockdown with or without IKKβ(EE) overexpression. (**C**) Cell cycle assay of SPAC-1-L with control or *L1CAM* knockdown with or without IKKβ(EE) overexpression (*p* < 0.05 *, *p* < 0.01 **, *p* < 0.001 ***, n.s.: not significant). The uncropped blots are shown in Supplementary Figure S1.

Next, we performed rescue experiments with NF-κB (p65) activation to examine the effect of NF-κB (p65) activation on L1CAM-induced proliferation in human endometrial cancer cell lines. In HHUA and SPAC-1-L, lentiviral overexpression of IKKβ(EE), a constitutively active IKKβ, induced phosphorylation of NF-κB (p65) under L1CAM knockdown conditions (Figure 6A).

We confirmed that activation of NF-κB (p65) by IKKβ(EE) promotes cell proliferation (Figure 6B). This difference became significant on day 4, and the proliferation was recovered by activating IKKβ even though *L1CAM* was knocked down (Figure 6B). Activation of IKKβ(EE) with SPAC-1-L decreased the G0/G1 phase in both the control and *L1CAM* knockdown groups, suggesting that the cell cycle progression is promoted by increased phosphorylation of NF-κB (p65) (Figure 6C).

These results further reinforce that the effect of L1CAM on human endometrial cancer cells depends on the activation of the NF-κB (p65) pathway.

### 3.5. Correlation Between L1CAM Expression and NF-κB Expression in Patients

**Figure 7:**
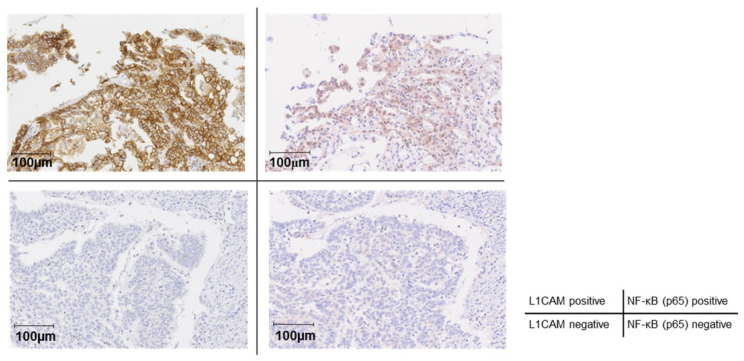
Correlation between L1CAM expression and NF-κB activation in human endometrial cancer patients. Immunohistochemical staining of L1CAM and NF-κB (p65) in TMA. The left and right panels in the upper row (double-positive for L1CAM and NF-κB [p65]) and those in the lower row (double-negative for L1CAM and NF-κB [p65]) represent paired sections derived from the same patients. The brown signal represents DAB staining, while the blue signal corresponds to hematoxylin counterstaining. The Original images of microscopy images are shown in Supplementary Figure S2.

To further confirm the relationship between L1CAM and the NF-κB pathway, we performed an evaluation using human clinical specimens. Immunohistochemical staining for L1CAM and NF-κB (p65) was performed in TMA specimens prepared from surgical specimens of human endometrial cancer; nuclear NF-κB (p65) positivity was significantly higher in L1CAM-positive cases, as determined by the chi-square test (*p* < 0.01) (Figure 7 and Table 2).

This result suggests that the activation of NF-κB (p65) by L1CAM expression also occurs in human endometrial cancer tissues.

### 3.6. IKK Inhibitors Suppress the Growth of Human Endometrial Cancer

**Figure 8:**
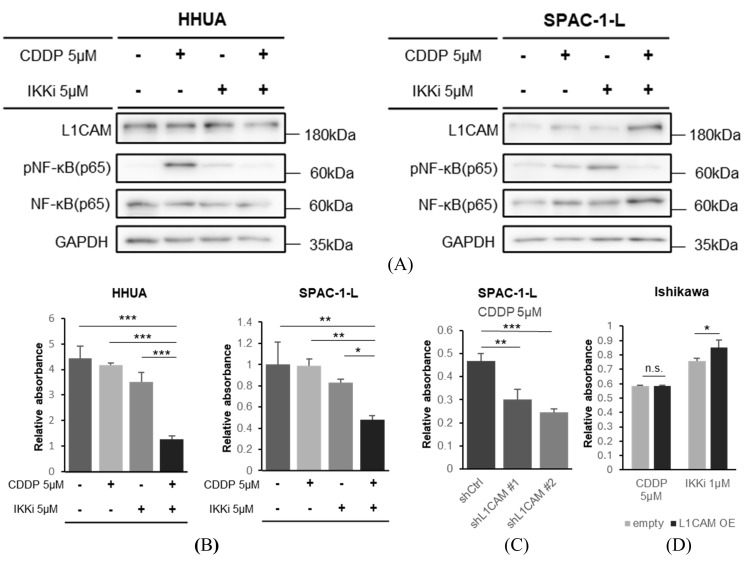
The combination of NF-κB inhibition and cisplatin more effectively inhibits proliferation of human endometrial cancer cells. (**A**) Western blotting of L1CAM, pNF-κB (p65), NF-κB (p65), and GAPDH in HHUA with drug addition. GAPDH was used as a loading control. (**B**) MTT assay of HHUA and SPAC-1-L cells were performed 48 h after treatment with cisplatin(CDDP), an IKK inhibitor, or their combination. (**C**) MTT assay of SPAC-1-L with control or *L1CAM* knockdown evaluated 48 h after cisplatin (CDDP) addition. (**D**) MTT assay of Ishikawa cells with control or *L1CAM* overexpression, evaluated 48 h after the addition of cisplatin (CDDP) or an IKK inhibitor. (*p* < 0.05 *, *p* < 0.01 **, *p* < 0.001 ***, n.s.: not significant). The uncropped blots are shown in Supplementary Figure S1.

Finally, we confirmed the association between L1CAM expression and chemotherapy resistance. We found that cisplatin treatment of HHUA and SPAC-1-L promoted NF-κB (p65) phosphorylation. This effect was suppressed by the concomitant use of an IKK inhibitor (Figure 8A).

In drug sensitivity experiments in HHUA and SPAC-1-L, cisplatin in combination with an IKK inhibitor inhibited cell proliferation more strongly at 48 h after drug administration than cisplatin monotherapy or IKK inhibitor monotherapy (Figure 8B). This is also indicated by stronger growth inhibition observed in SPAC-1-L with *L1CAM* knockdown in response to cisplatin monotherapy (Figure 8C). Conversely, the growth suppression of Ishikawa cells overexpressing *L1CAM* was attenuated when treated with IKK inhibitors; cisplatin did not change that (Figure 8D).

These results suggest that increased expression of L1CAM promotes the activation of NF-κB (p65) and contributes to drug resistance, and that suppressing NF-κB (p65) may be effective in chemotherapy against human endometrial cancer [26,30,31].

## 4. Discussion

L1CAM is a poor prognostic factor in endometrial cancer, and many clinical studies have reported that L1CAM expression is associated with drug resistance [15,16,18,19,21,22]. However, the underlying mechanisms remain unclear. This study demonstrated that L1CAM is involved in cell proliferation and transition from the G0/G1 phase using human endometrial cancer cell lines with reduced L1CAM expression. Conversely, when L1CAM expression was increased, the opposite changes were observed. These changes suggest that L1CAM plays a role in controlling cell proliferation by acting on the G0/G1 checkpoint in human endometrial cancer. Based on the report on the relationship between L1CAM and NF-κB in pancreatic cancer and our RNA-seq results, we focused on the NF-κB pathway [14]. In this study, we primarily focused on RelA (p65) as the downstream effector of NF-κB signaling. RelA is widely recognized as the functionally dominant NF-κB subunit in cancer biology, serving as the principal mediator of canonical NF-κB activation and regulating transcriptional programs involved in tumor proliferation, survival, inflammation, and chemoresistance. This central role has been well documented in multiple cancer types [26]. Furthermore, a recent systematic review on endometrial diseases highlighted that research in endometrial carcinoma has predominantly focused on canonical NF-κB signaling, with RelA being the most frequently investigated subunit [32]. This trend reflects its recognized involvement in promoting cell proliferation, survival, and inflammatory signaling in endometrial cancer, supporting our decision to prioritize RelA in the present work. While our findings suggest that RelA represents a major oncogenic NF-κB output in this context, other NF-κB subunits, including RelB, c-Rel, p50, and p52, may also contribute to the phenotype in a subunit-specific or context-dependent manner. Future studies will be required to determine whether alternative NF-κB dimers participate in L1CAM-mediated signaling and to provide a more comprehensive understanding of NF-κB regulation in endometrial cancer. We clarified that L1CAM promotes NF-κB (p65) phosphorylation in human endometrial cancer. Furthermore, we demonstrated that NF-κB (p65) phosphorylation itself positively influences the transition from the G0/G1 phase in human endometrial cancer. The upregulation of *TNF* and *LTB* mRNA levels suggests that activation of the L1CAM-NF-κB pathway may activate a positive feedback mechanism, i.e., an autocrine mechanism, in the TNF-NF-κB pathway, thereby maintaining NF-κB activation. These findings indicate that the L1CAM-NF-κB (p65) pathway may be involved in the poor prognosis of human endometrial cancer, consistent with previous reports showing that high expression of TNF-α is associated with poor prognosis [33]. We did not directly assess whether L1CAM regulates NF-κB (p65) activation through an autocrine cytokine loop. Measuring TNF-α levels in the supernatant under L1CAM overexpression or inhibition conditions, or testing whether conditioned media from high-L1CAM-expressing cells can induce NF-κB activation in low-L1CAM-expressing lines, would help clarify this possibility. Although such experiments were beyond the scope of the present study, they represent an important direction for future studies to determine whether an autocrine L1CAM–TNF-α–NF-κB signaling axis contributes to the observed phenotypes. Our results show that the combination of cisplatin and IKK inhibitors has a stronger inhibitory effect on cell proliferation, and suggest that the L1CAM-NF-κB (p65) pathway is involved in drug resistance in human endometrial cancer.

This study has several limitations. Firstly, the number of human endometrial cancer cell lines used was limited. In addition, some of the assays did not yield sufficiently precise or reproducible results to be included in the final analysis. For this reason, we selected the cell lines that produced technically reliable and interpretable data for each specific assay. Although we used human endometrial cancer cell lines with different mutations, further validation combining TCGA classification is necessary to obtain more clinically useful data. Another limitation is that while we confirmed that L1CAM-induced NF-κB (p65) activation occurs not only in vitro but also in vivo based on TMA evaluation, further validation is needed to determine whether similar changes occur in vivo. We did not investigate potential interactions between L1CAM and integrins. Previous work in pancreatic cancer has demonstrated that L1CAM can activate NF-κB through binding to specific integrins, suggesting that integrin-mediated signaling may contribute to L1CAM-dependent NF-κB activation in certain tumor types [12]. Although our data support a functional link between L1CAM and the NF-κB (p65) pathway in human endometrial cancer cells, the possibility that integrins participate in this signaling axis cannot be excluded. Future studies will be required to determine whether L1CAM–integrin interactions occur in human endometrial cancer and whether they modulate NF-κB activation in a cell-type-specific manner.

An additional consideration in interpreting our findings is the unexpected spindle-shaped morphology observed after *L1CAM* knockdown. Given the established role of L1CAM in promoting EMT, a loss of L1CAM would typically be expected to drive cells toward a more epithelial appearance rather than a fusiform phenotype. This morphological change may therefore reflect stress-induced senescence or an abnormal differentiation state rather than a straightforward reversal of EMT. Cytoskeletal immunofluorescence would be the appropriate approach to clarify the underlying structural alterations, but it has not been performed. Future studies will be required to determine whether L1CAM depletion induces senescence-associated remodeling or alters cytoskeletal dynamics in a manner that impacts cellular behavior.

Paradoxical increases in pNF-κB (p65) after IKKβ inhibition have been reported and are attributed to IKKβ-independent phosphorylation mediated by MAPK and other stress-responsive kinases [34]. The strength of this compensatory signaling may differ across cell lines. Thus, the elevated pNF-κB (p65) in SPAC-1-L likely reflects a cell-line–specific compensatory mechanism, rather than activation of NF-κB (p65) by IKKβ inhibitor. Further verification is also necessary on this point.

Finally, another important consideration is the biological interpretation of the cellular response to L1CAM knockdown. While L1CAM has been implicated in promoting chemoresistance, our data indicate cell-cycle arrest rather than apoptosis following its silencing. Because cisplatin sensitivity is typically mediated through apoptosis induction, these findings suggest that L1CAM loss may not simply reverse a classical chemoresistance mechanism. The spindle-shaped morphology observed in knockdown cells raises the possibility that they may instead be entering a senescence-like state or undergoing cytoskeletal remodeling associated with altered migratory behavior. Although additional assays would be required to distinguish between these possibilities, these alternative interpretations should be considered when evaluating the functional consequences of L1CAM depletion. Future studies will be needed to clarify whether L1CAM regulates apoptosis, senescence, or cell motility in a context-dependent manner.

Our results suggest the potential for new treatment methods targeting L1CAM for human endometrial cancer, as well as the possibility that inhibiting the NF-κB (p65) signaling pathway in L1CAM-positive tumors may contribute to improved prognosis.

## 5. Conclusions

In this study, we demonstrate for the first time that the L1CAM drives proliferation and chemoresistance in human endometrial cancer through activation of the NF-κB (p65) signaling pathway. Functional analyses revealed that L1CAM promotes G0/G1 cell-cycle transition and proliferation, while its knockdown inhibits NF-κB (p65) activation and sensitizes cancer cells to cisplatin. Importantly, our analysis of patient samples revealed a significant correlation between L1CAM expression and nuclear NF-κB (p65) positivity, and combined therapy with cisplatin and an IKK inhibitor synergistically enhanced anti-proliferative effects in vitro. These findings establish a novel mechanistic link between L1CAM and NF-κB signaling in endometrial cancer progression and suggest that targeting the L1CAM-NF-κB axis may represent a promising therapeutic approach for patients with L1CAM-positive tumors.

## Figures and Tables

**Table 1:** Analysis of HHUA with control or *L1CAM* knockdown RNA-seq data using DAVID.

Term	Count	%	*p*-Value
Pathways in cancer	47	2.5	5.50 × 10^−3^
Neuroactive ligand-receptor interaction	39	2.1	5.00 × 10^−4^
Cytokine-cytokine receptor interaction	32	1.7	1.50 × 10^−3^
MAPK signaling pathway	32	1.7	1.60 × 10^−3^
PI3K-Akt signaling pathway	31	1.7	3.80 × 10^−2^
Calcium signaling pathway	30	1.6	4.70 × 10^−4^
Cell adhesion molecules	25	1.3	1.50 × 10^−5^
Ras signaling pathway	23	1.2	2.40 × 10^−2^
cAMP signaling pathway	22	1.2	2.60 × 10^−2^
Rap1 signaling pathway	20	1.1	4.50 × 10^−2^
Cytoskeleton in muscle cells	20	1.1	9.20 × 10^−2^
**NF-kappa B signaling pathway**	19	1	3.70 × 10^−5^
Transcriptional misregulation in cancer	19	1	3.60 × 10^−2^
Focal adhesion	19	1	5.50 × 10^−2^
Proteoglycans in cancer	19	1	5.70 × 10^−2^
Rheumatoid arthritis	18	1	3.00 × 10^−5^
JAK-STAT signaling pathway	18	1	2.10 × 10^−2^
Axon guidance	17	0.9	7.70 × 10^−2^
Parathyroid hormone synthesis, secretion and action	16	0.9	3.00 × 10^−3^
**TNF signaling pathway**	16	0.9	4.20 × 10^−3^

**Table 2:** Number of L1CAM-positive cells and NF-κB (p65)-positive nuclei.

	NF-κB (p65) +	NF-κB (p65) −	Total
**L1CAM +**	45	135	180
**L1CAM −**	21	159	180
**Total**	66	294	360

## Data Availability

RNA-seq data are available in GEO under accession number GSE299250.

## Supplementary Materials

The following supporting information can be downloaded at: https://www.mdpi.com/article/10.3390/cancers18020198/s1, Table S1: Sequence of shRNA. Table S2: Sequence of qPCR primers. Table S3: Sequence of luciferase assay reporter plasmid. Table S4: Mutation of cell lines. Figure S1: Original images of electrophoretic blots. Figure S2: Original images of microscopy images. Figure S3: Cell cycle assay of HHUA with control or L1CAM knockdown.

## Abbreviations

The following abbreviations are used in this manuscript:

CDDP	cisplatin
DAVID	Database for Annotation, Visualization, and Integrated Discovery
FIGO	The International Federation of Gynecology and Obstetrics
IKK	I kappa B kinase
KEGG	Kyoto Encyclopedia of Genes and Genomes
L1CAM	L1 cell adhesion molecule
LTB	lymphotoxin beta
MSI	microsatellite instability
PI	propidium iodide
RIN	RNA Integrity Number
TCGA	The Cancer Genome Atlas
TMA	tissue microarray
TNF	tumor necrosis factor
