# Supplementary material for: L1CAM Promotes Human Endometrial Cancer Via NF-κB Activation
Source: Cancers (Basel). 2026 Jan 8;18(2):198. doi: 10.3390/cancers18020198 (PMC12839394; doi:10.3390/cancers18020198)
Supplement: Supplementary file 1 [file cancers-18-00198-s001.zip › Supplementary Table S1 Sequence of shRNA.pdf]

Supplementary Table S1

Sequence of shRNA

	Target Sequence	Target location	Clone ID
shL1CAM #1	GCCAATGCCTACATCTACGTT	1351-1371	TRCN0000299624
shL1CAM #2	GTCTCCGAAATGCTGTCTTTC	4173-4193	TRCN0000310751
