# Supplementary material for: L1CAM Promotes Human Endometrial Cancer Via NF-κB Activation
Source: Cancers (Basel). 2026 Jan 8;18(2):198. doi: 10.3390/cancers18020198 (PMC12839394; doi:10.3390/cancers18020198)
Supplement: Supplementary file 1 [file cancers-18-00198-s001.zip › Supplementary Table S2 Sequence of qPCR primers.pdf]

Supplementary Table S2

Sequence of qPCR primers

Gene name	Forward primer	Reverse primer
L1CAM	5'-ACCGTGTCACCTACCAGAAC-3'	5'-CAGCGGTACTCGCCATCAT-3'
TNF	5'-GTAGCCCATGTTGTAGCAAAC-3'	5'-GGTTATCTCTCAGCTCCACG-3'
LTB	5'-GGAGACGACGAAGGAACAGG-3'	5'-GTAGAGGTAATAGAGGCCGTCC-3'
