# Supplementary material for: L1CAM Promotes Human Endometrial Cancer Via NF-κB Activation
Source: Cancers (Basel). 2026 Jan 8;18(2):198. doi: 10.3390/cancers18020198 (PMC12839394; doi:10.3390/cancers18020198)
Supplement: Supplementary file 1 [file cancers-18-00198-s001.zip › Supplementary Table S3 Sequence of luciferase assay reporter plasmid.pdf]

Supplementary Table S3

Sequence of luciferase assay reporter plasmid

Gene name	insert promoter region
TNF	CTGCACTCGATGTACCACGGGGCTGCGTTCCAGCTCACCCAGGGAGA<br>CCAGCTATCCACCCACACAGATGGCATCCCCCACCTAGTCCTCAGCCC<br>TAGTACTGTCTTCTTTGGAGCCTTCGCTCTGTAGAACTTGGAAAAATC<br>CAGAAAGAAAAAATAATTGATTTCAAGACCTTCTCCCCATTCTGCCTC<br>CATTCTGACCATTTCAGGGGTCGTCACCACCTCTCCTTTGGCCATTCC<br>AACAGCTCAAGTCTTCCCTGATCAAGTCACCGGAGCTTTCAAAGAAG<br>GAATTCTAGGCATCCCAGGGGACCACACCTCCCTGAACCATCCCTGA<br>TGTCTGTCTGGCTGAGGATTTCAAGCCTGCCTAGGAATTCCCAGCCCA<br>AAGCTGTTGGTCTGTCCCACCAGCTAGGTGGGGCCTAGATCCACACA<br>CAGAGGAAGAGCAGGCACATGGAGGAGCTTGGGGGATGACTAGAGG<br>CAGGGAGGGGACTATTTATGAAGGCAAAAAAATTAAATTATTTATTT<br>ATGGAGGATGGAGAGAGGGGAATAATAGAAGAACATCCAAGGAGAA<br>ACAGAGACAGGCCCAAGAGATGAAGAGTGAGAGGGCATGCGCACAA<br>GGCTGACCAAGAGAGAAAGAAGTAGGCATGAGGGATCACAGGGCCC<br>CAGAAGGCAGGGAAAGGCTCTGAAAGCCAGCTGCCGACCAGAGCCC<br>CACACGGAGGCATCTGCACCCTCGATGAAGCCCAATAAACCTCTTTT<br>CTCTGAAATGCTGTCTGCTTGTGTGTGTGTGTCTGGGAGTGAGAACTT<br>CCCAGTCTATCTAAGGAATGGAGGGAGGGACAGAGGGCTCAAAGGG<br>AGCAAGAGCTGTGGGGAGAACAAAAGGATAAGGGCTCAGAGAGCTT<br>CAGGGATATGTGATGGACTCACCAGGTGAGGCCGCCAGACTGCTGCA<br>GGGGAAGCAAAGGAGAAGCTGAGAAGATGAAGGAAAAGTCAGGGTC<br>TGGAGGGGCGGGGGTCAGGGAGCTCCTGGGAGATATGGCCACATGT<br>AGCGGCTCTGAGGAATGGGTTACAGGAGACCTCTGGGGAGATGTGAC<br>CACAGCAATGGGTAGGAGAATGTCCAGGGCTATGGAAGTCGAGTATG<br>GGGACCCCCCCTTAACGAAGACAGGGCCATGTAGAGGGCCCCAGGG<br>AGTGAAAGAGCCTCCAGGACCTCCAGGTATGGAATACAGGGGACGTT<br>TAAGAAGATATGGCCACACACTGGGGCCCTGAGAAGTGAGAGCTTCA<br>TGAAAAAAATCAGGGACCCCAGAGTTCCTTGGAAGCCAAGACTGAA<br>ACCAGCATTATGAGTCTCCGGGTCAGAATGAAAGAAGAAGGCCTGCC<br>CCAGTGGGGTCTGTGAATTCCCGGGGGTGATTTCACTCCCCGGGGCT

	GTCCCAGGCTTGTCCCTGCTACCCCCACCCAGCCTTTCCTGAGGCCTC<br>AAGCCTGCCACCAAGCCCCCAGCTCCTTCTCCCCGCAGGGACCCAAA<br>CACAGGCCTCAGGACTCAACACAGCTTTTCCCTCCAACCCCGTTTTCT<br>CTCCCTCAAGGACTCAGCTTTCTGAAGCCCCTCCCAGTTCTAGTTCTA<br>TCTTTTTCCTGCATCCTGTCTGGAAGTTAGAAGGAAACAGACCACAG<br>ACCTGGTCCCCAAAAGAAATGGAGGCAATAGGTTTTGAGGGGCATGG<br>GGACGGGGTTCAGCCTCCAGGGTCCTACACACAAATCAGTCAGTGGC<br>CCAGAAGACCCCCCTCGGAATCGGAGCAGGGAGGATGGGGAGTGTG<br>AGGGGTATCCTTGATGCTTGTGTGTCCCCAACTTTCCAAATCCCCGCC<br>CCCGCGATGGAGAAGAAACCGAGACAGAAGGTGCAGGGCCCACTAC<br>CGCTTCCTCCAGATGAGCTCATGGGTTTCTCCACCAAGGAAGTTTTCC<br>GCTGGTTGAATGATTCTTTCCCCGCCCTCCTCTCGCCCCAGGGACATA<br>TAAAGGCAGTTGTTGGCACACCCAGCC
