# Supplementary material for: L1CAM Promotes Human Endometrial Cancer Via NF-κB Activation
Source: Cancers (Basel). 2026 Jan 8;18(2):198. doi: 10.3390/cancers18020198 (PMC12839394; doi:10.3390/cancers18020198)
Supplement: Supplementary file 1 [file cancers-18-00198-s001.zip › Supplementary Table S4 Mutation of cell lines.pdf]

Supplementary Table S4

Mutation of cell lines

	Mutation
Ishikawa	*PIK3R1, POLE, PTEN, TP53*
HEC-1	*KRAS, MSH6, PMS2, TP53*
HHUA	*TP53*
SPAC-1-L, SPAC-1-S	*PTEN*
HOUA	*KRAS, TP53*
