# Supplementary figures and images for: L1CAM Promotes Human Endometrial Cancer Via NF-κB Activation
Source: Cancers (Basel). 2026 Jan 8;18(2):198. doi: 10.3390/cancers18020198 (PMC12839394; doi:10.3390/cancers18020198)

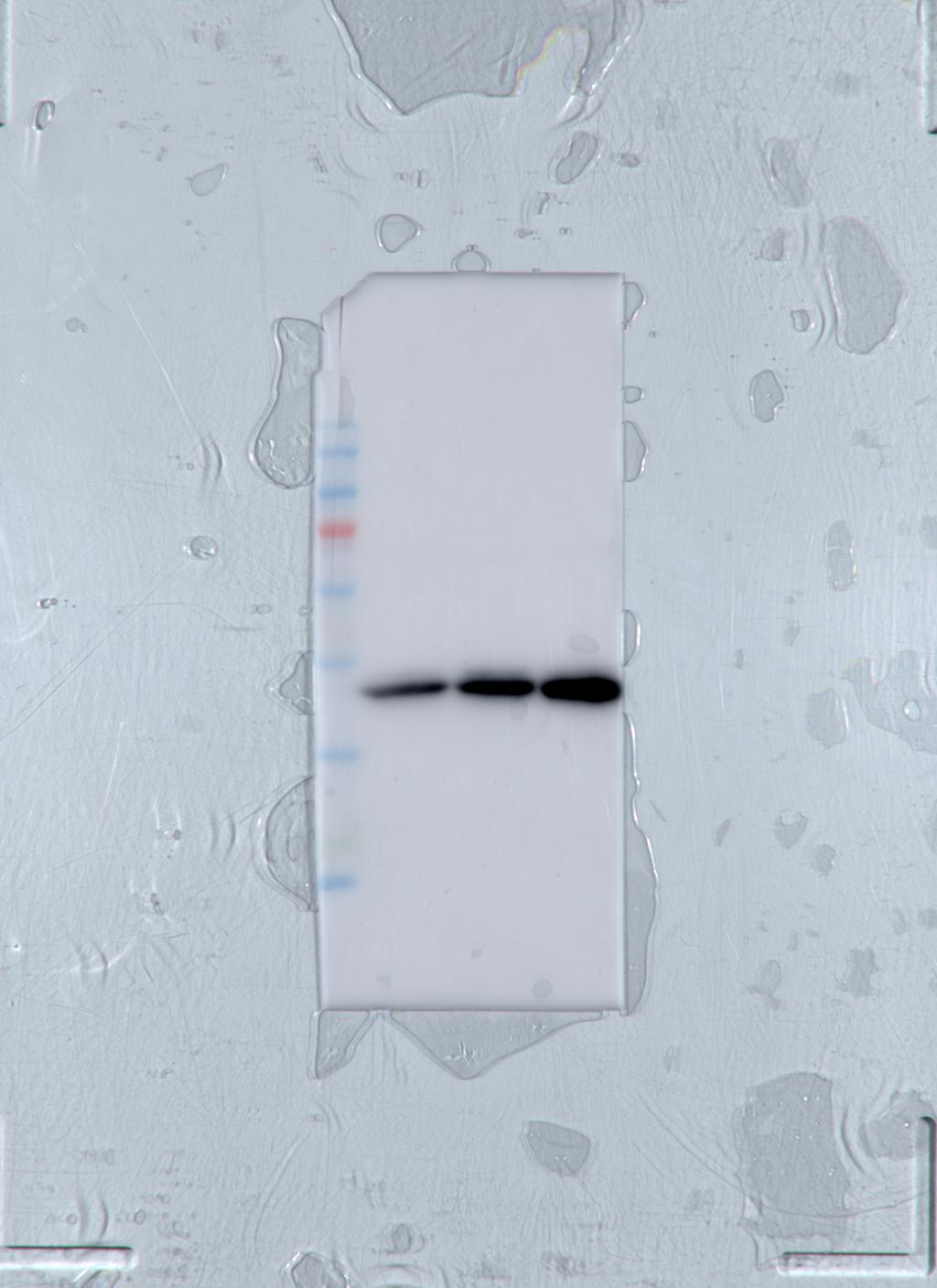

Supplement: Supplementary file 1 [file cancers-18-00198-s001.zip › Figure S1 and S2 Kurosu Original Images for Blots or Gels or Microscopy/Figure10 HHUA_ERK.tif]

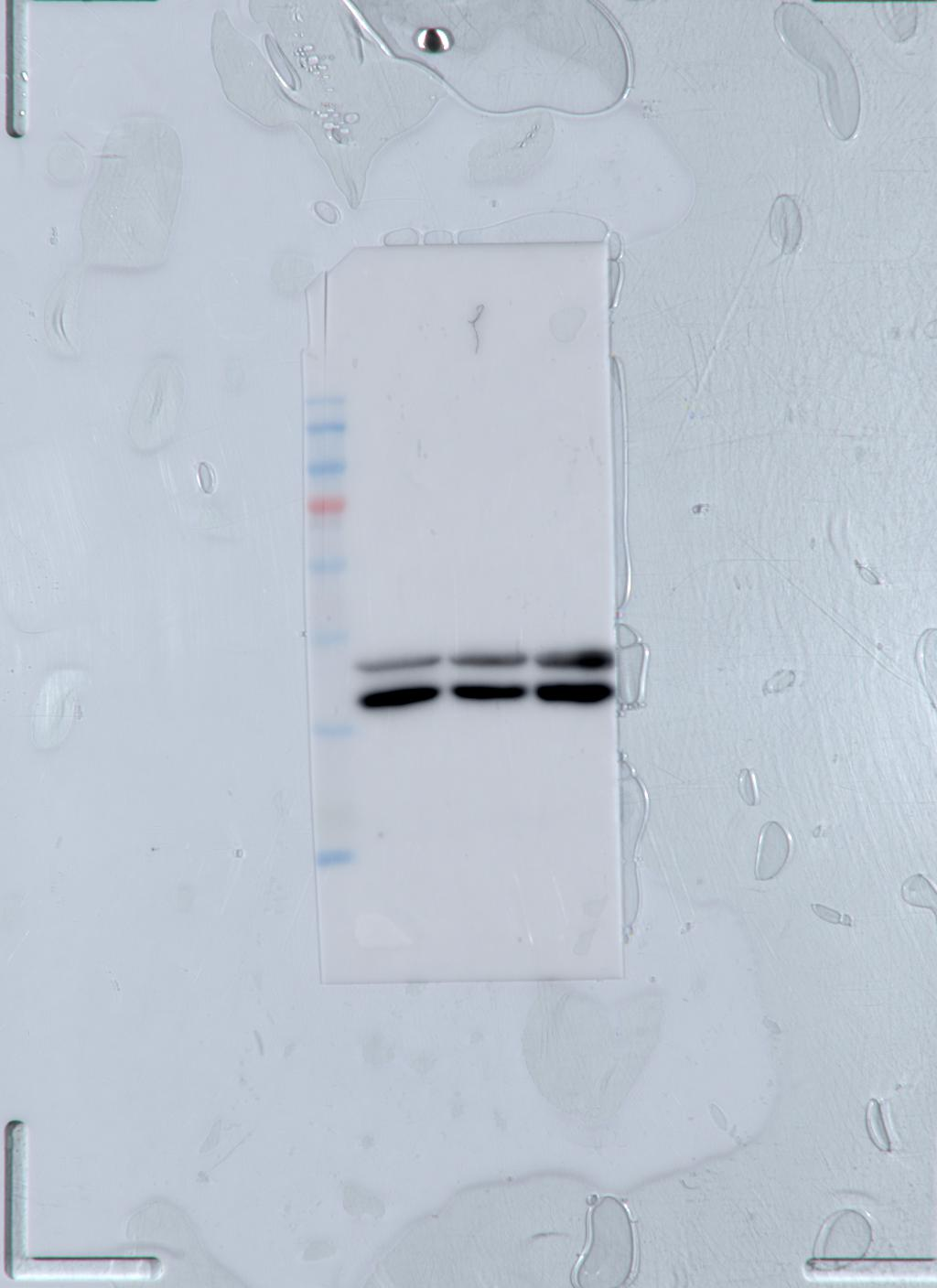

Supplement: Supplementary file 1 [file cancers-18-00198-s001.zip › Figure S1 and S2 Kurosu Original Images for Blots or Gels or Microscopy/Figure10 HHUA_GAPDH (Same membrane as ERK)(unused in figure10).tif]

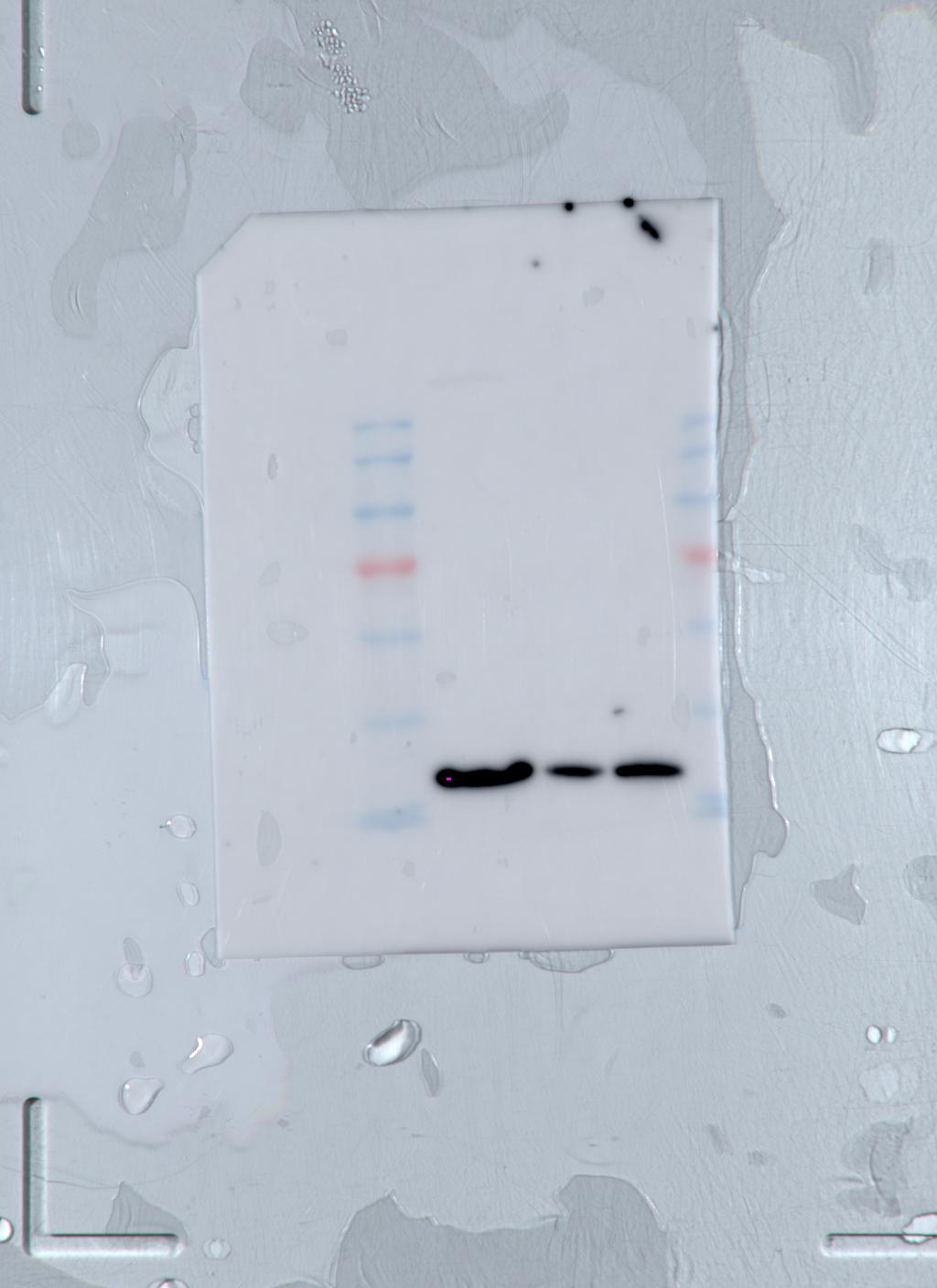

Supplement: Supplementary file 1 [file cancers-18-00198-s001.zip › Figure S1 and S2 Kurosu Original Images for Blots or Gels or Microscopy/Figure10 HHUA_GAPDH (Same membrane as L1CAM)(unused in figure10).tif]

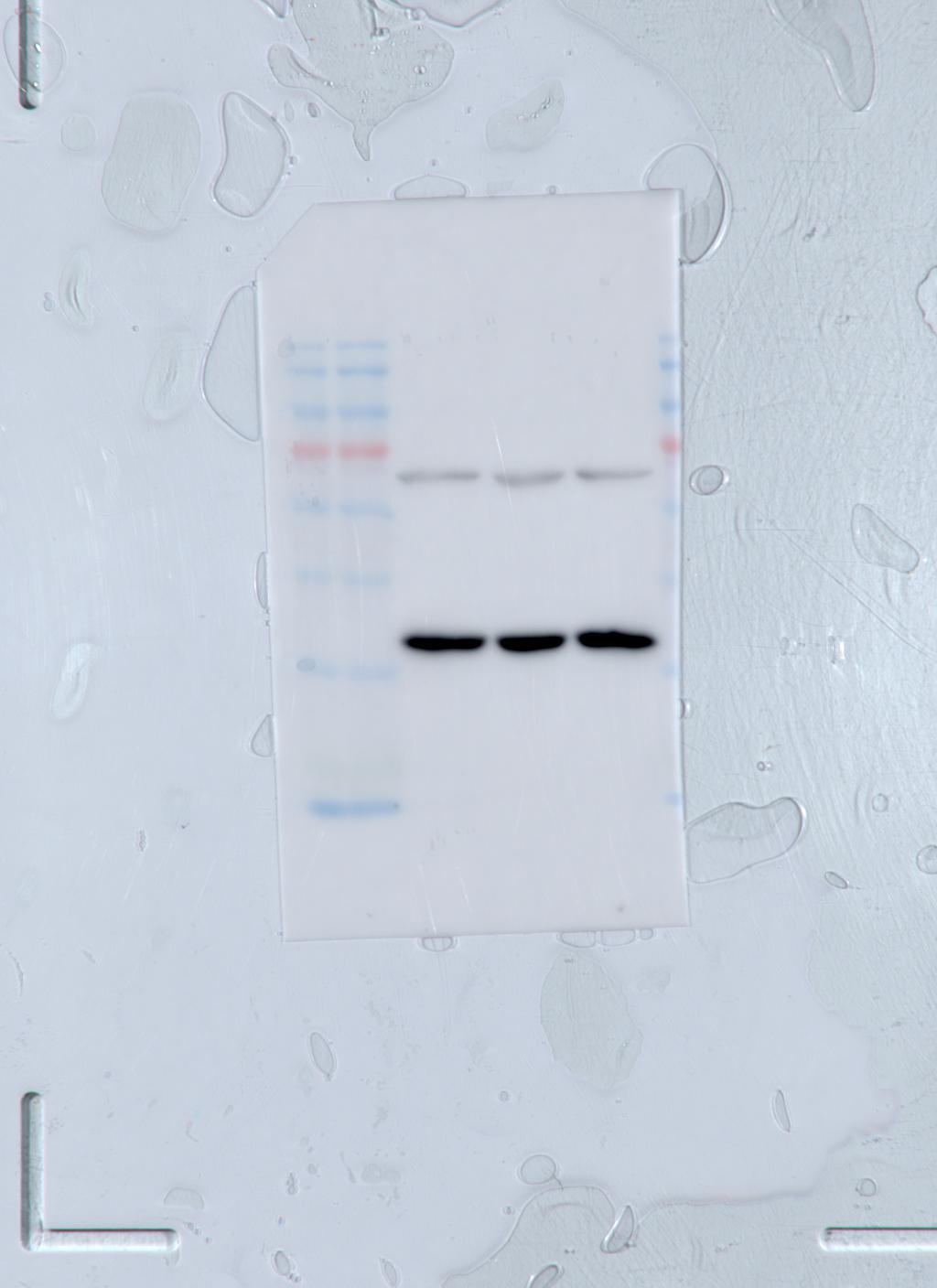

Supplement: Supplementary file 1 [file cancers-18-00198-s001.zip › Figure S1 and S2 Kurosu Original Images for Blots or Gels or Microscopy/Figure10 HHUA_GAPDH (Same membrane as NF-kB)(used in figure10).tif]

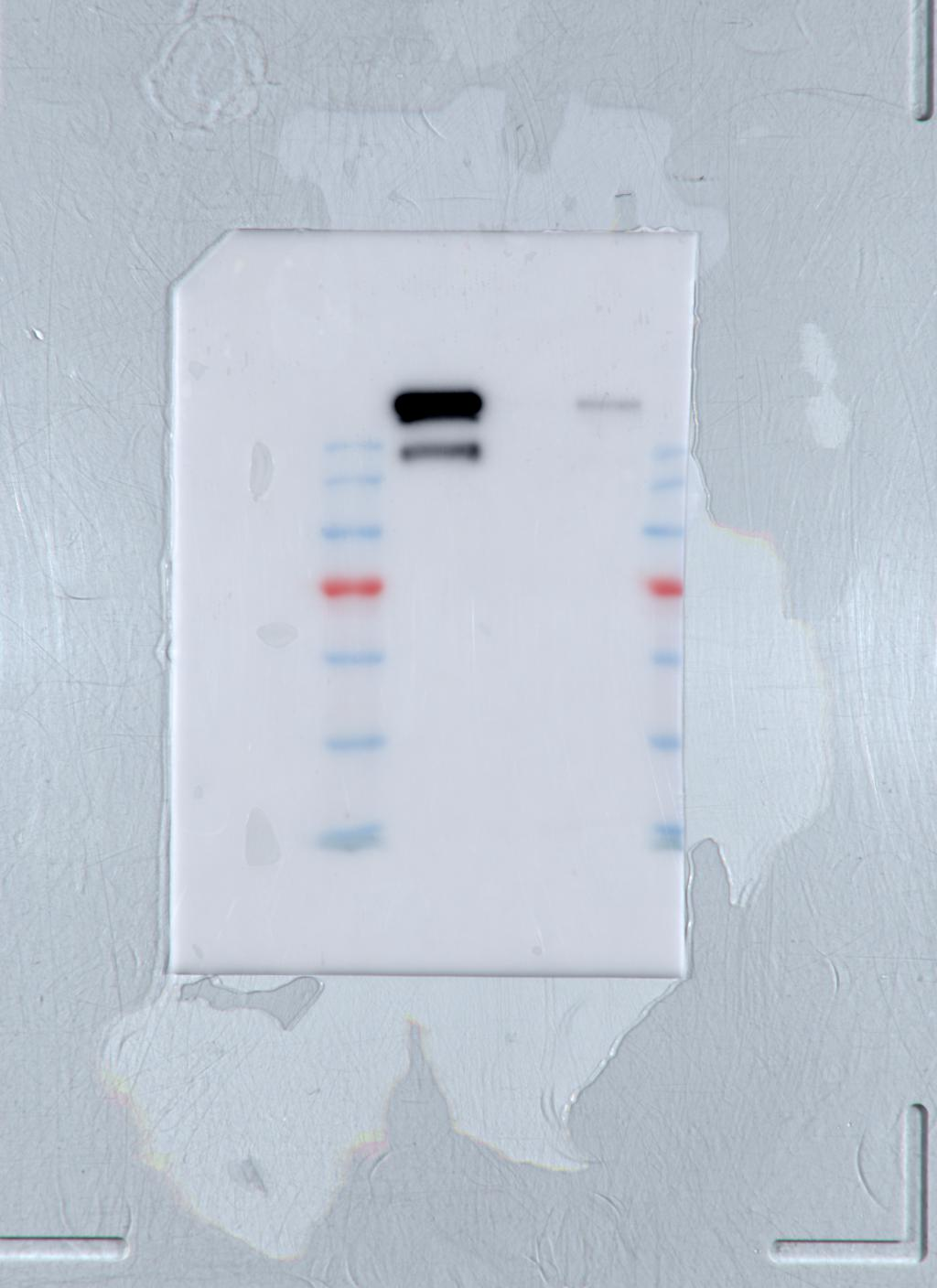

Supplement: Supplementary file 1 [file cancers-18-00198-s001.zip › Figure S1 and S2 Kurosu Original Images for Blots or Gels or Microscopy/Figure10 HHUA_L1CAM.tif]

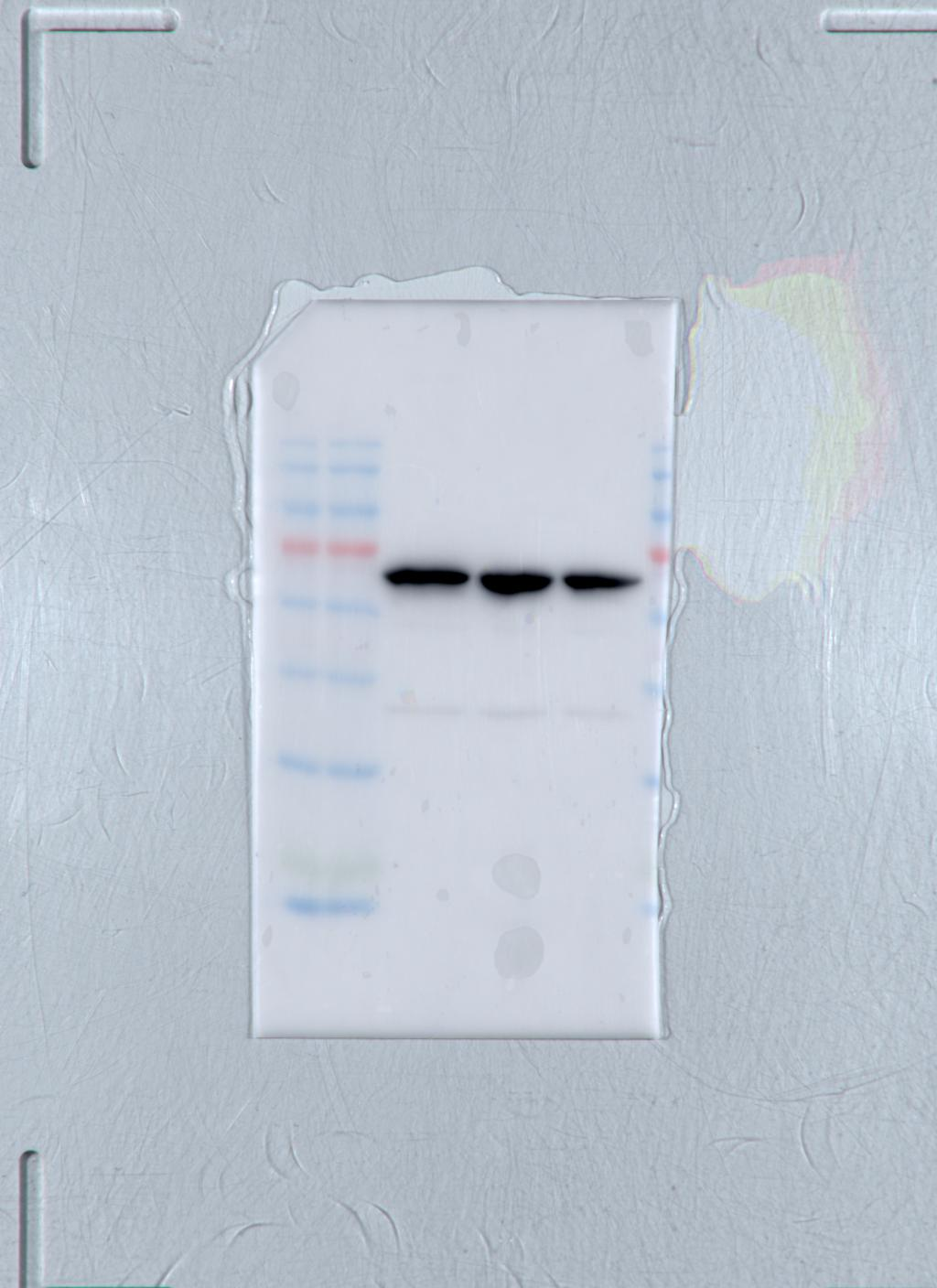

Supplement: Supplementary file 1 [file cancers-18-00198-s001.zip › Figure S1 and S2 Kurosu Original Images for Blots or Gels or Microscopy/Figure10 HHUA_NF-kB(p65).tif]

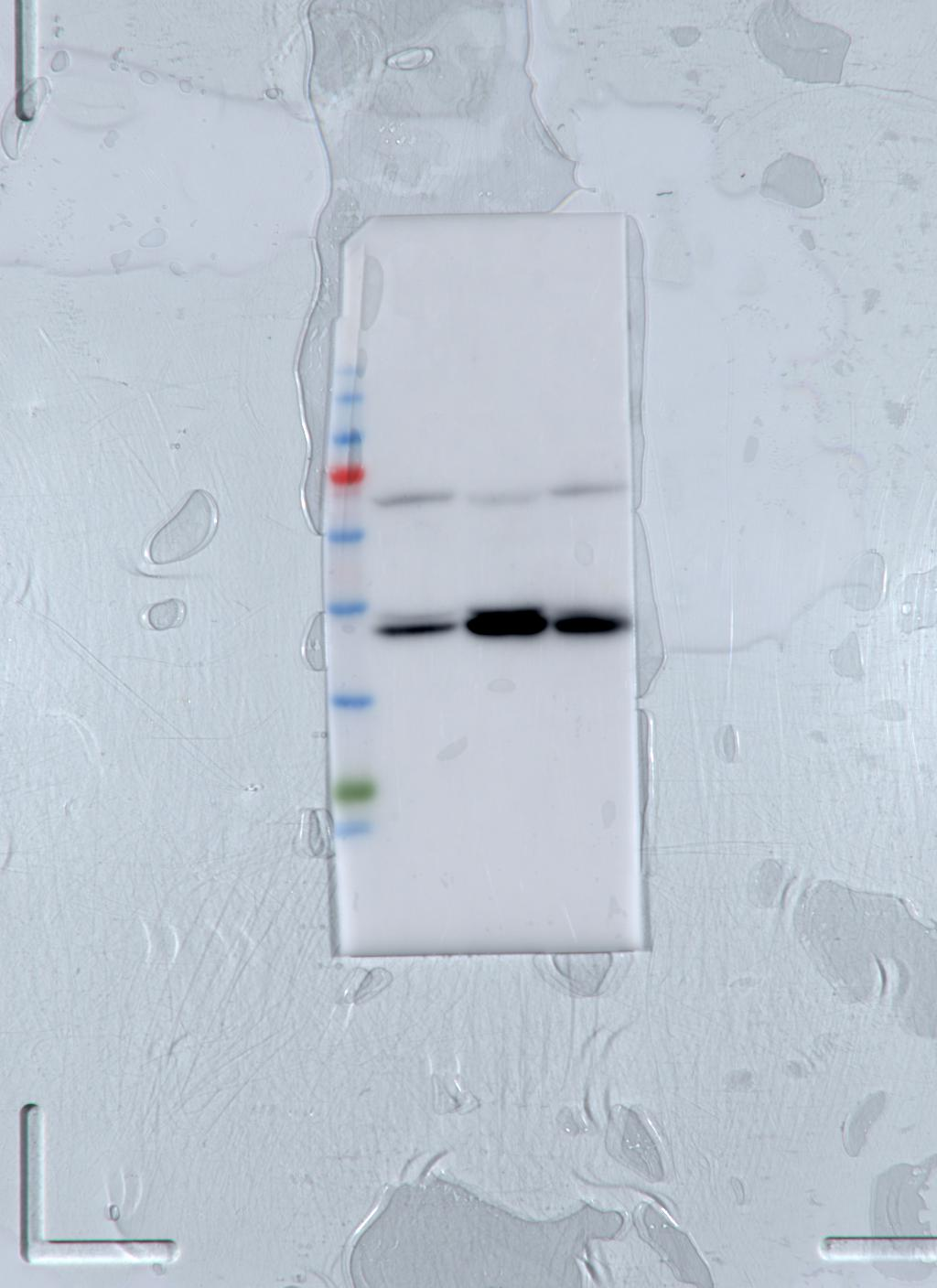

Supplement: Supplementary file 1 [file cancers-18-00198-s001.zip › Figure S1 and S2 Kurosu Original Images for Blots or Gels or Microscopy/Figure10 HHUA_pERK1_2.tif]

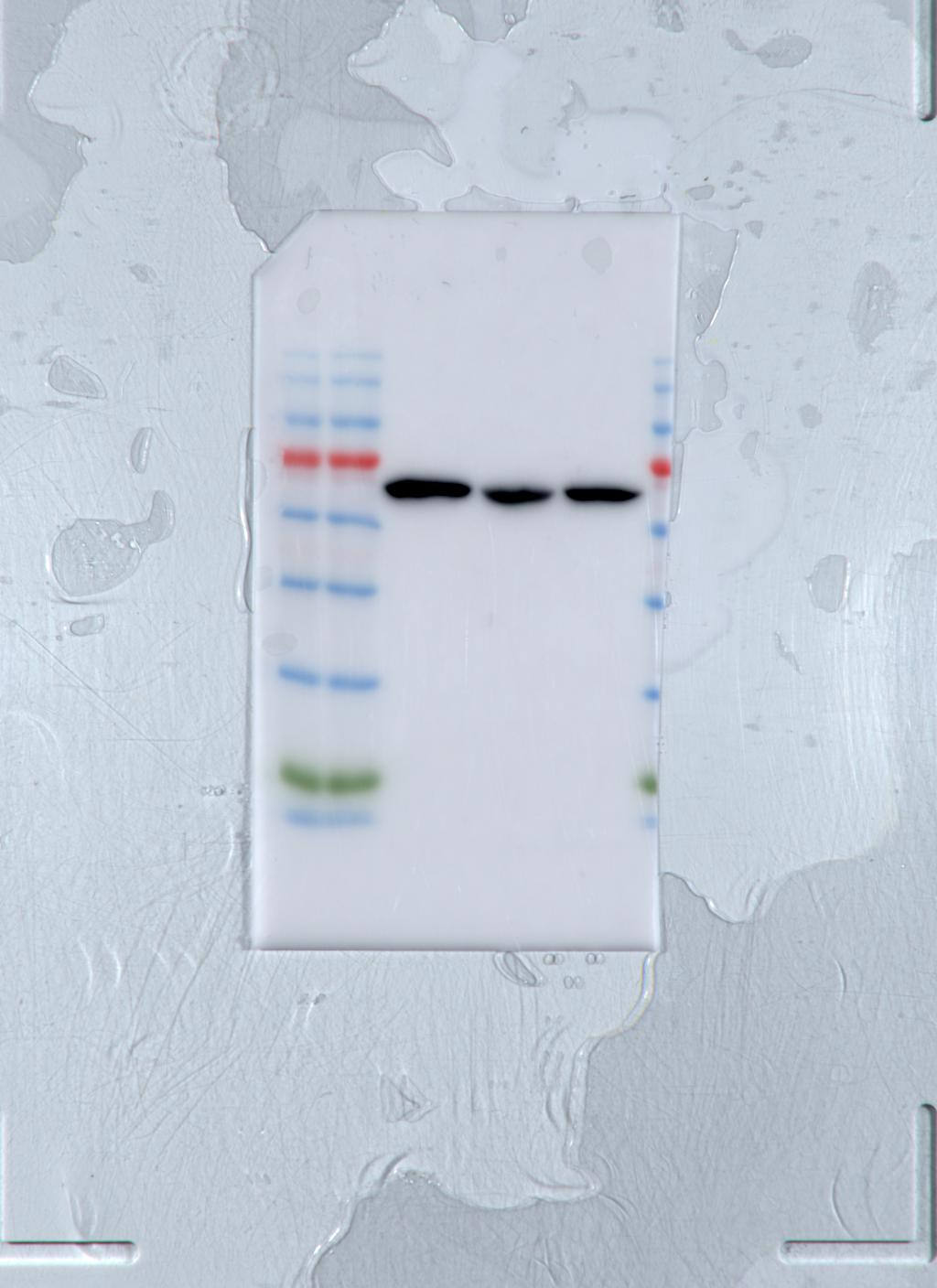

Supplement: Supplementary file 1 [file cancers-18-00198-s001.zip › Figure S1 and S2 Kurosu Original Images for Blots or Gels or Microscopy/Figure10 HHUA_pNF-kB(p65).tif]

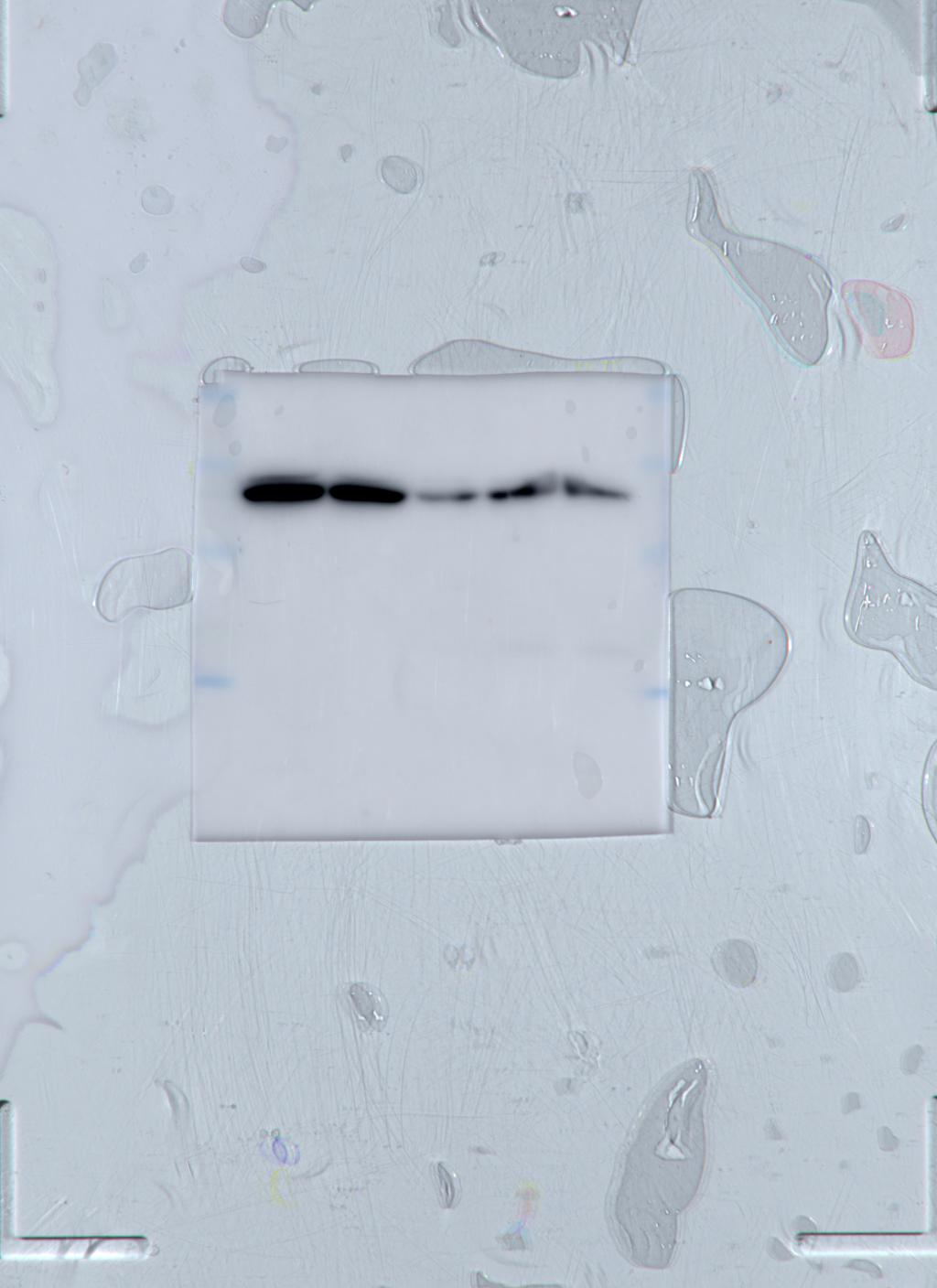

Supplement: Supplementary file 1 [file cancers-18-00198-s001.zip › Figure S1 and S2 Kurosu Original Images for Blots or Gels or Microscopy/Figure10 SPAC-1-L_ERK (the 3 bands on the left).tif]

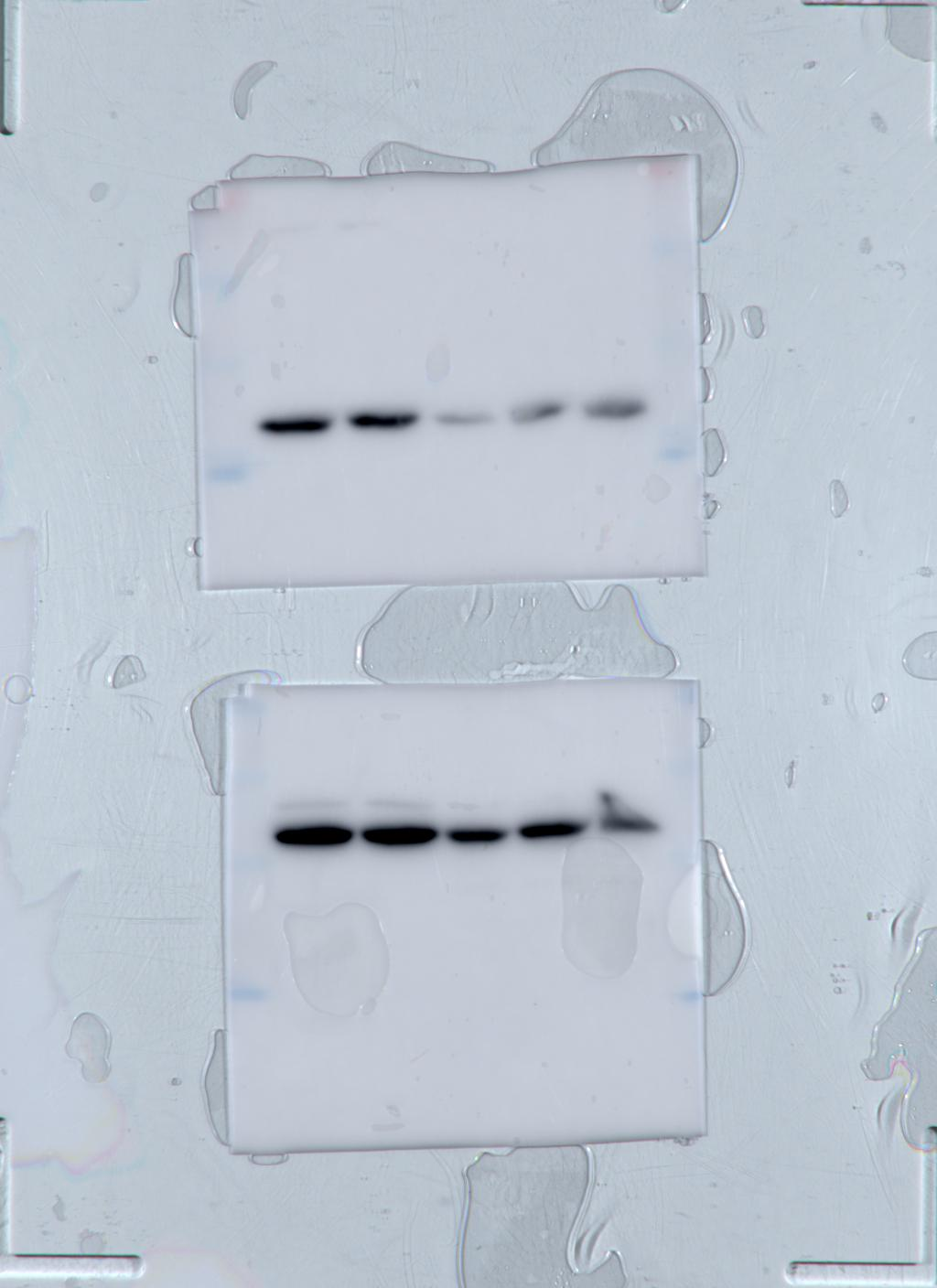

Supplement: Supplementary file 1 [file cancers-18-00198-s001.zip › Figure S1 and S2 Kurosu Original Images for Blots or Gels or Microscopy/Figure10 SPAC-1-L_GAPDH (the 3 bands on the left)(top_same membrane as L1CAM&NF-kB)(bottom_same membrane as ERK).tif]

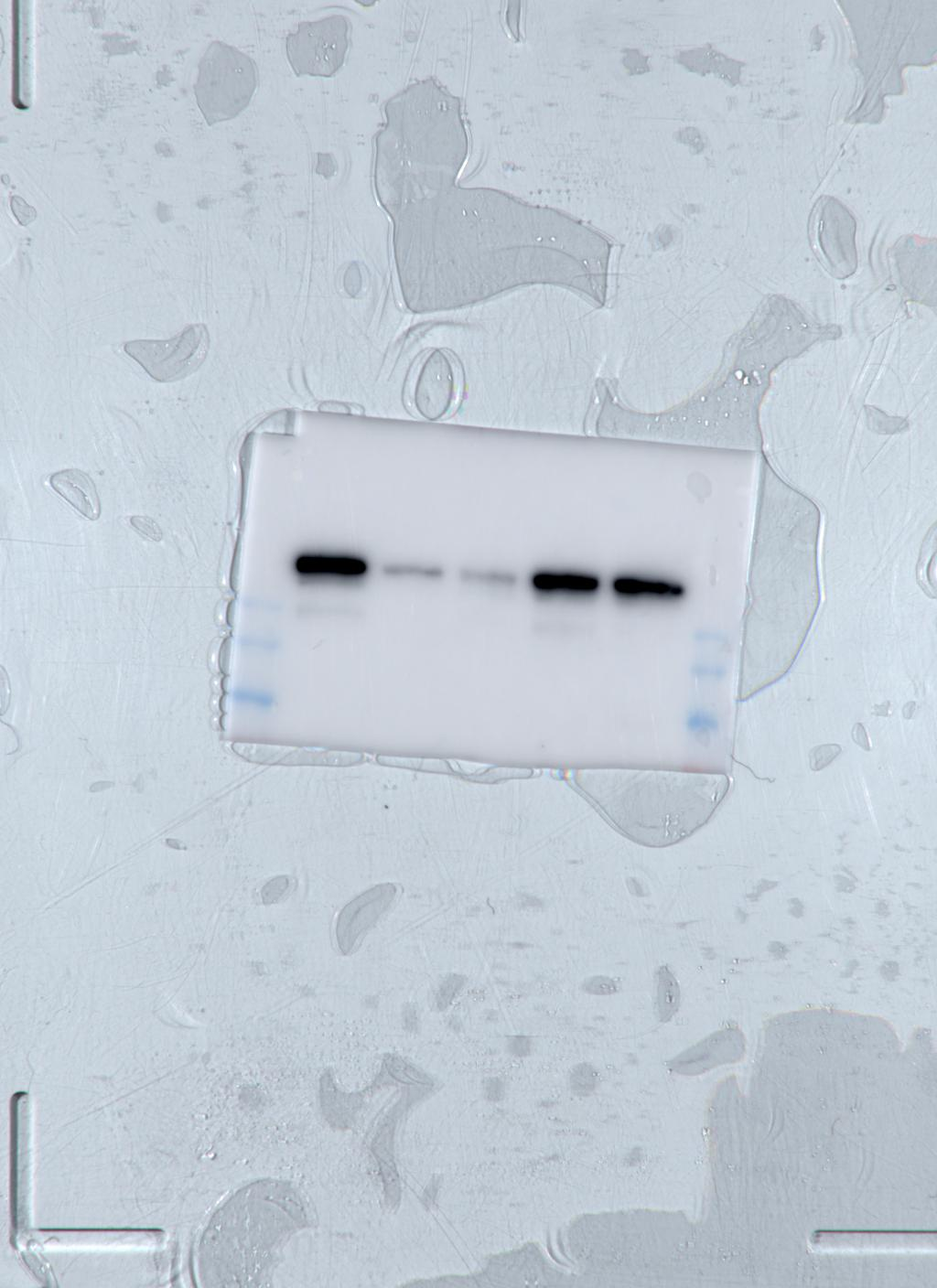

Supplement: Supplementary file 1 [file cancers-18-00198-s001.zip › Figure S1 and S2 Kurosu Original Images for Blots or Gels or Microscopy/Figure10 SPAC-1-L_L1CAM (the 3 bands on the left).tif]

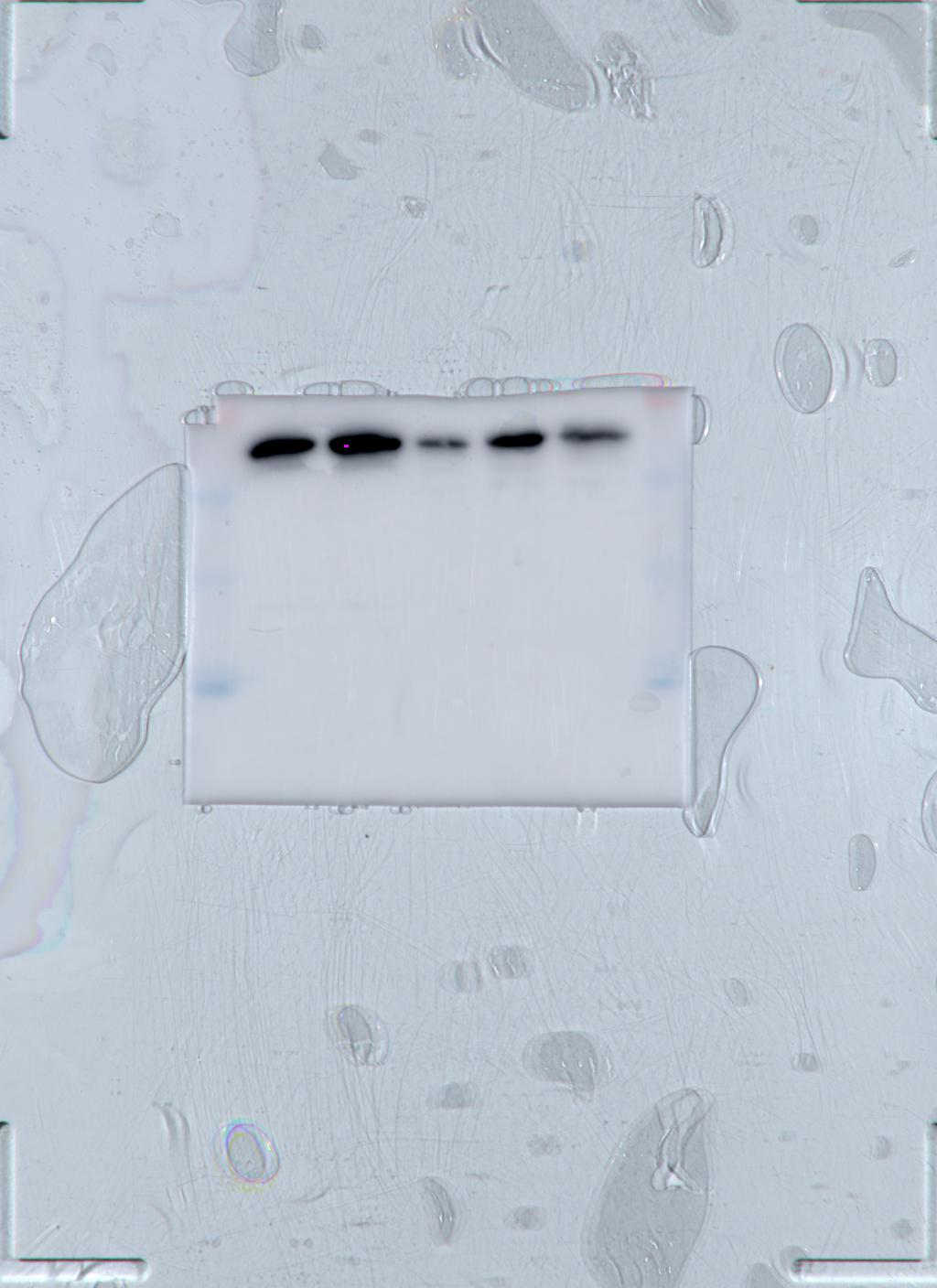

Supplement: Supplementary file 1 [file cancers-18-00198-s001.zip › Figure S1 and S2 Kurosu Original Images for Blots or Gels or Microscopy/Figure10 SPAC-1-L_NF-kB (the 3 bands on the left).tif]

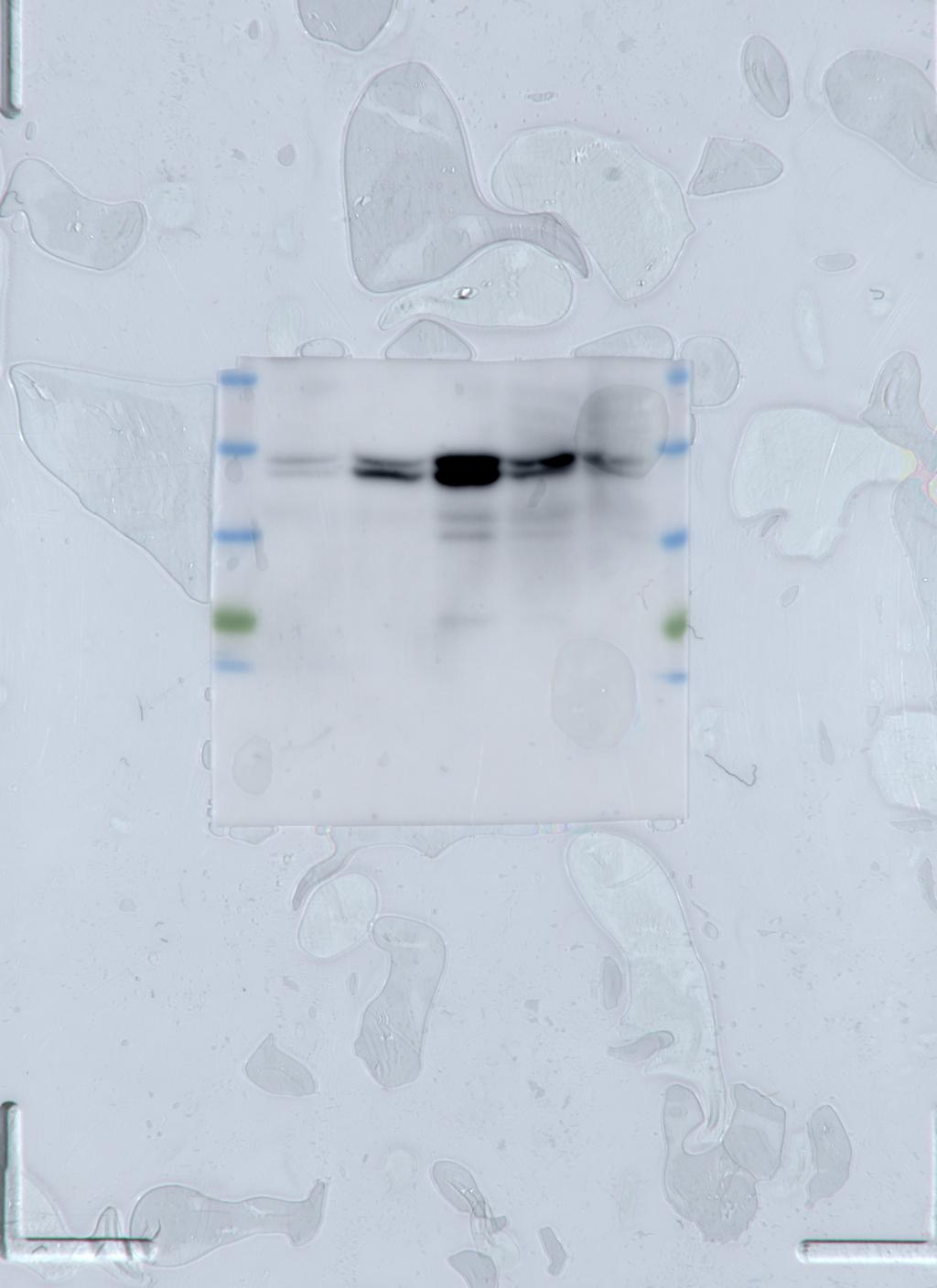

Supplement: Supplementary file 1 [file cancers-18-00198-s001.zip › Figure S1 and S2 Kurosu Original Images for Blots or Gels or Microscopy/Figure10 SPAC-1-L_pERK1_2 (the 3 bands on the left).tif]

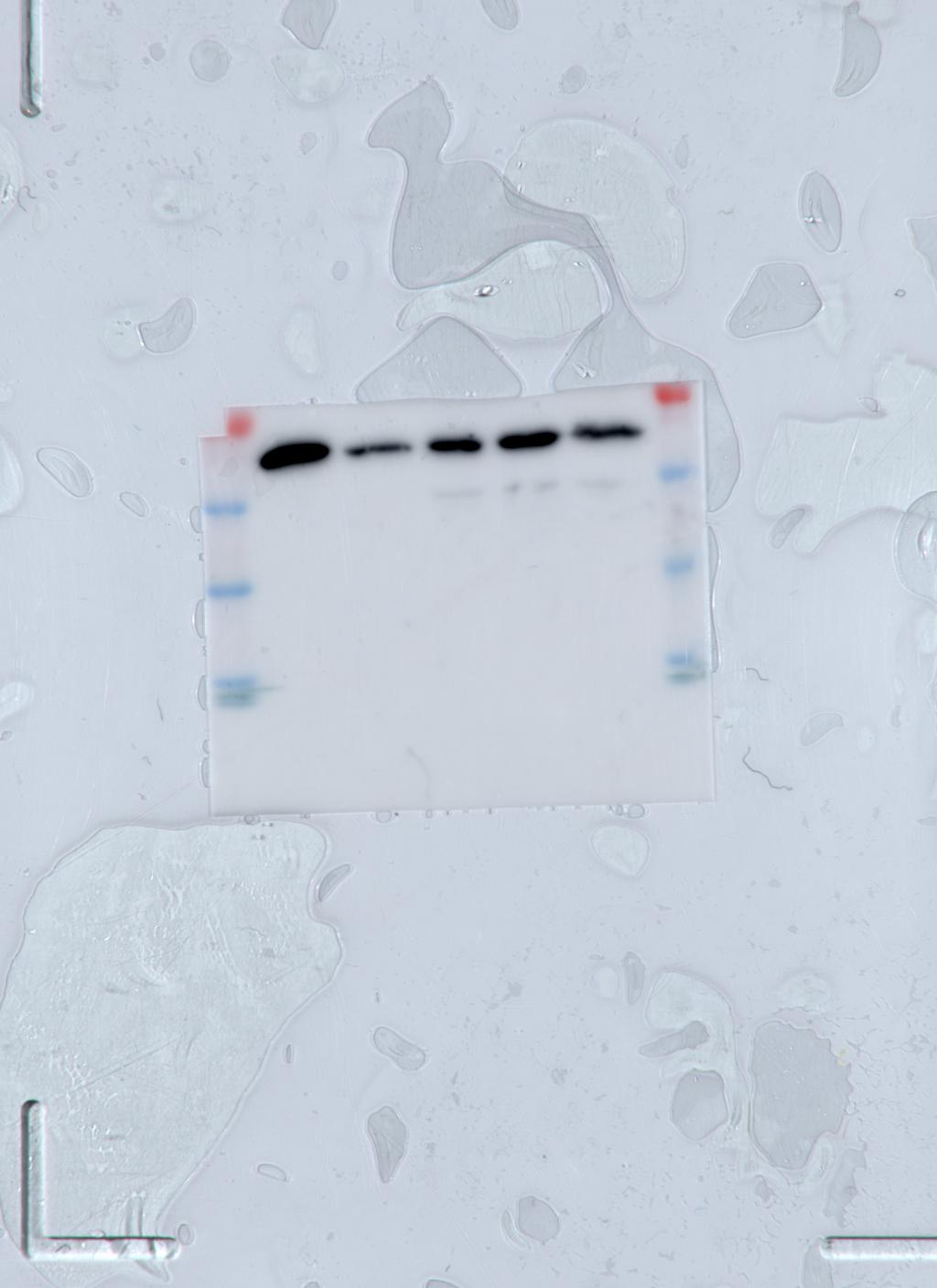

Supplement: Supplementary file 1 [file cancers-18-00198-s001.zip › Figure S1 and S2 Kurosu Original Images for Blots or Gels or Microscopy/Figure10 SPAC-1-L_pNF-kB (the 3 bands on the left).tif]

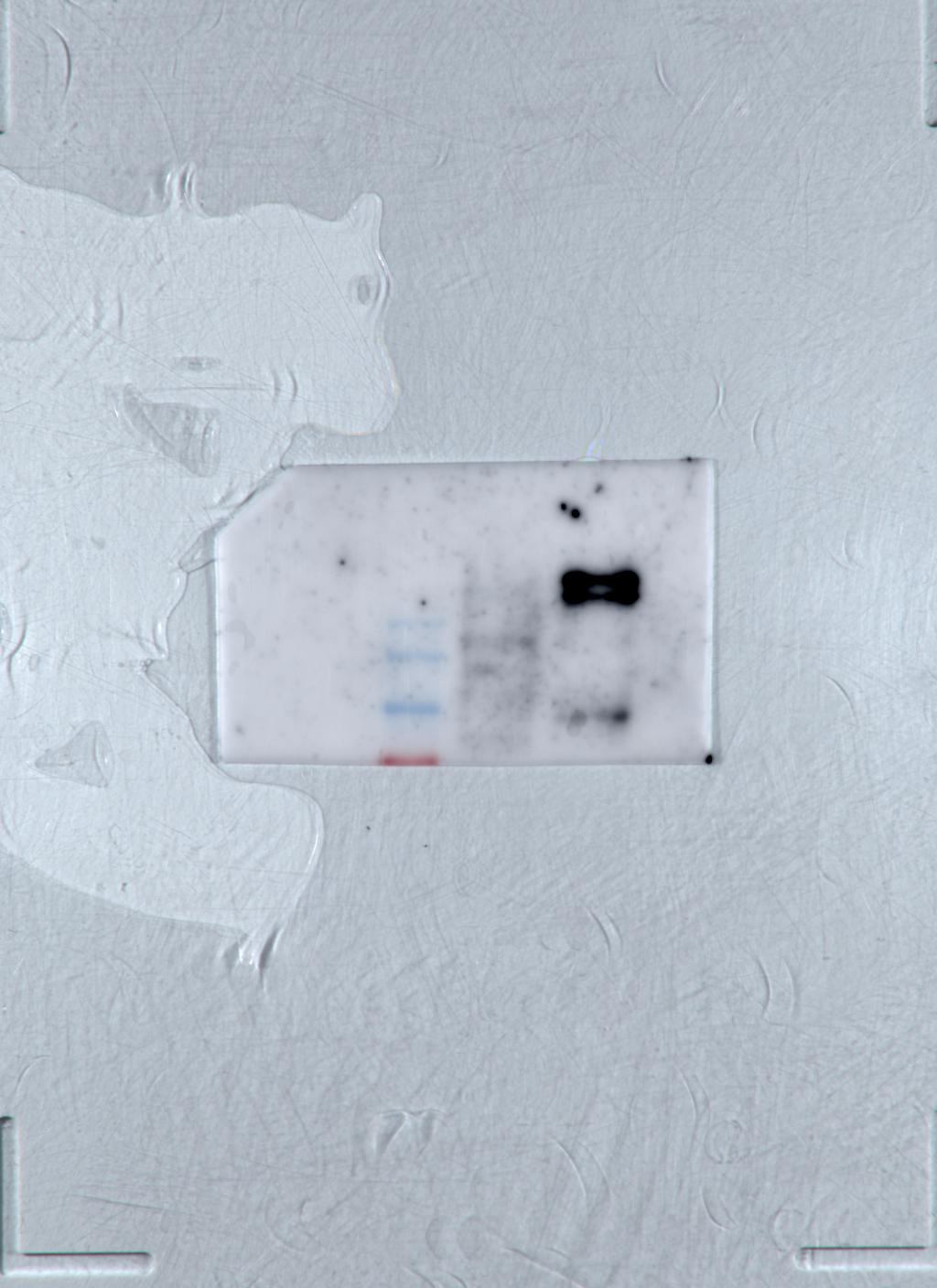

Supplement: Supplementary file 1 [file cancers-18-00198-s001.zip › Figure S1 and S2 Kurosu Original Images for Blots or Gels or Microscopy/Figure14 HHUA_Flag(L1CAM).tif]

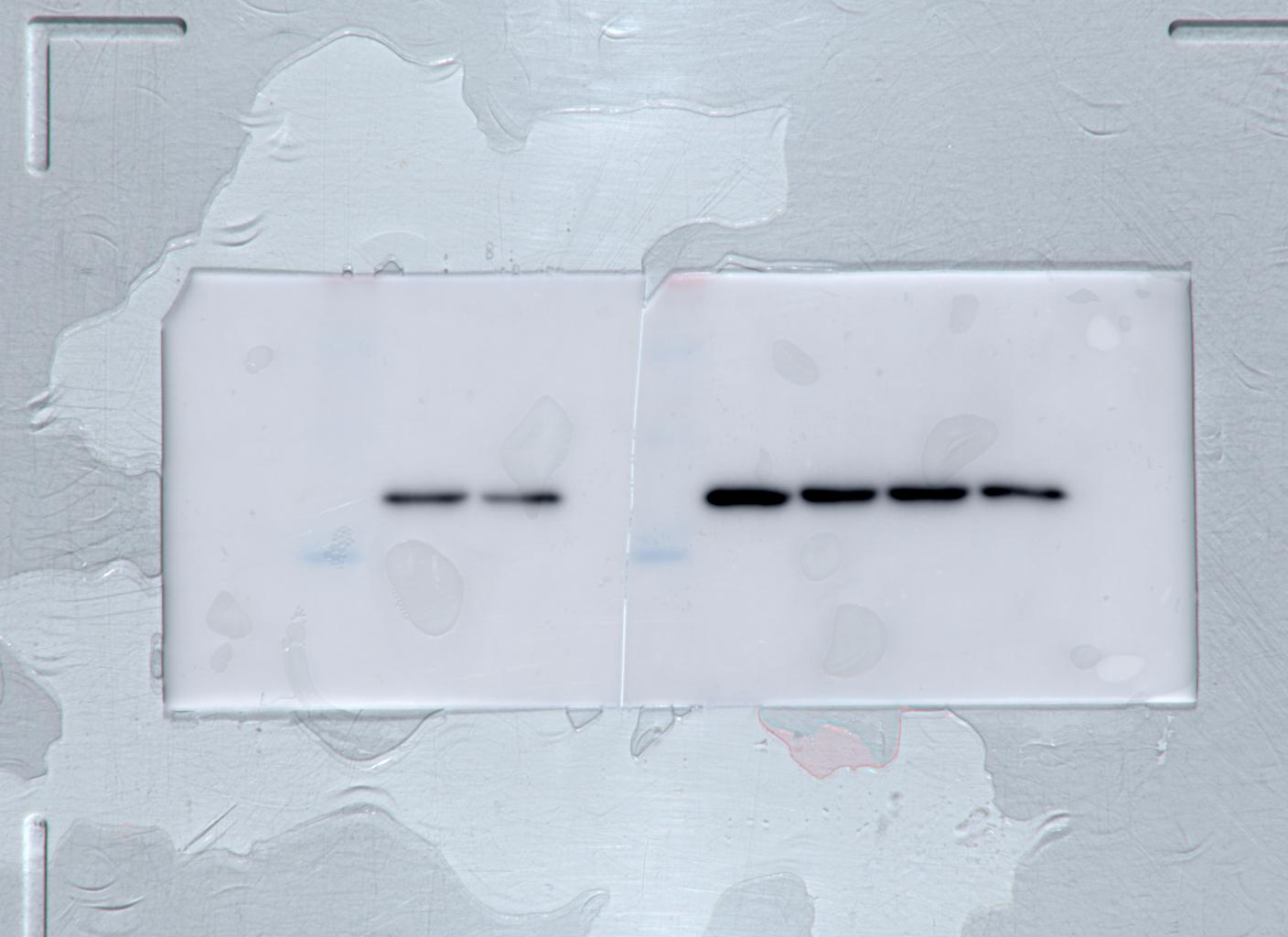

Supplement: Supplementary file 1 [file cancers-18-00198-s001.zip › Figure S1 and S2 Kurosu Original Images for Blots or Gels or Microscopy/Figure14 HHUA_GAPDH(left membrane).tif]

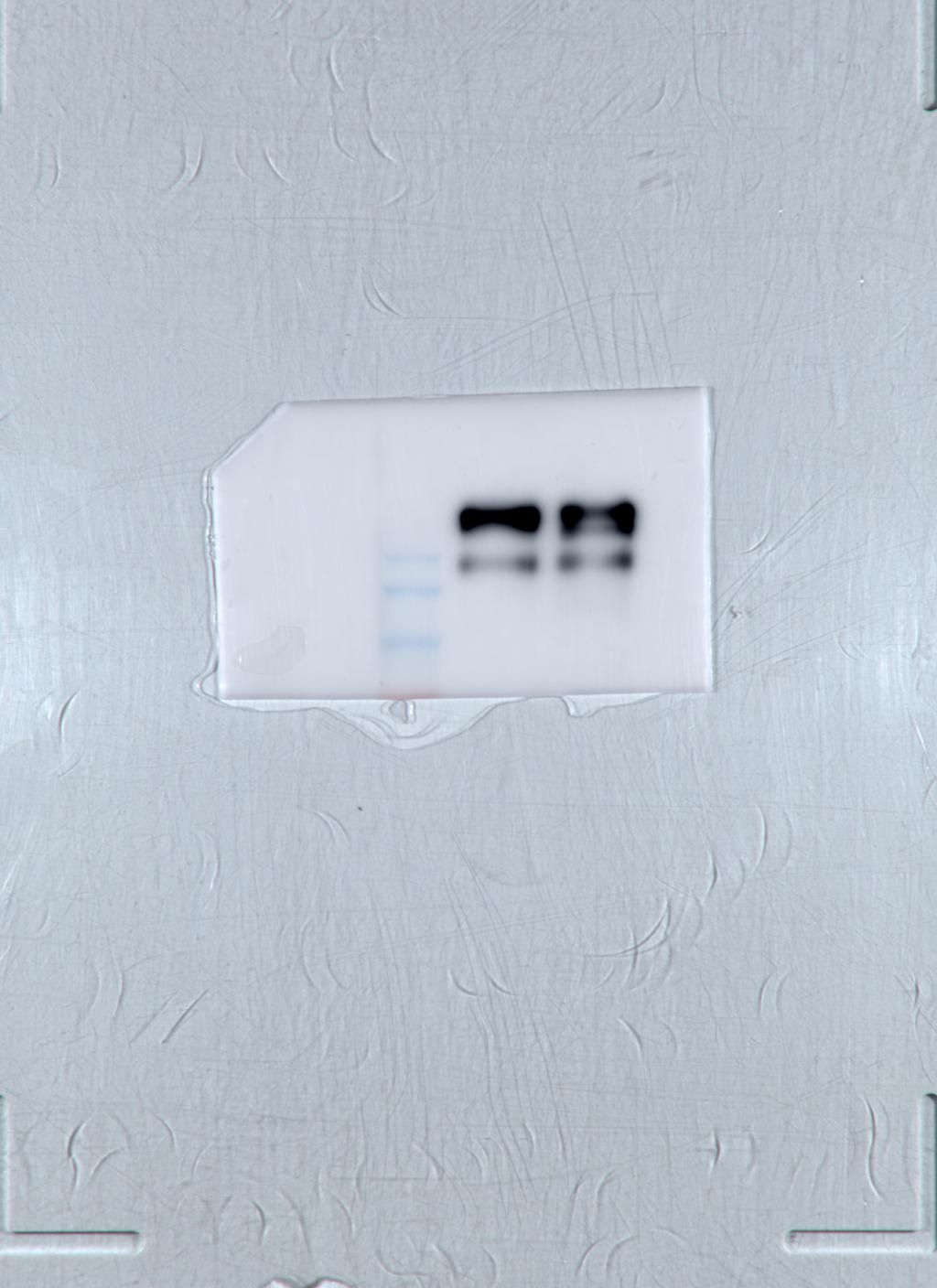

Supplement: Supplementary file 1 [file cancers-18-00198-s001.zip › Figure S1 and S2 Kurosu Original Images for Blots or Gels or Microscopy/Figure14 HHUA_L1CAM.tif]

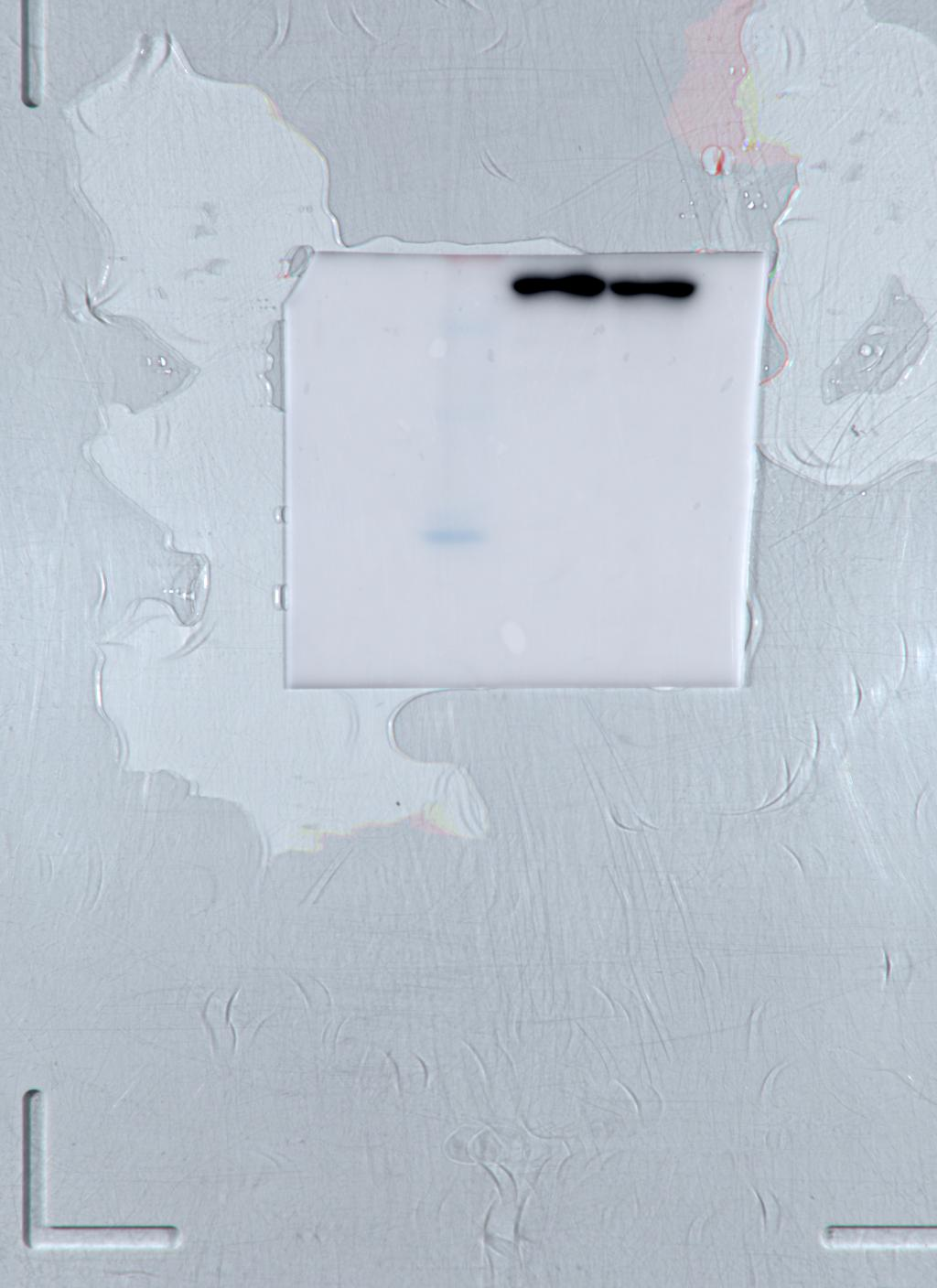

Supplement: Supplementary file 1 [file cancers-18-00198-s001.zip › Figure S1 and S2 Kurosu Original Images for Blots or Gels or Microscopy/Figure14 HHUA_NF-kB(p65).tif]

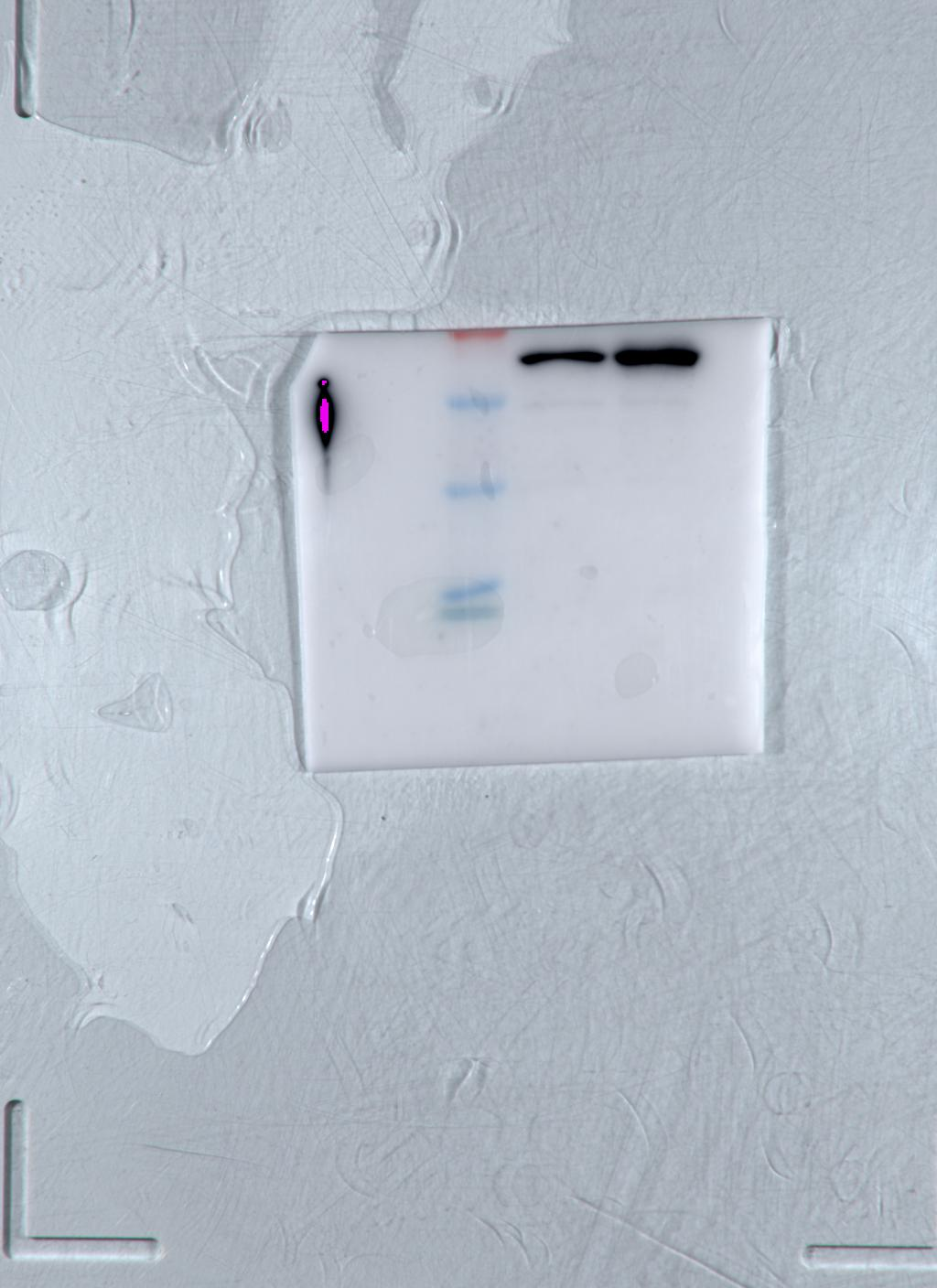

Supplement: Supplementary file 1 [file cancers-18-00198-s001.zip › Figure S1 and S2 Kurosu Original Images for Blots or Gels or Microscopy/Figure14 HHUA_pNF-kB(p65).tif]

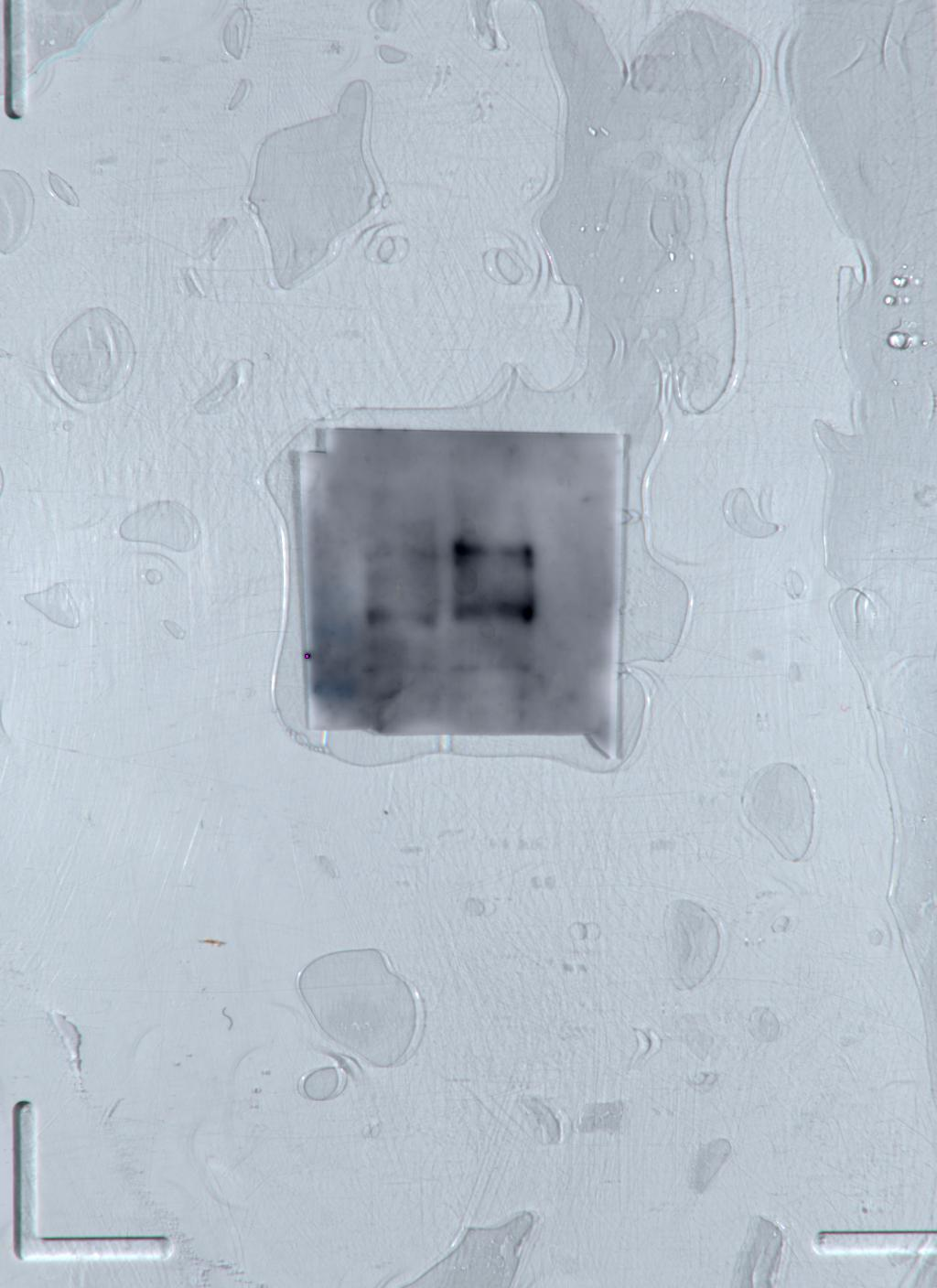

Supplement: Supplementary file 1 [file cancers-18-00198-s001.zip › Figure S1 and S2 Kurosu Original Images for Blots or Gels or Microscopy/Figure14 Ishikawa_Flag(L1CAM).tif]

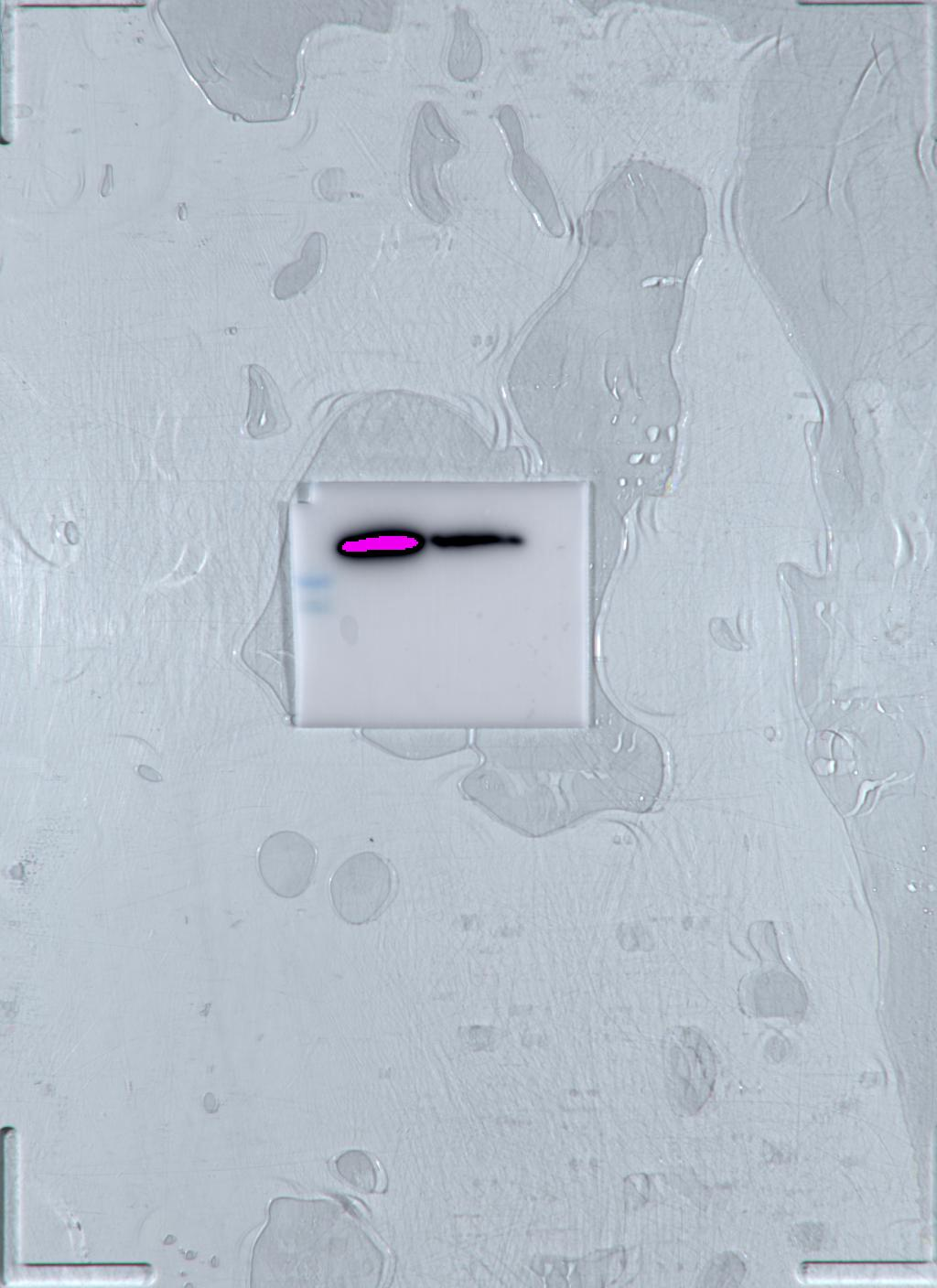

Supplement: Supplementary file 1 [file cancers-18-00198-s001.zip › Figure S1 and S2 Kurosu Original Images for Blots or Gels or Microscopy/Figure14 Ishikawa_GAPDH.tif]

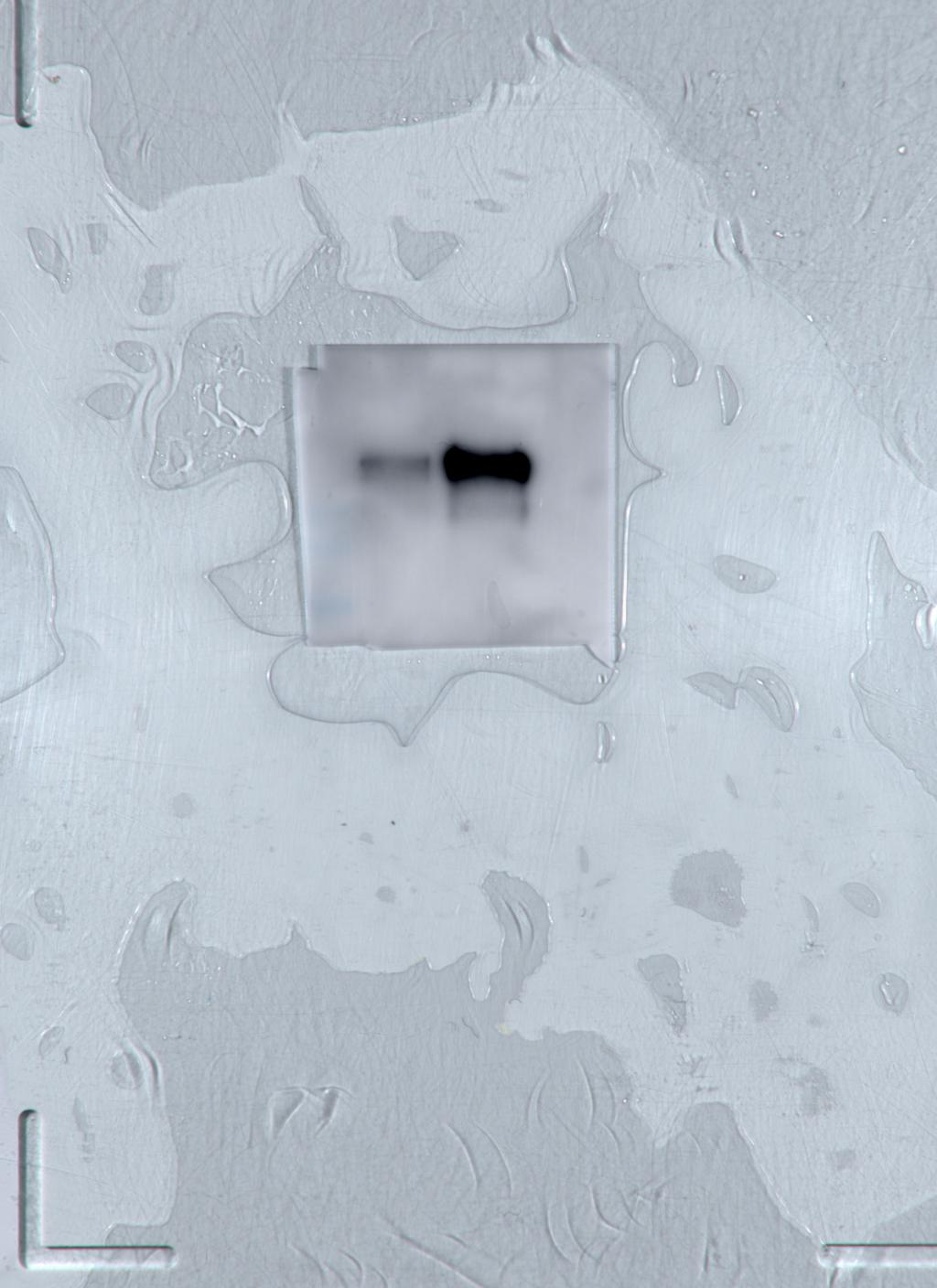

Supplement: Supplementary file 1 [file cancers-18-00198-s001.zip › Figure S1 and S2 Kurosu Original Images for Blots or Gels or Microscopy/Figure14 Ishikawa_L1CAM.tif]

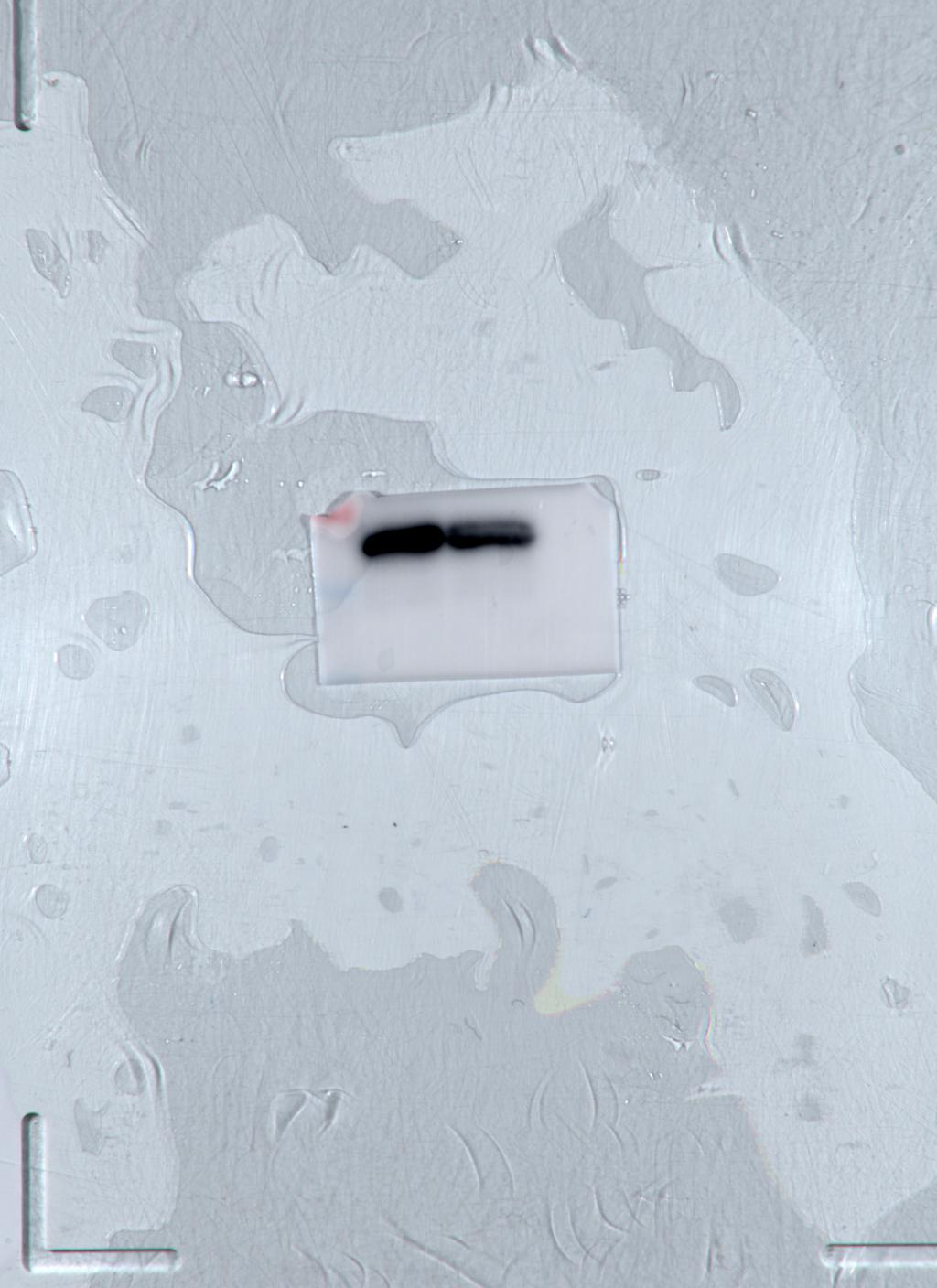

Supplement: Supplementary file 1 [file cancers-18-00198-s001.zip › Figure S1 and S2 Kurosu Original Images for Blots or Gels or Microscopy/Figure14 Ishikawa_NF-kB.tif]

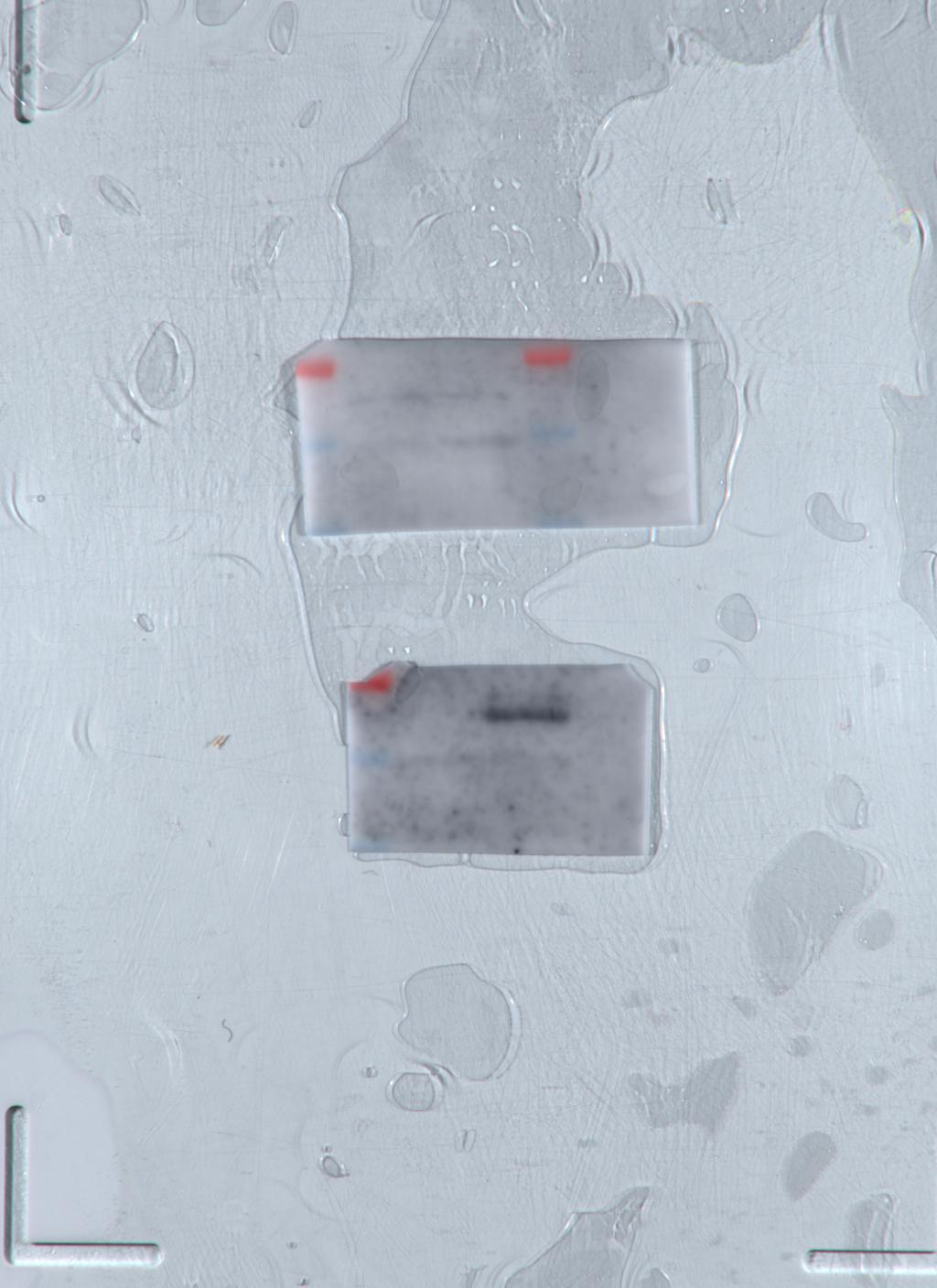

Supplement: Supplementary file 1 [file cancers-18-00198-s001.zip › Figure S1 and S2 Kurosu Original Images for Blots or Gels or Microscopy/Figure14 Ishikawa_pNF-kB (lower membarne).tif]

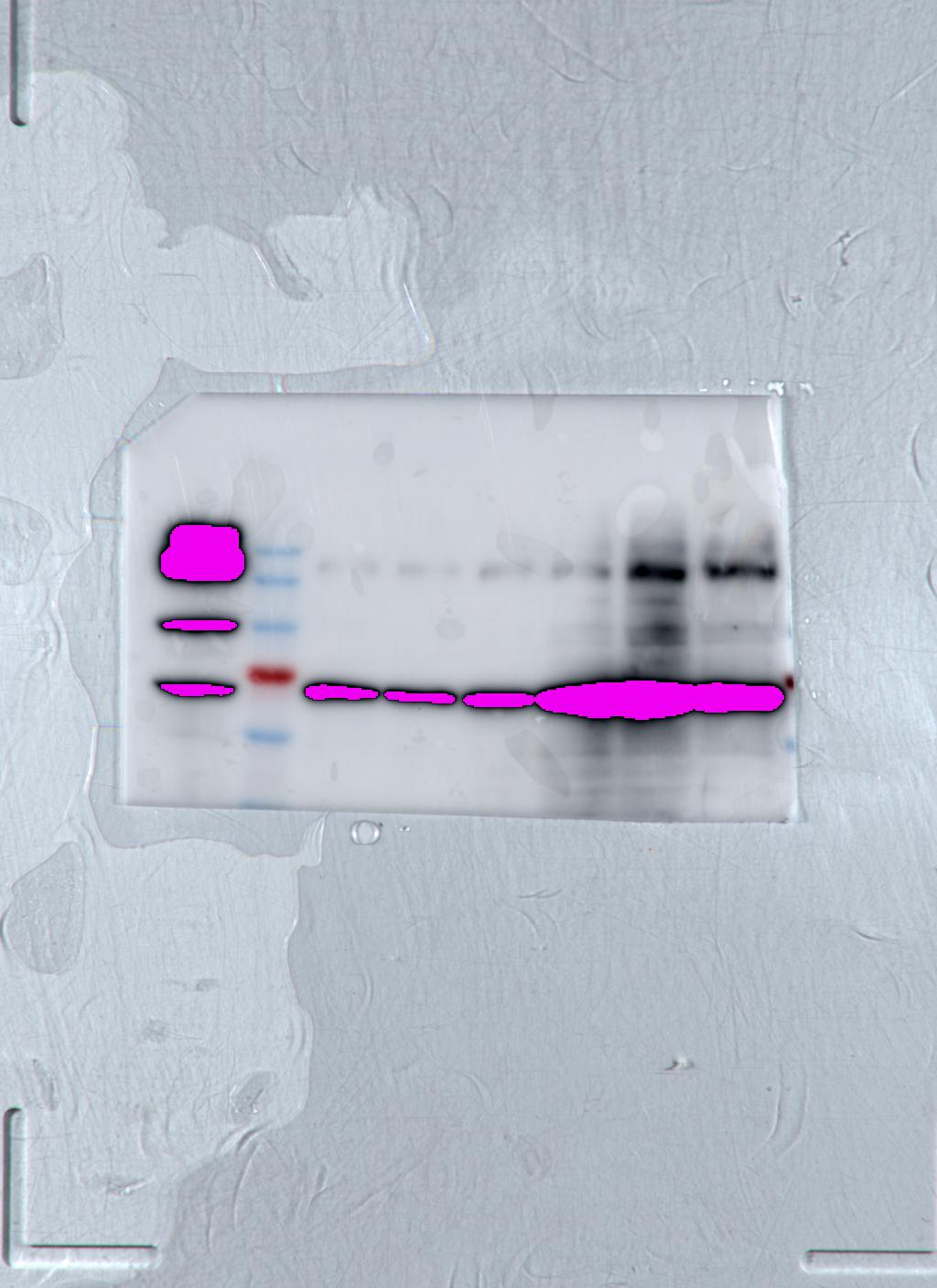

Supplement: Supplementary file 1 [file cancers-18-00198-s001.zip › Figure S1 and S2 Kurosu Original Images for Blots or Gels or Microscopy/Figure15 HHUA_Flag(IKKb(EE))(the 6bands on the right).tif]

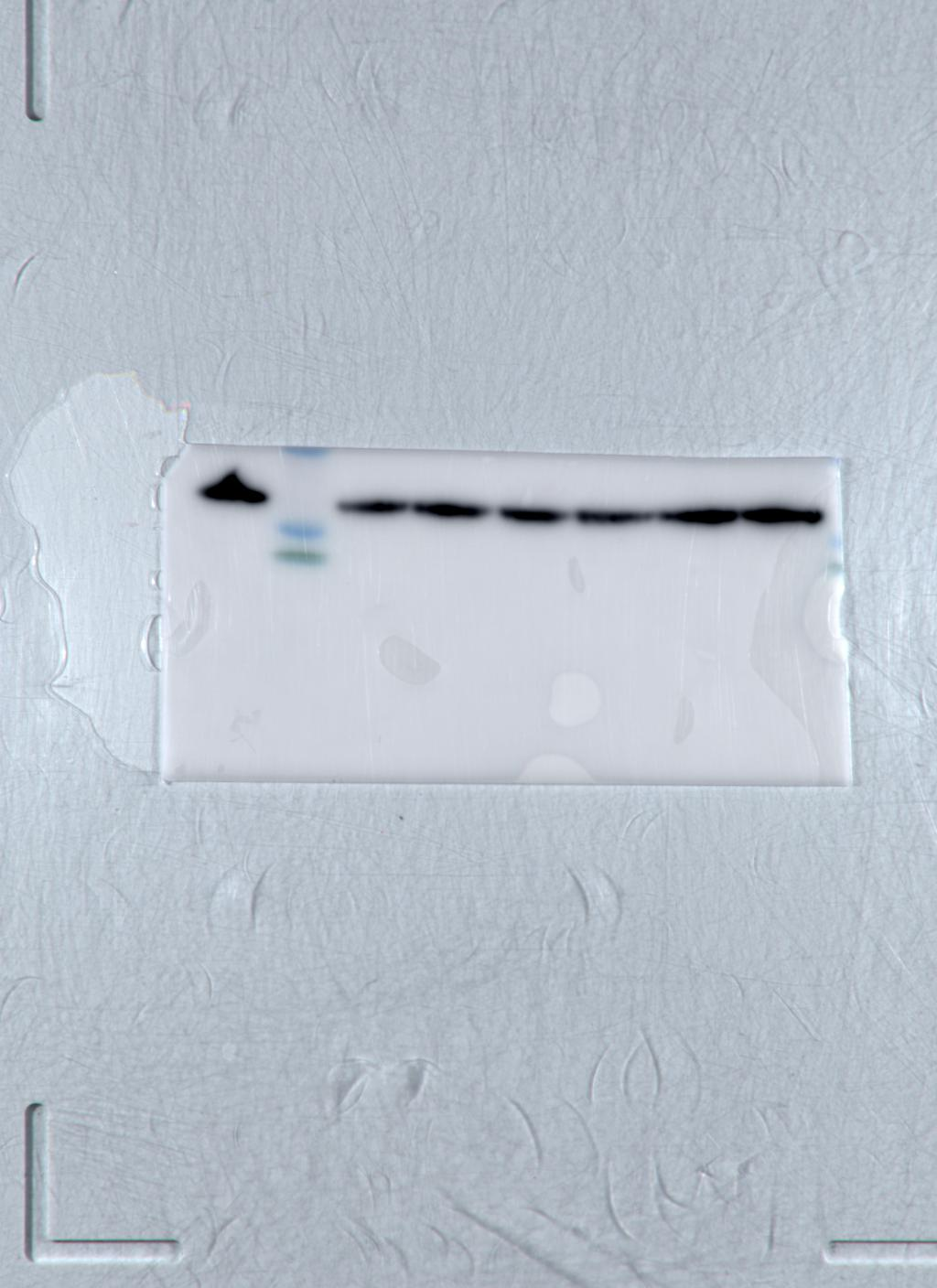

Supplement: Supplementary file 1 [file cancers-18-00198-s001.zip › Figure S1 and S2 Kurosu Original Images for Blots or Gels or Microscopy/Figure15 HHUA_GAPDH(same membrane as Flag(IKKb(EE))(unused in Figure15).tif]

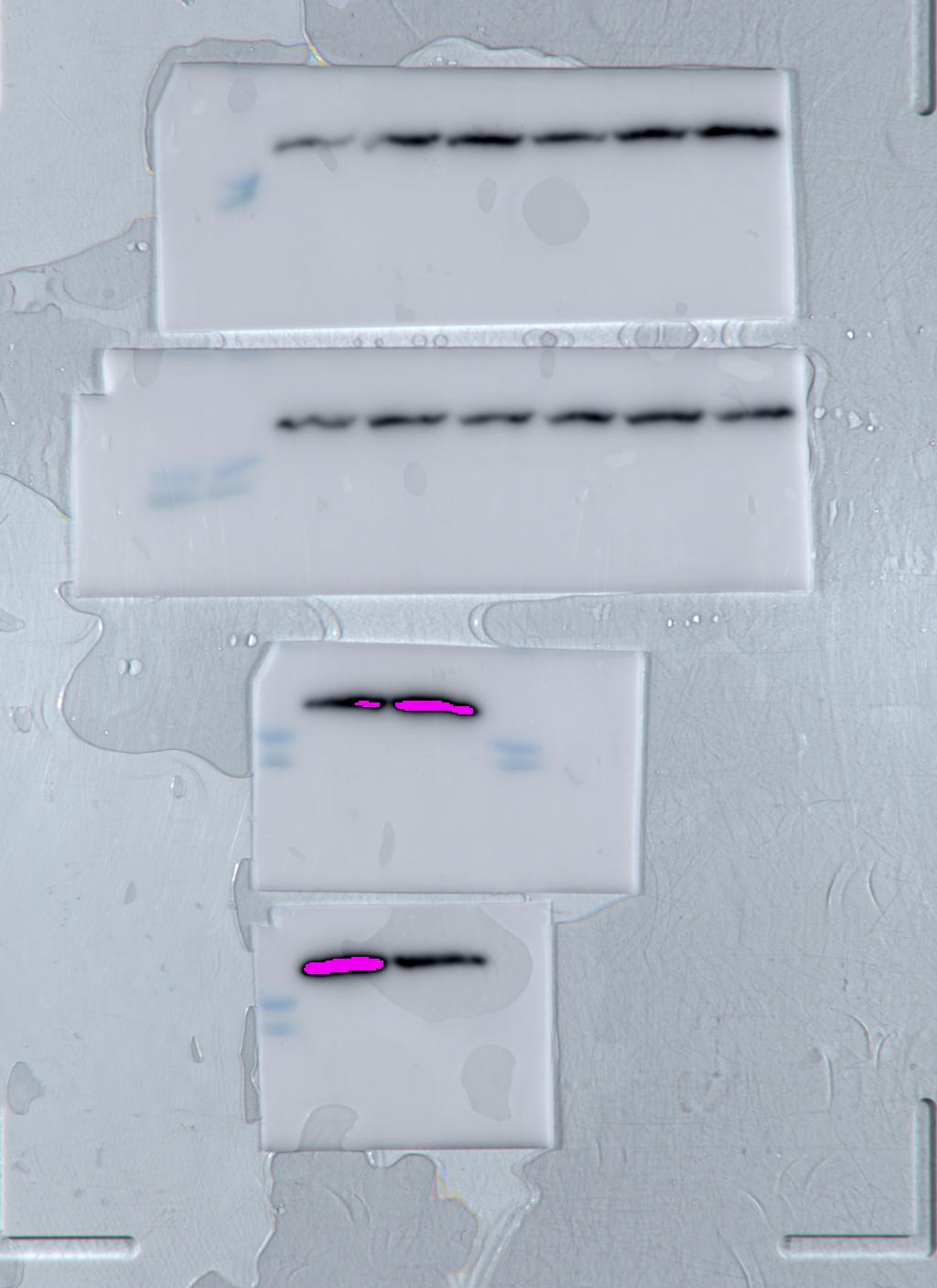

Supplement: Supplementary file 1 [file cancers-18-00198-s001.zip › Figure S1 and S2 Kurosu Original Images for Blots or Gels or Microscopy/Figure15 HHUA_GAPDH(upper membrane).tif]

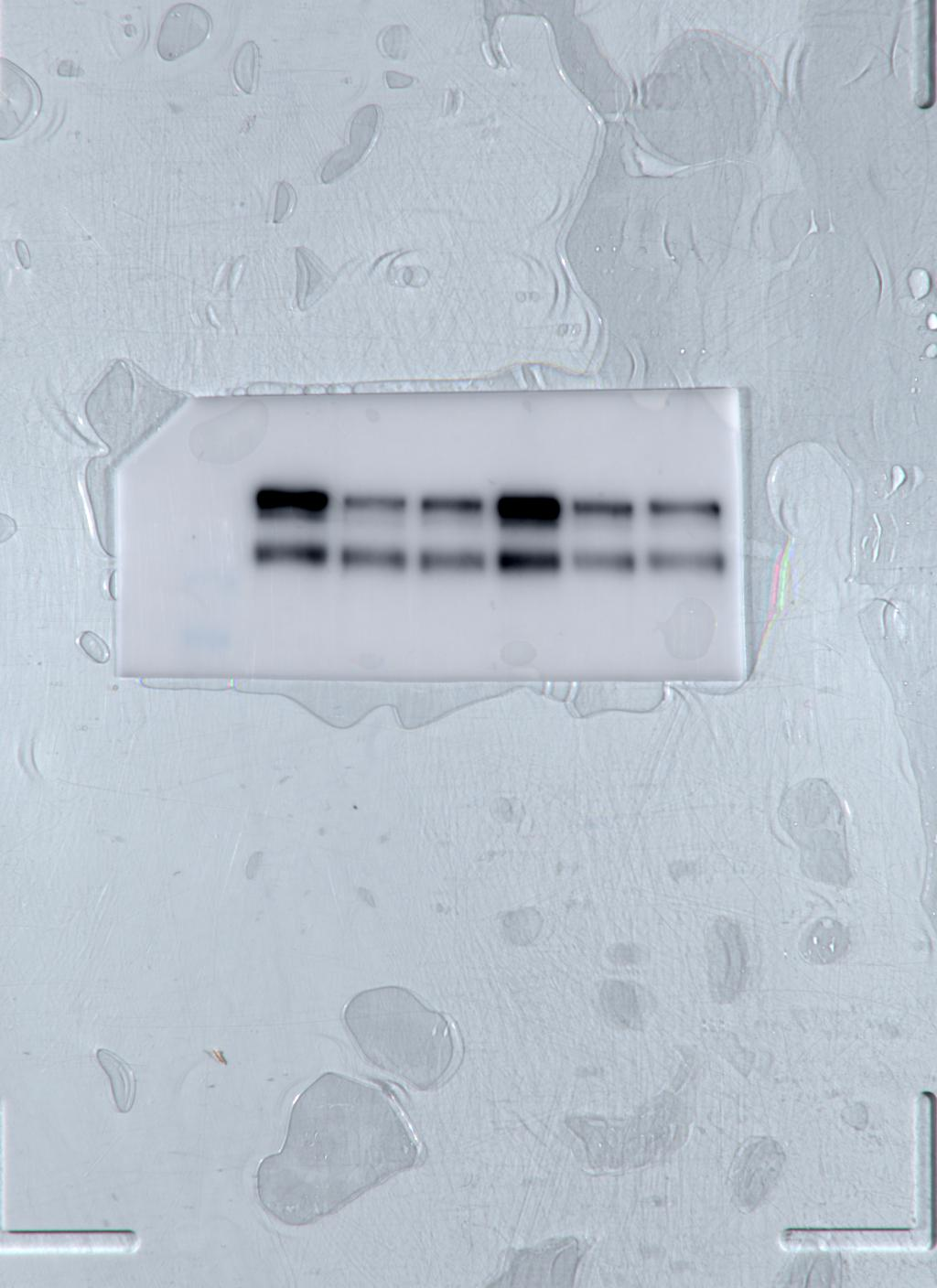

Supplement: Supplementary file 1 [file cancers-18-00198-s001.zip › Figure S1 and S2 Kurosu Original Images for Blots or Gels or Microscopy/Figure15 HHUA_L1CAM.tif]

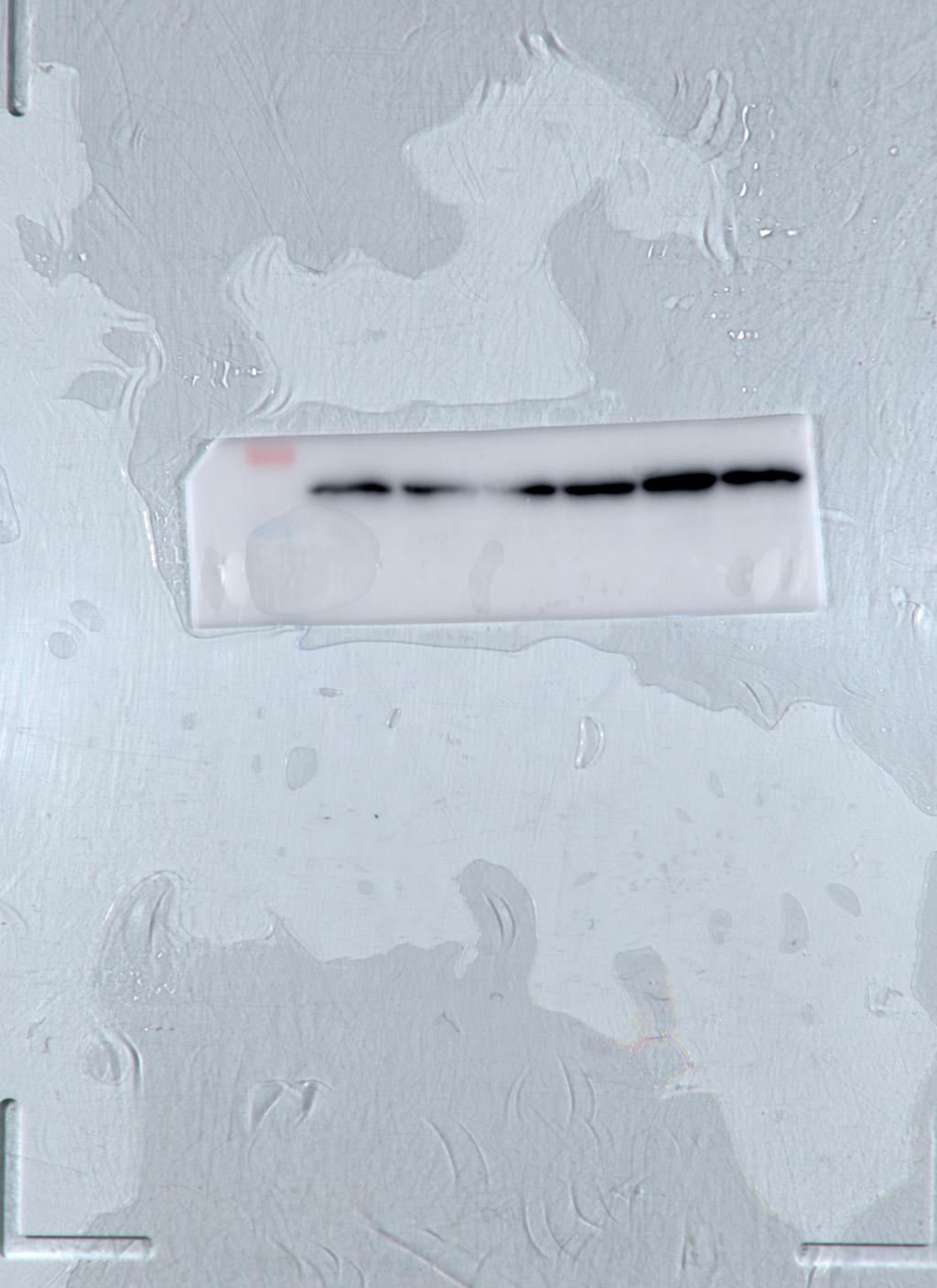

Supplement: Supplementary file 1 [file cancers-18-00198-s001.zip › Figure S1 and S2 Kurosu Original Images for Blots or Gels or Microscopy/Figure15 HHUA_NF-kB(p65).tif]

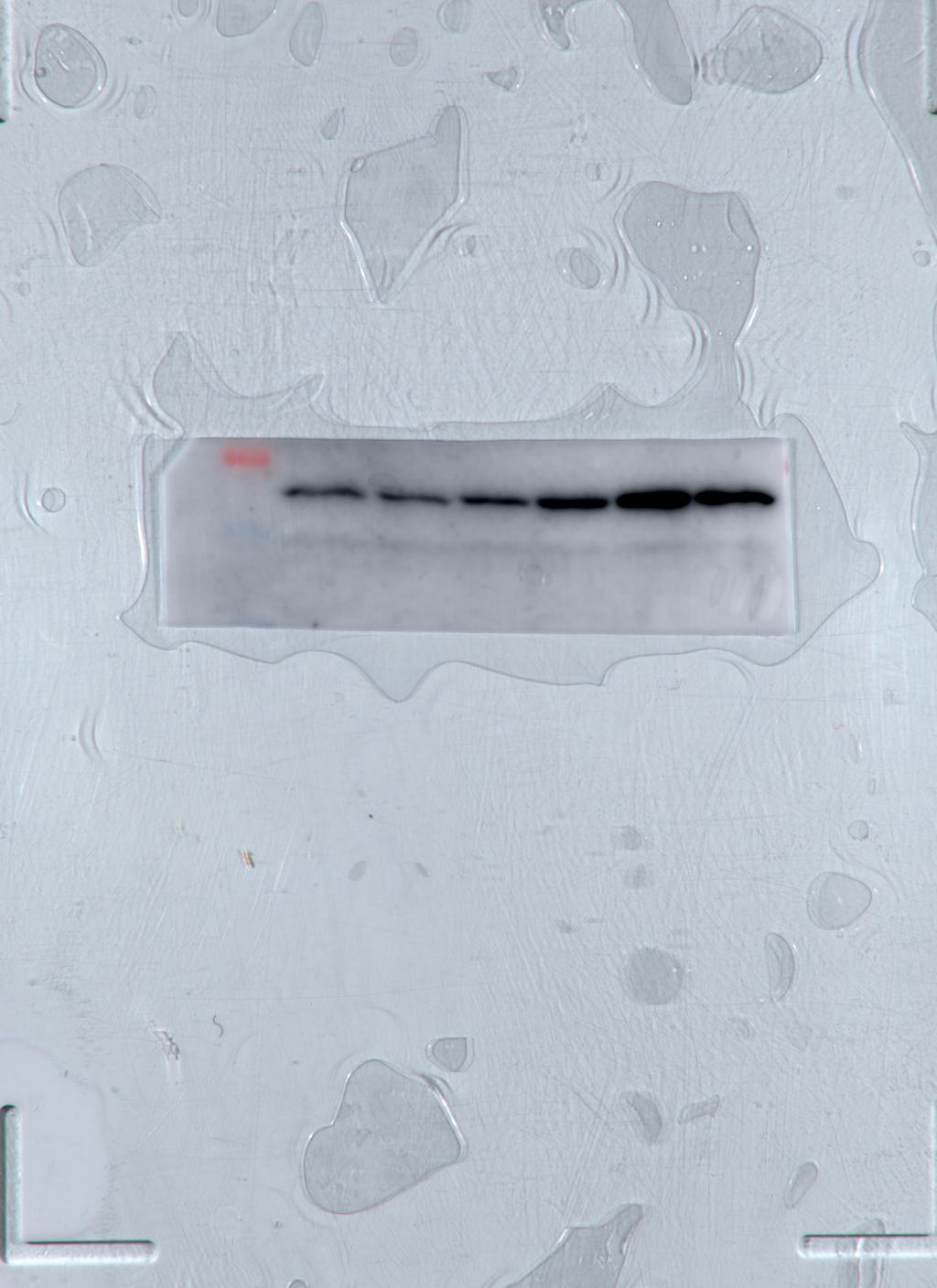

Supplement: Supplementary file 1 [file cancers-18-00198-s001.zip › Figure S1 and S2 Kurosu Original Images for Blots or Gels or Microscopy/Figure15 HHUA_pNF-kB(p65).tif]

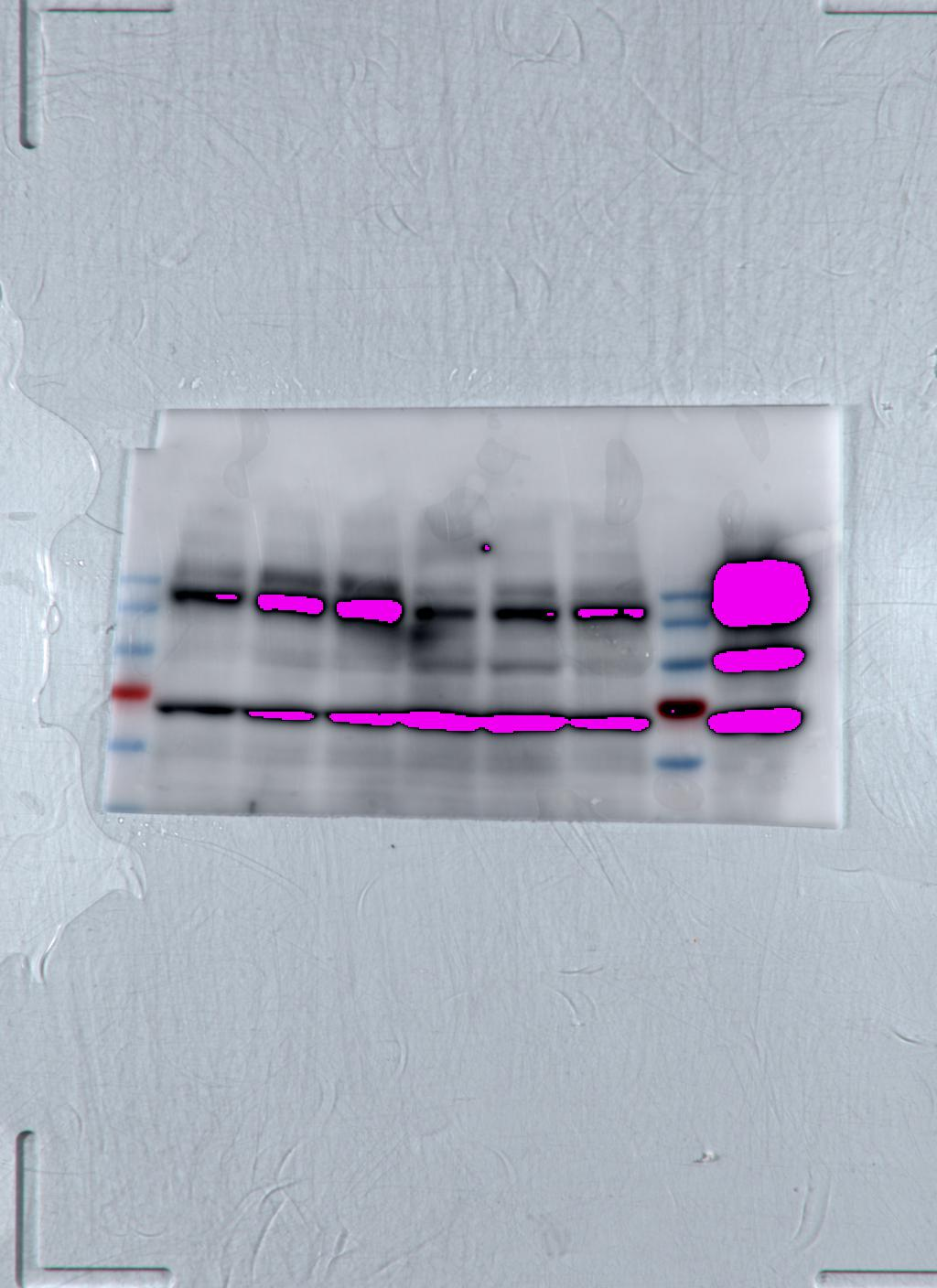

Supplement: Supplementary file 1 [file cancers-18-00198-s001.zip › Figure S1 and S2 Kurosu Original Images for Blots or Gels or Microscopy/Figure15 SPAC-1-L_Flag(IKKb(EE))(the 6bands on the left).tif]

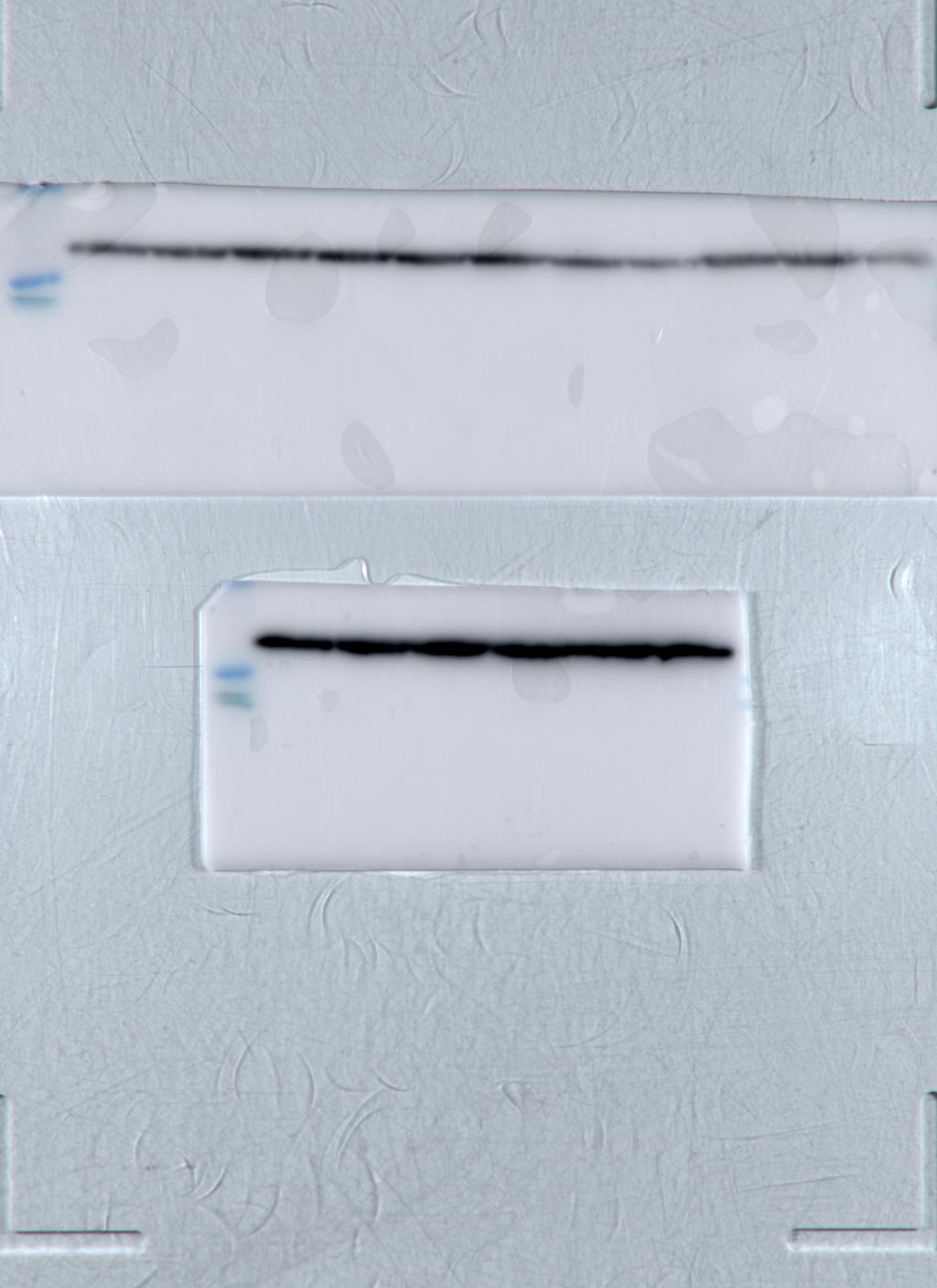

Supplement: Supplementary file 1 [file cancers-18-00198-s001.zip › Figure S1 and S2 Kurosu Original Images for Blots or Gels or Microscopy/Figure15 SPAC-1-L_GAPDH(bottom membrane).tif]

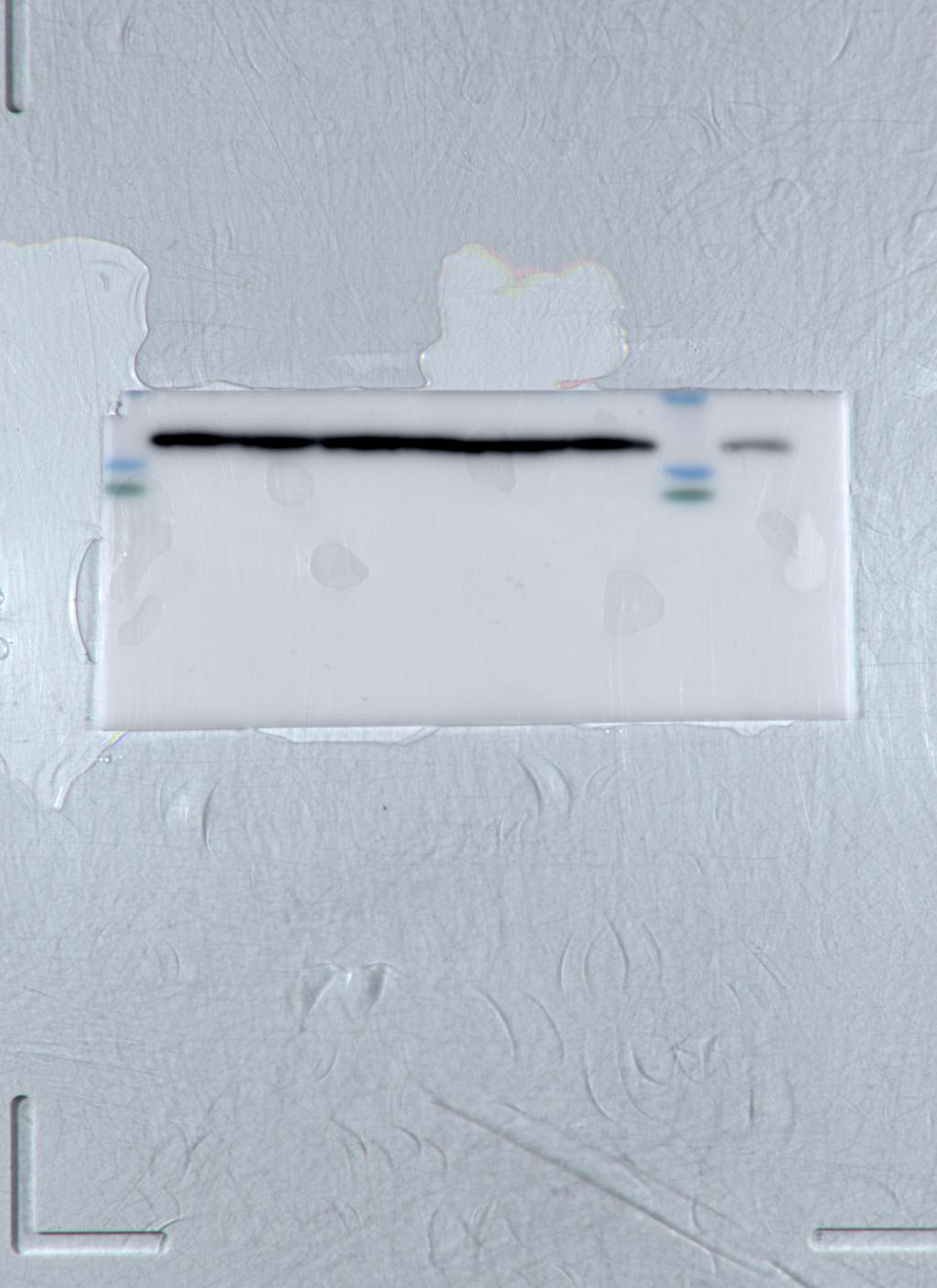

Supplement: Supplementary file 1 [file cancers-18-00198-s001.zip › Figure S1 and S2 Kurosu Original Images for Blots or Gels or Microscopy/Figure15 SPAC-1-L_GAPDH(same membrane as Flag(IKKb(EE))(unused in figure15).tif]

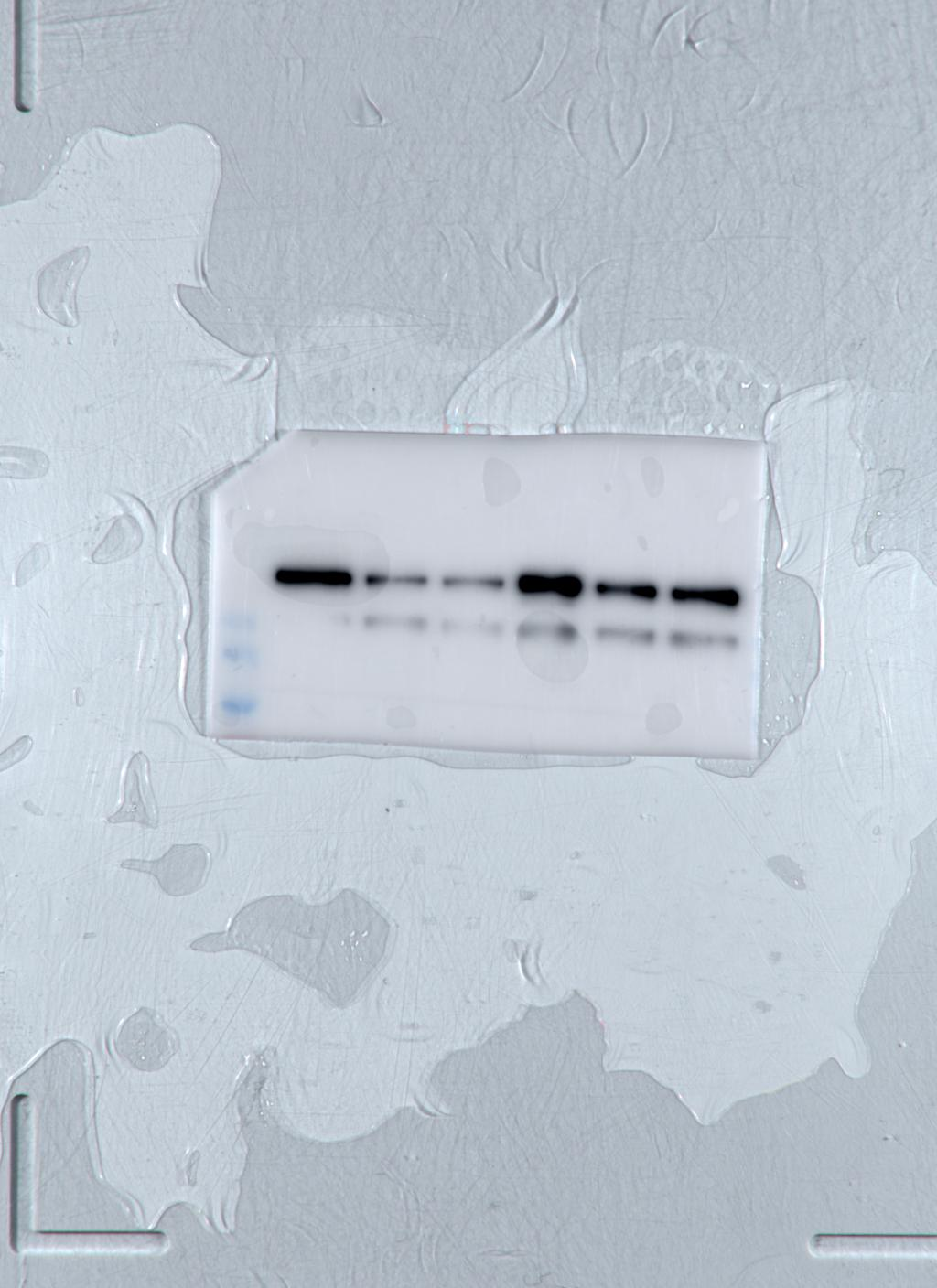

Supplement: Supplementary file 1 [file cancers-18-00198-s001.zip › Figure S1 and S2 Kurosu Original Images for Blots or Gels or Microscopy/Figure15 SPAC-1-L_L1CAM.tif]

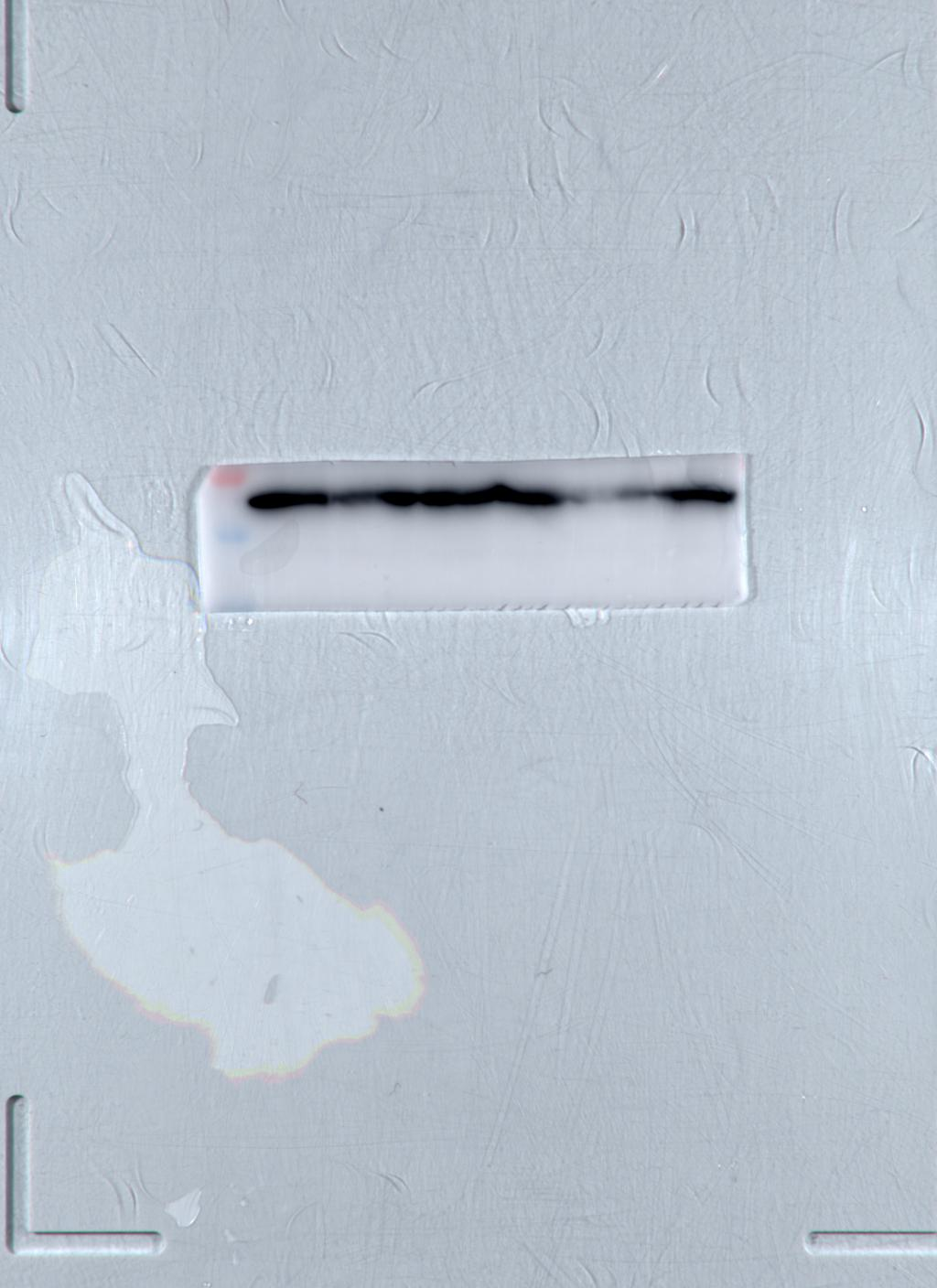

Supplement: Supplementary file 1 [file cancers-18-00198-s001.zip › Figure S1 and S2 Kurosu Original Images for Blots or Gels or Microscopy/Figure15 SPAC-1-L_NF-kB(p65).tif]

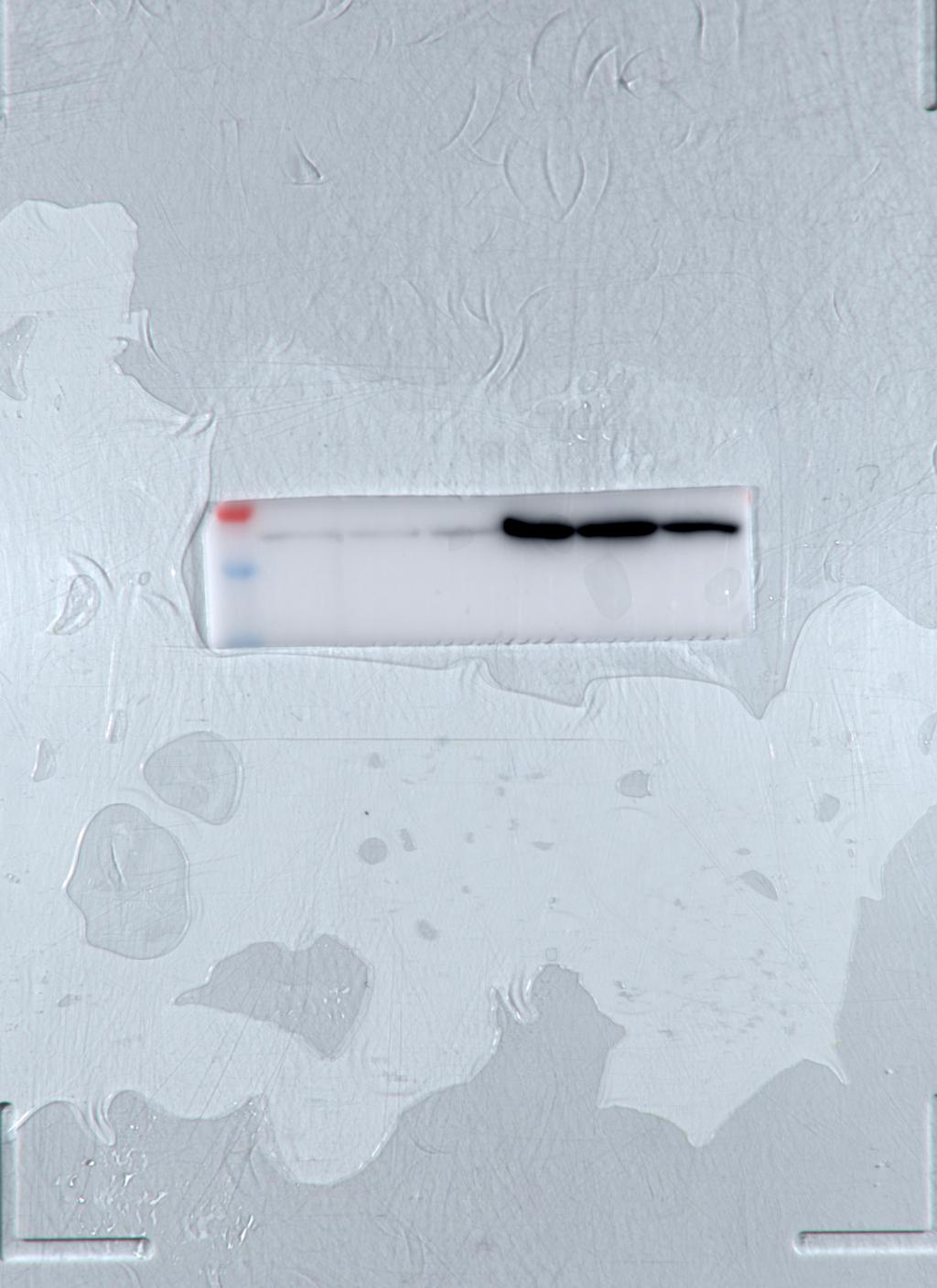

Supplement: Supplementary file 1 [file cancers-18-00198-s001.zip › Figure S1 and S2 Kurosu Original Images for Blots or Gels or Microscopy/Figure15 SPAC-1-L_pNF-kB(p65).tif]

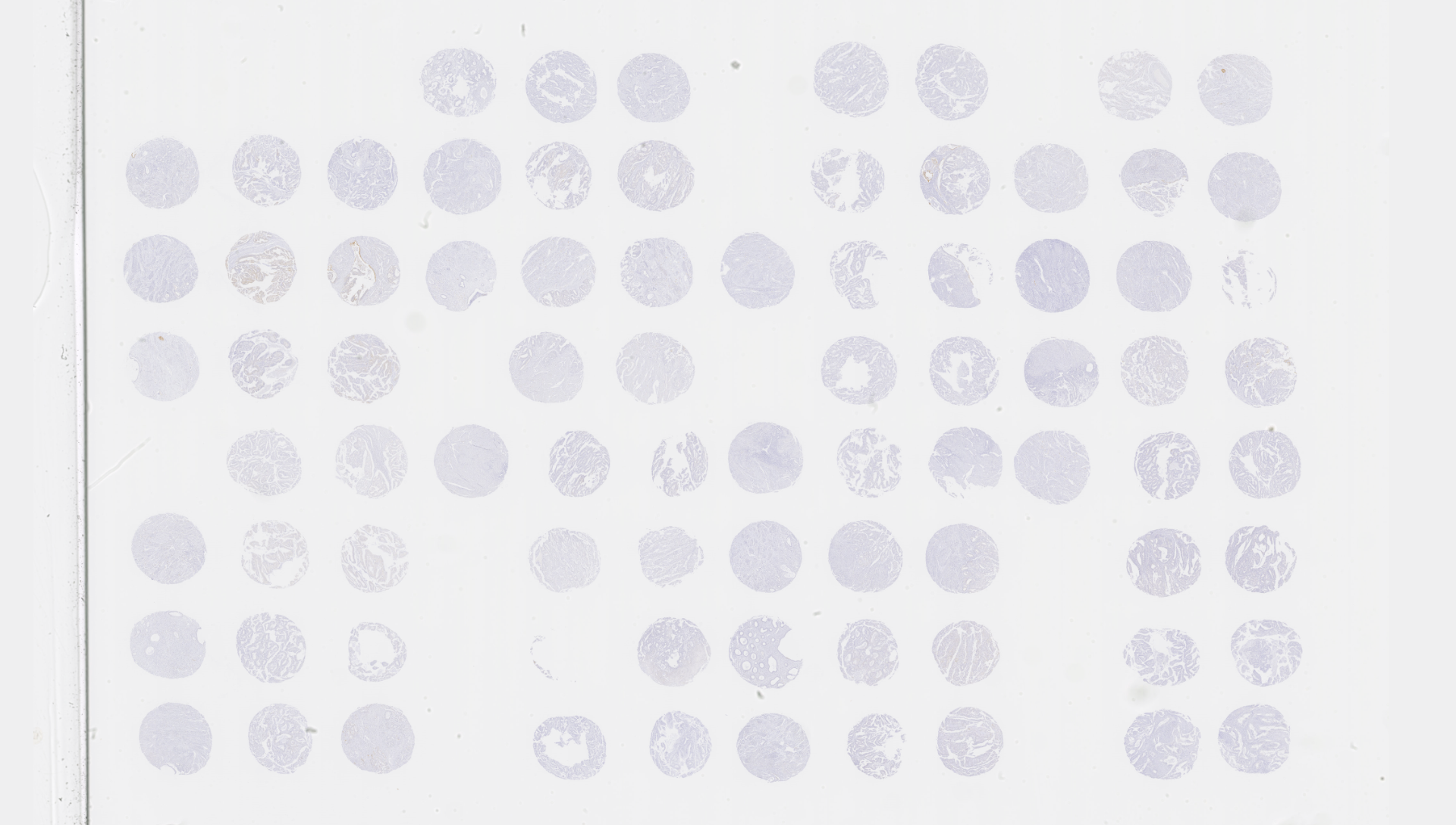

Supplement: Supplementary file 1 [file cancers-18-00198-s001.zip › Figure S1 and S2 Kurosu Original Images for Blots or Gels or Microscopy/Figure18_L1CAM1.tif]

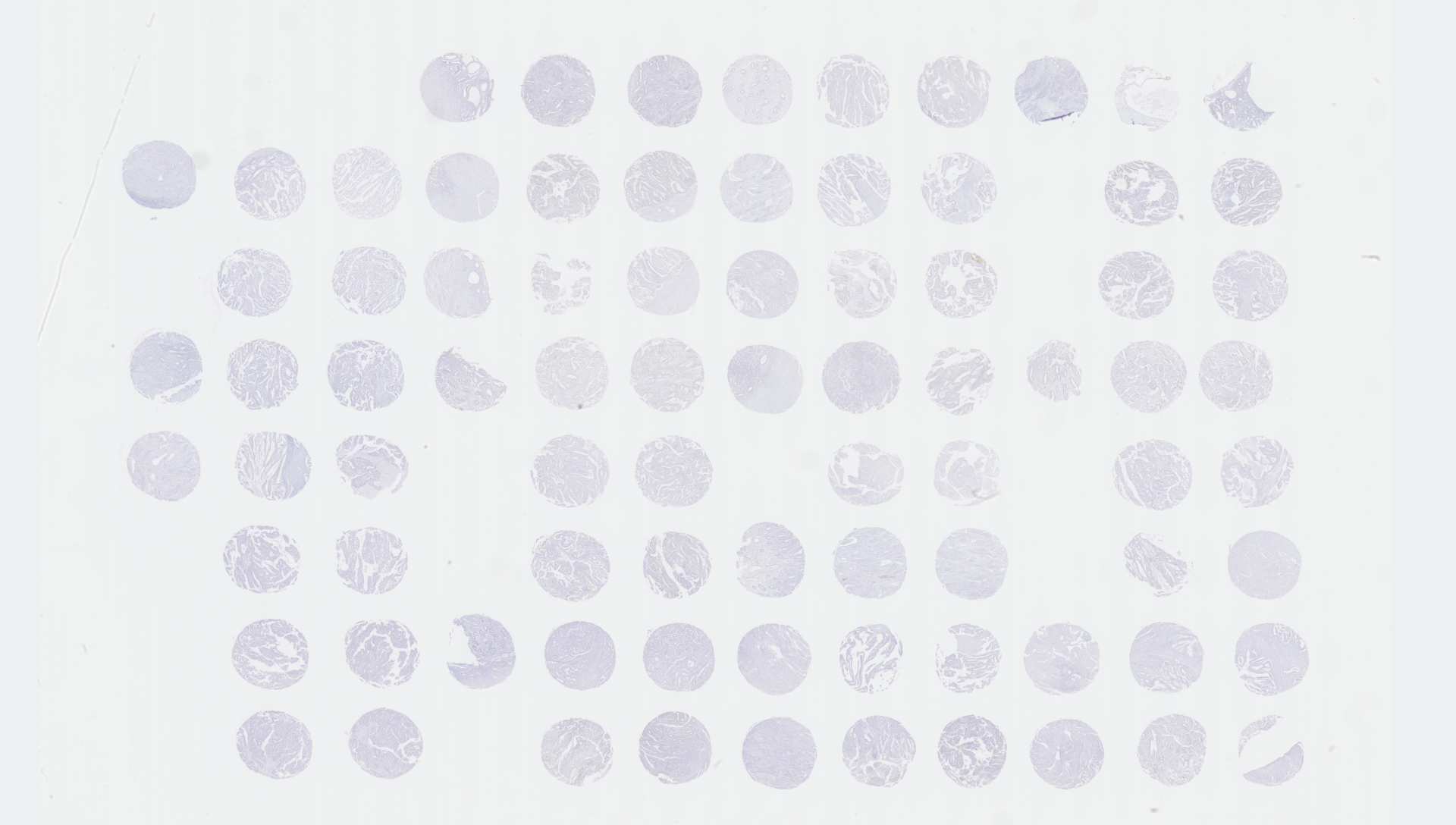

Supplement: Supplementary file 1 [file cancers-18-00198-s001.zip › Figure S1 and S2 Kurosu Original Images for Blots or Gels or Microscopy/Figure18_L1CAM2.tif]

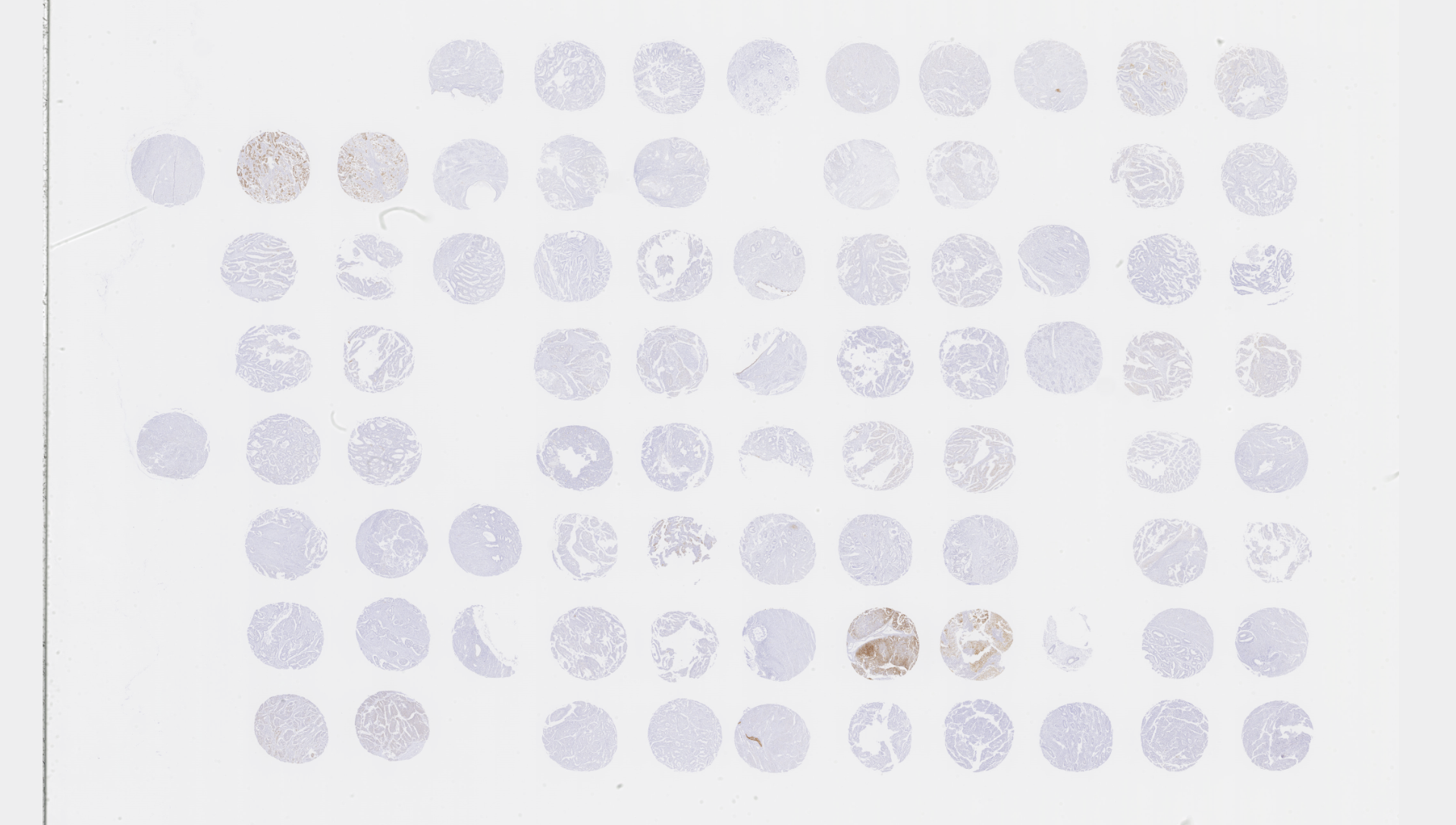

Supplement: Supplementary file 1 [file cancers-18-00198-s001.zip › Figure S1 and S2 Kurosu Original Images for Blots or Gels or Microscopy/Figure18_L1CAM3.tif]

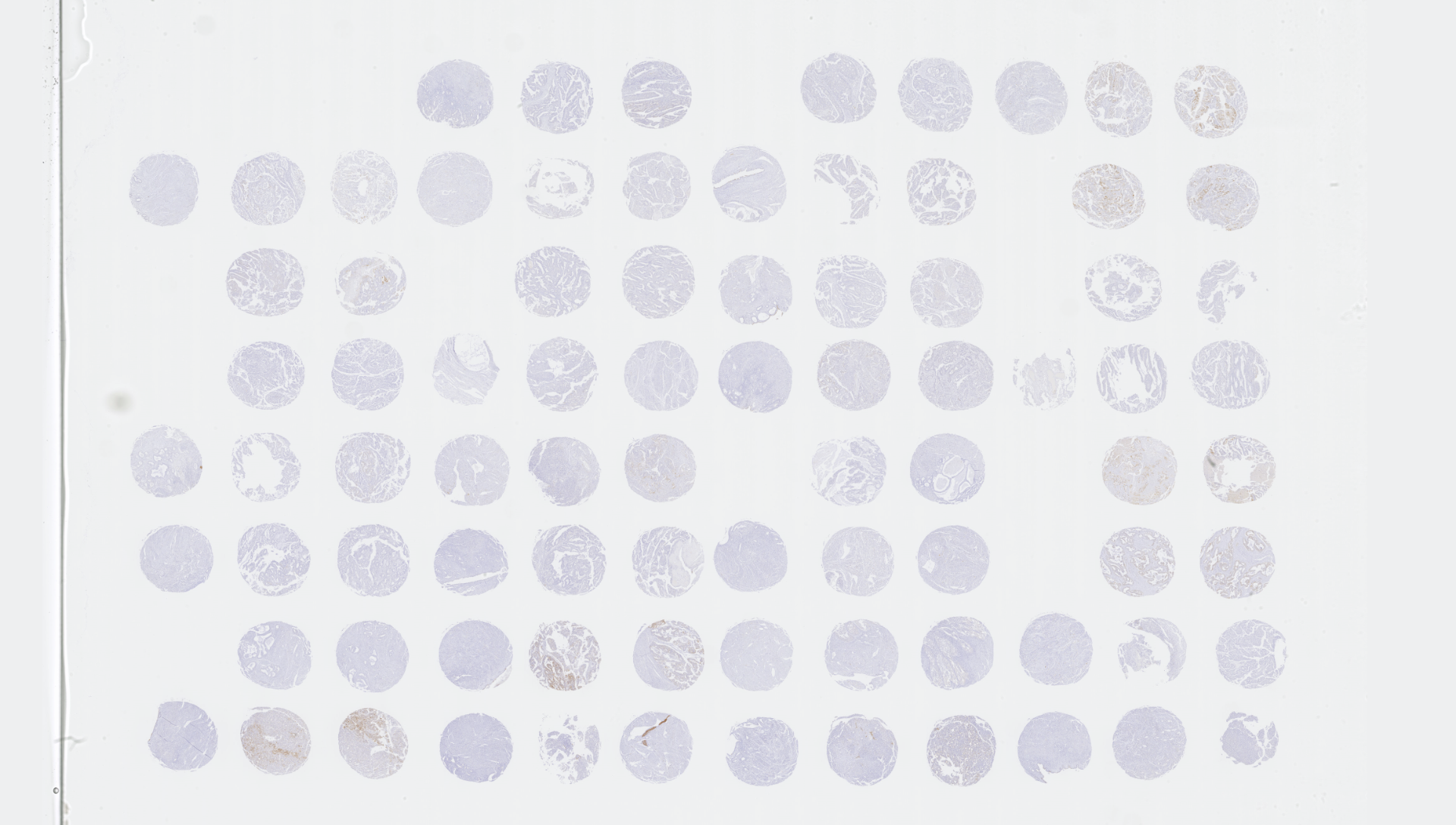

Supplement: Supplementary file 1 [file cancers-18-00198-s001.zip › Figure S1 and S2 Kurosu Original Images for Blots or Gels or Microscopy/Figure18_L1CAM4.tif]

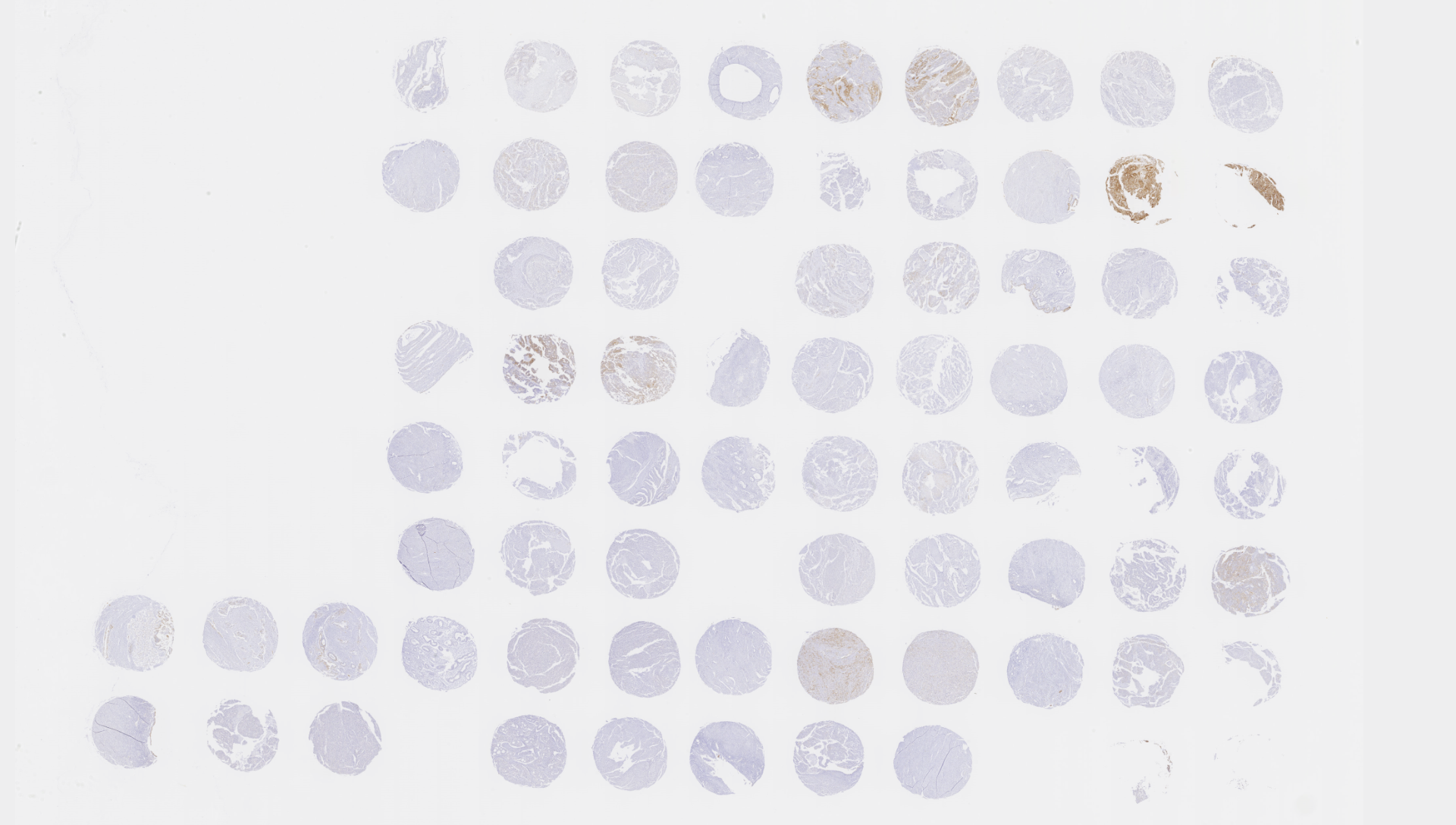

Supplement: Supplementary file 1 [file cancers-18-00198-s001.zip › Figure S1 and S2 Kurosu Original Images for Blots or Gels or Microscopy/Figure18_L1CAM5.tif]

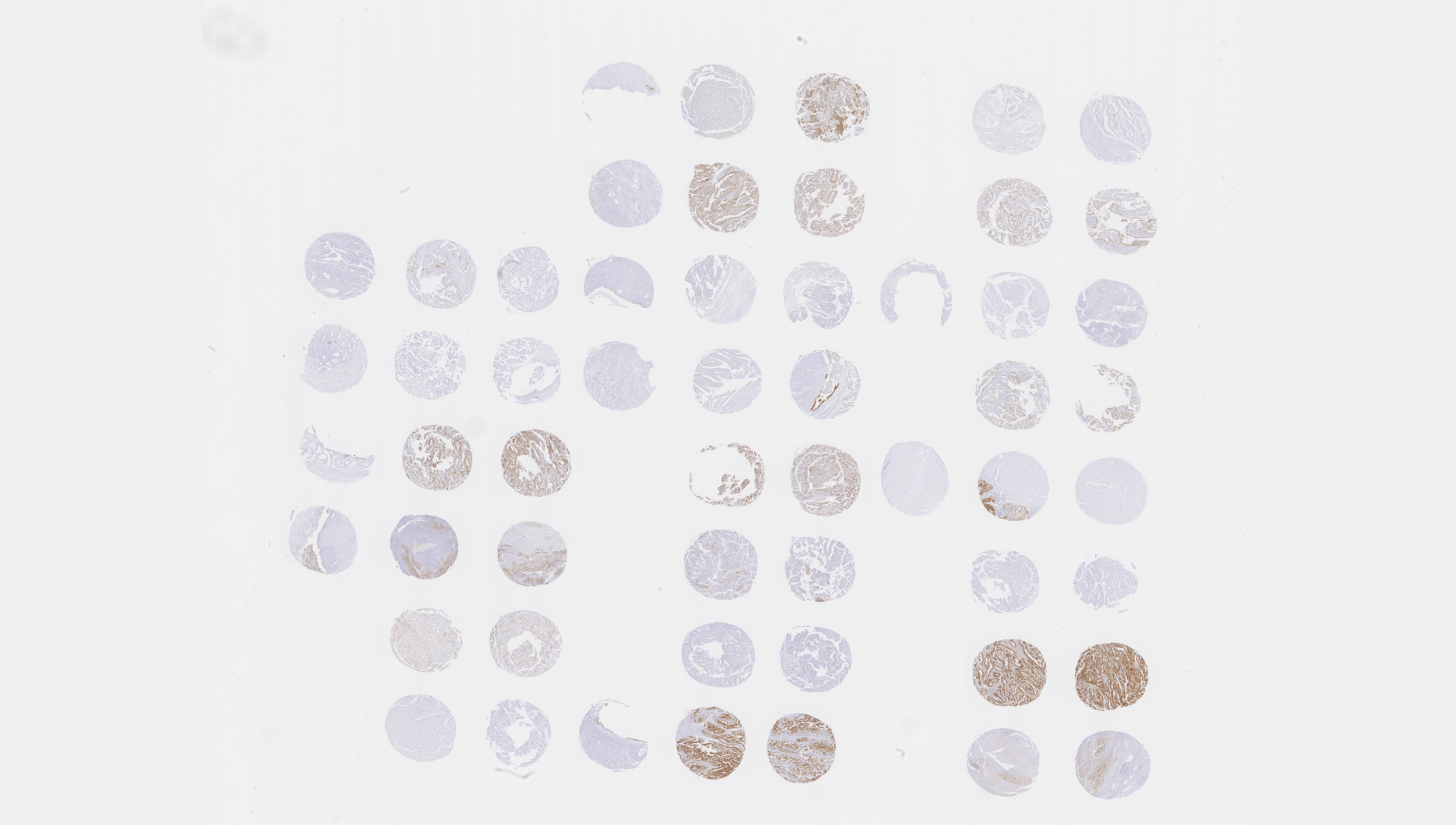

Supplement: Supplementary file 1 [file cancers-18-00198-s001.zip › Figure S1 and S2 Kurosu Original Images for Blots or Gels or Microscopy/Figure18_L1CAM6.tif]

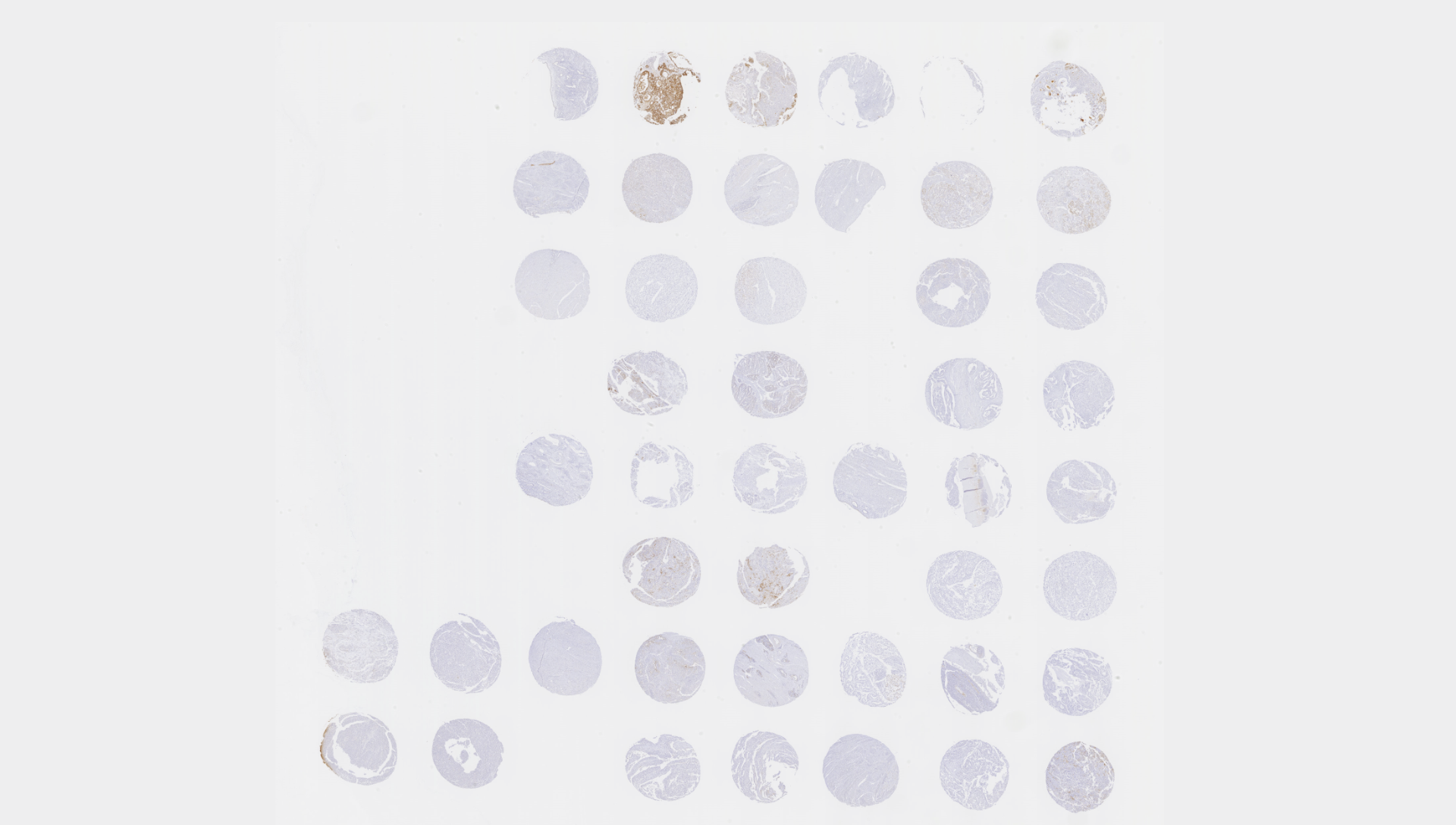

Supplement: Supplementary file 1 [file cancers-18-00198-s001.zip › Figure S1 and S2 Kurosu Original Images for Blots or Gels or Microscopy/FIgure18_L1CAM7.tif]

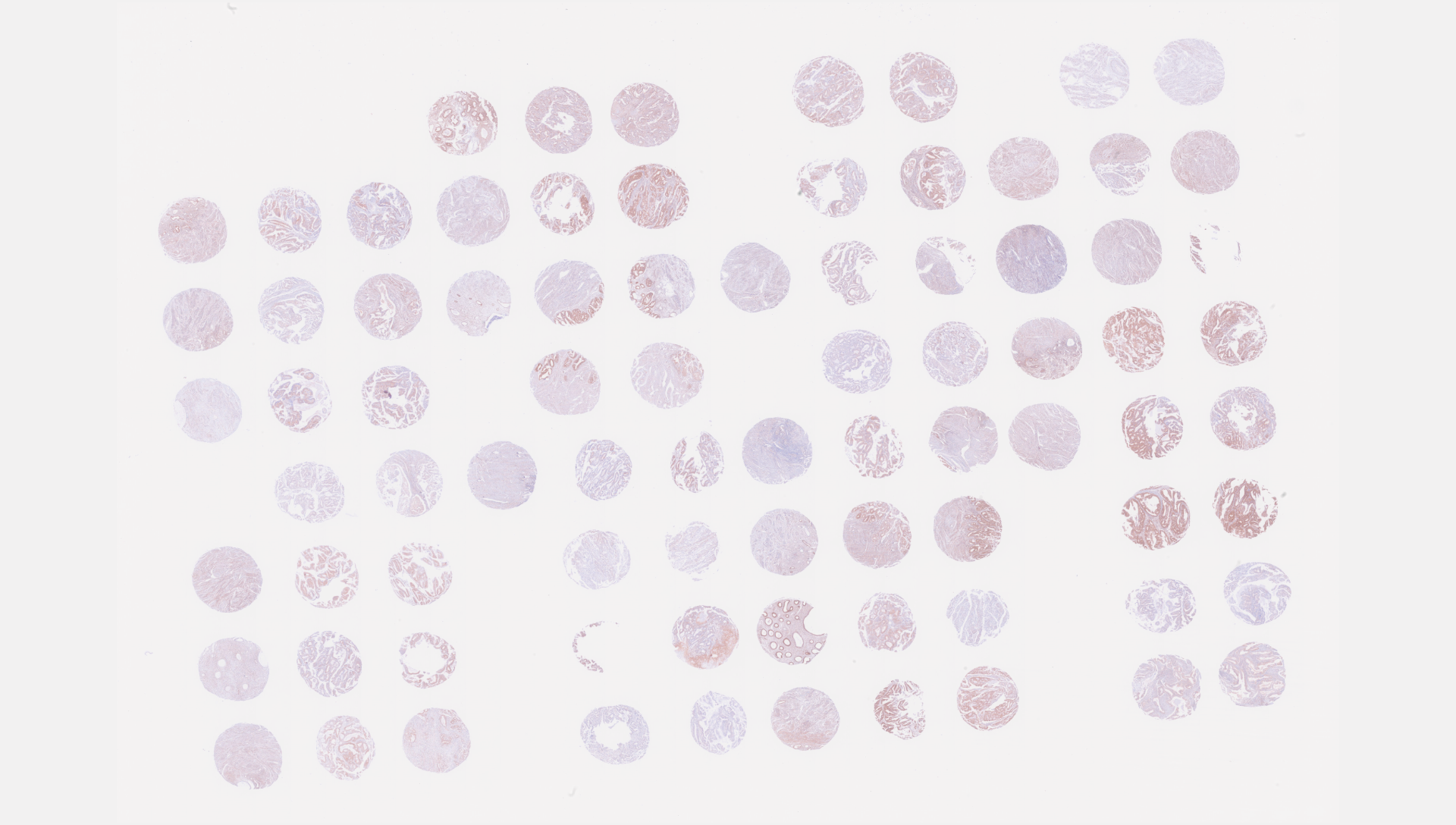

Supplement: Supplementary file 1 [file cancers-18-00198-s001.zip › Figure S1 and S2 Kurosu Original Images for Blots or Gels or Microscopy/Figure18_NF-kB(p65)1.tif]

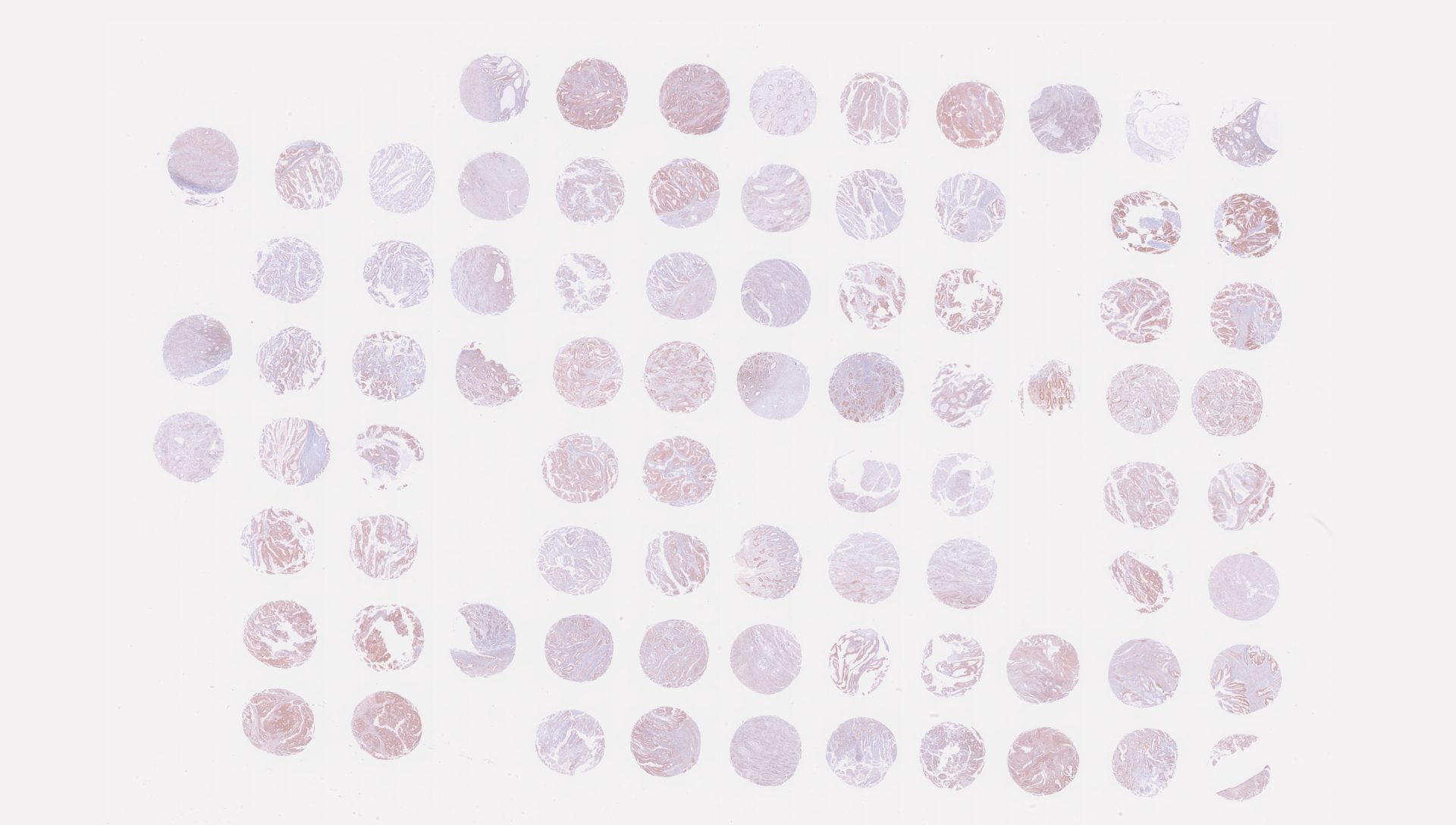

Supplement: Supplementary file 1 [file cancers-18-00198-s001.zip › Figure S1 and S2 Kurosu Original Images for Blots or Gels or Microscopy/Figure18_NF-kB(p65)2.tif]

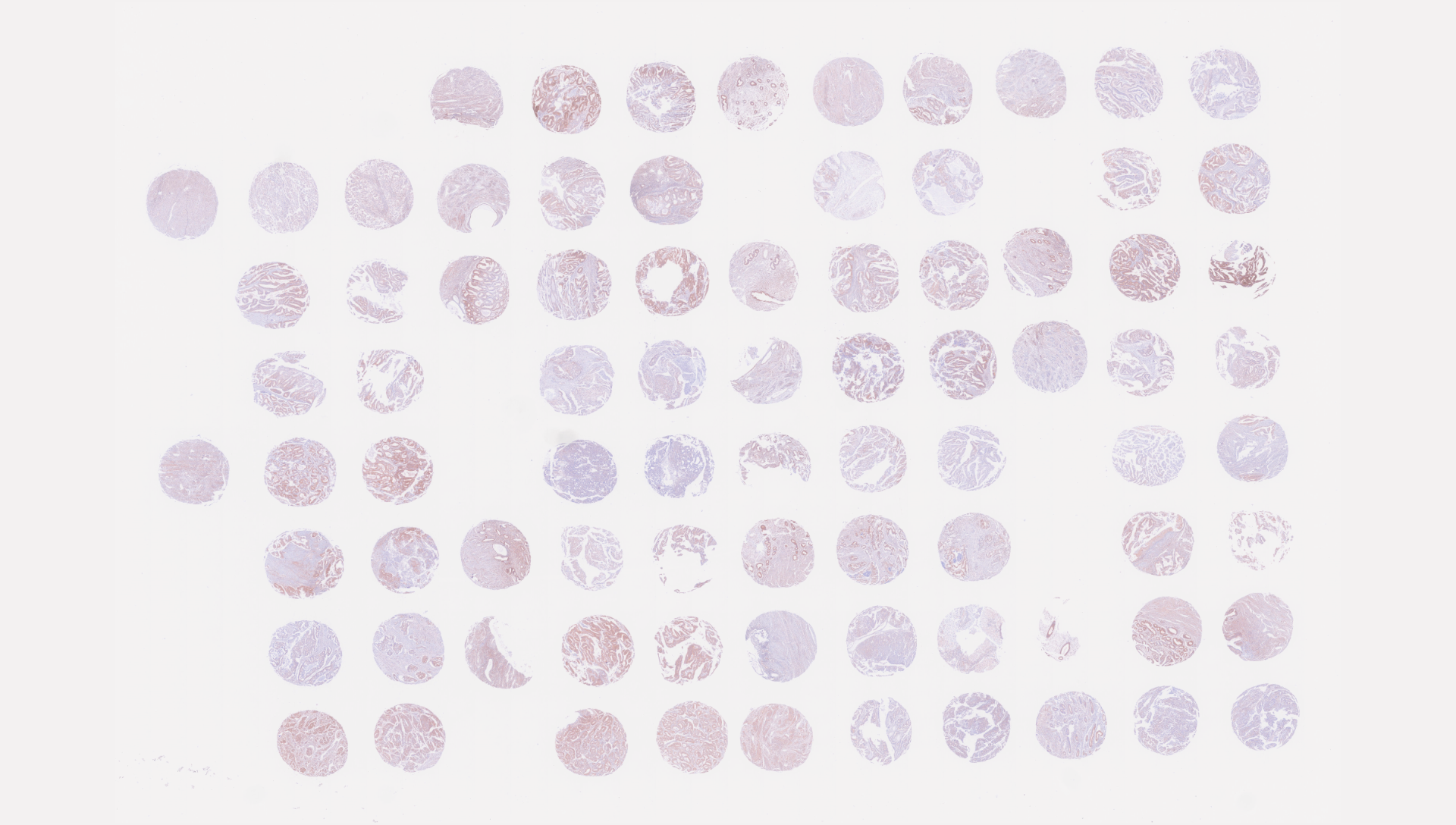

Supplement: Supplementary file 1 [file cancers-18-00198-s001.zip › Figure S1 and S2 Kurosu Original Images for Blots or Gels or Microscopy/Figure18_NF-kB(p65)3.tif]

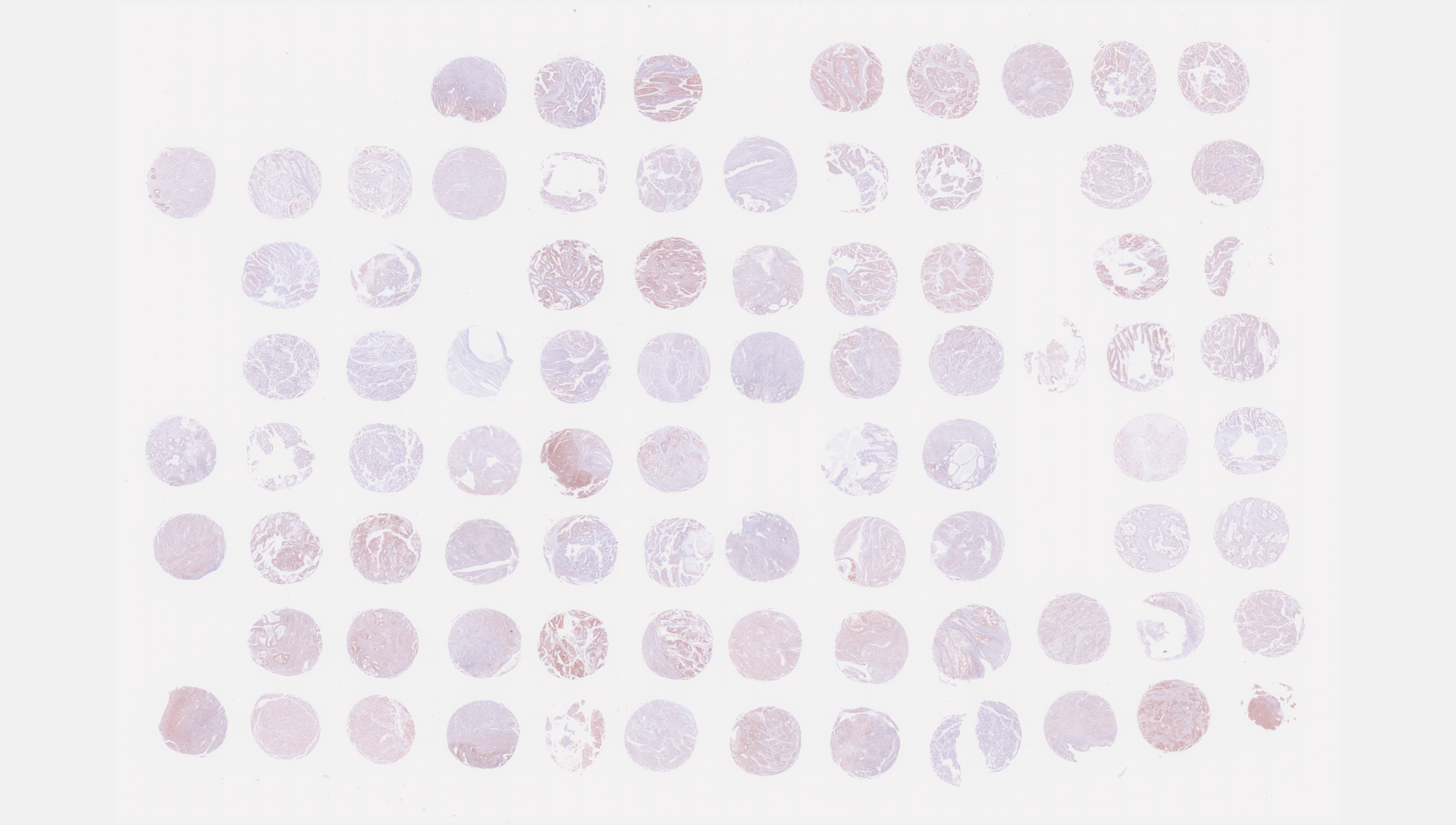

Supplement: Supplementary file 1 [file cancers-18-00198-s001.zip › Figure S1 and S2 Kurosu Original Images for Blots or Gels or Microscopy/Figure18_NF-kB(p65)4.tif]

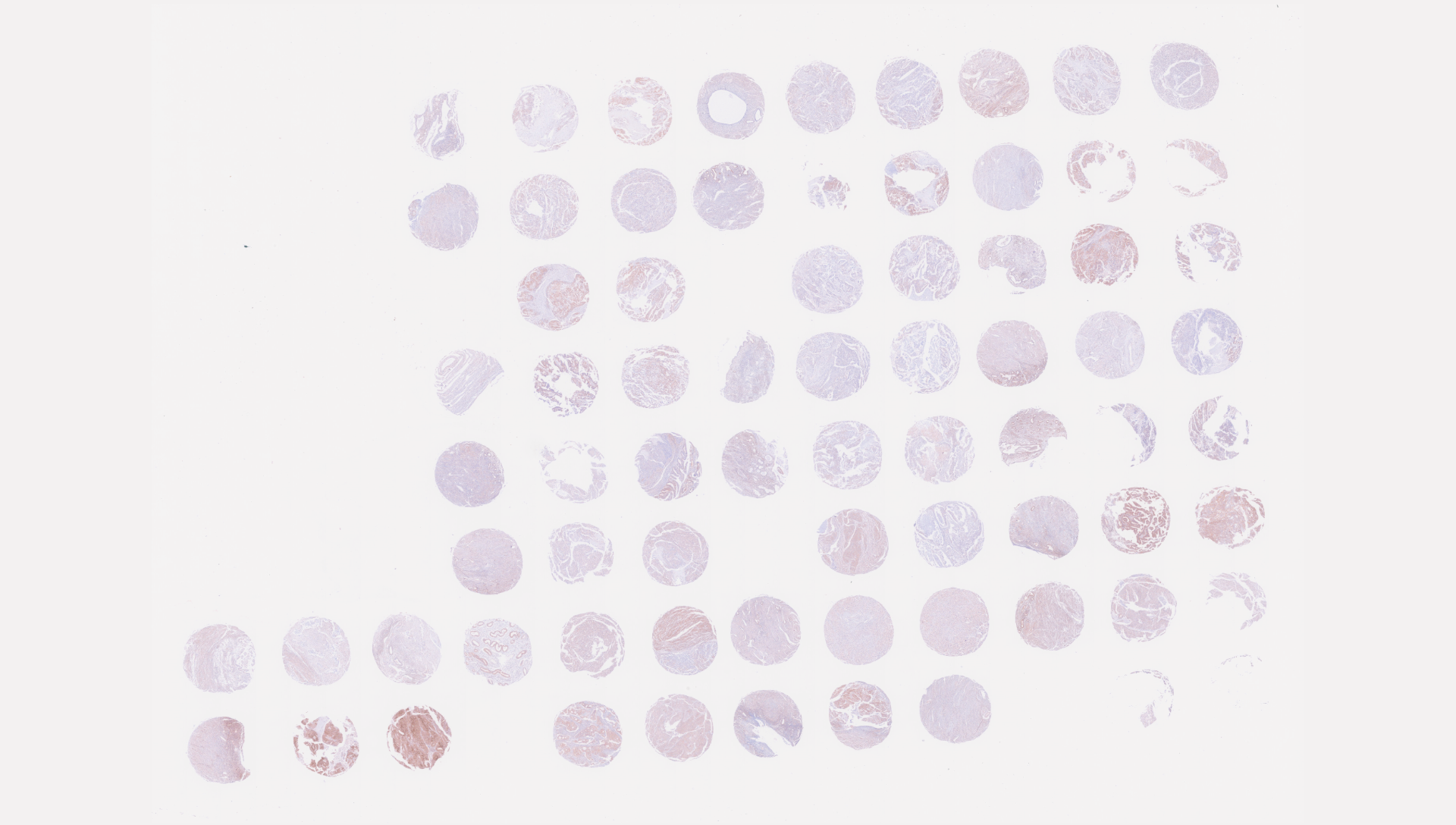

Supplement: Supplementary file 1 [file cancers-18-00198-s001.zip › Figure S1 and S2 Kurosu Original Images for Blots or Gels or Microscopy/Figure18_NF-kB(p65)5.tif]

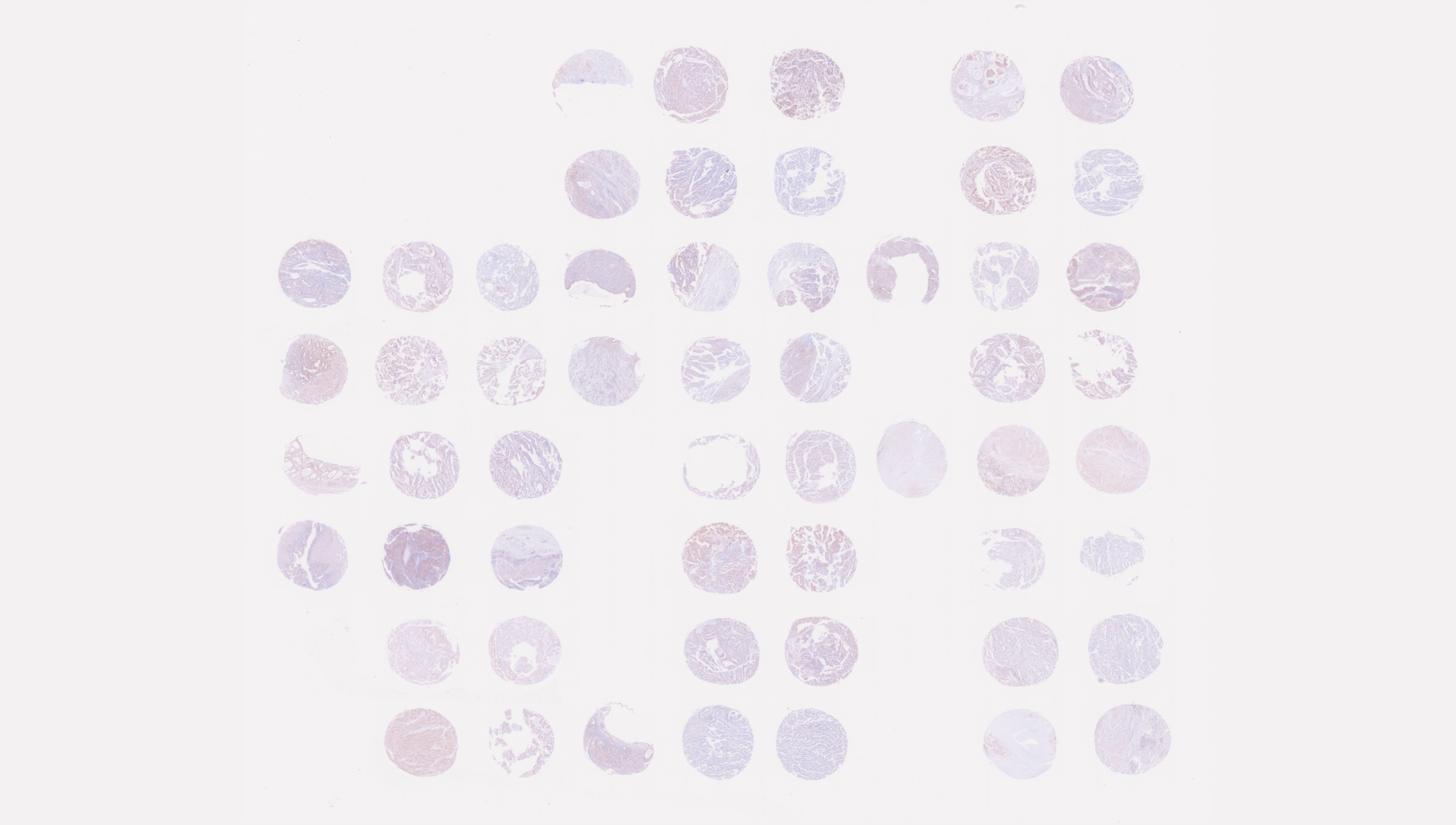

Supplement: Supplementary file 1 [file cancers-18-00198-s001.zip › Figure S1 and S2 Kurosu Original Images for Blots or Gels or Microscopy/Figure18_NF-kB(p65)6.tif]

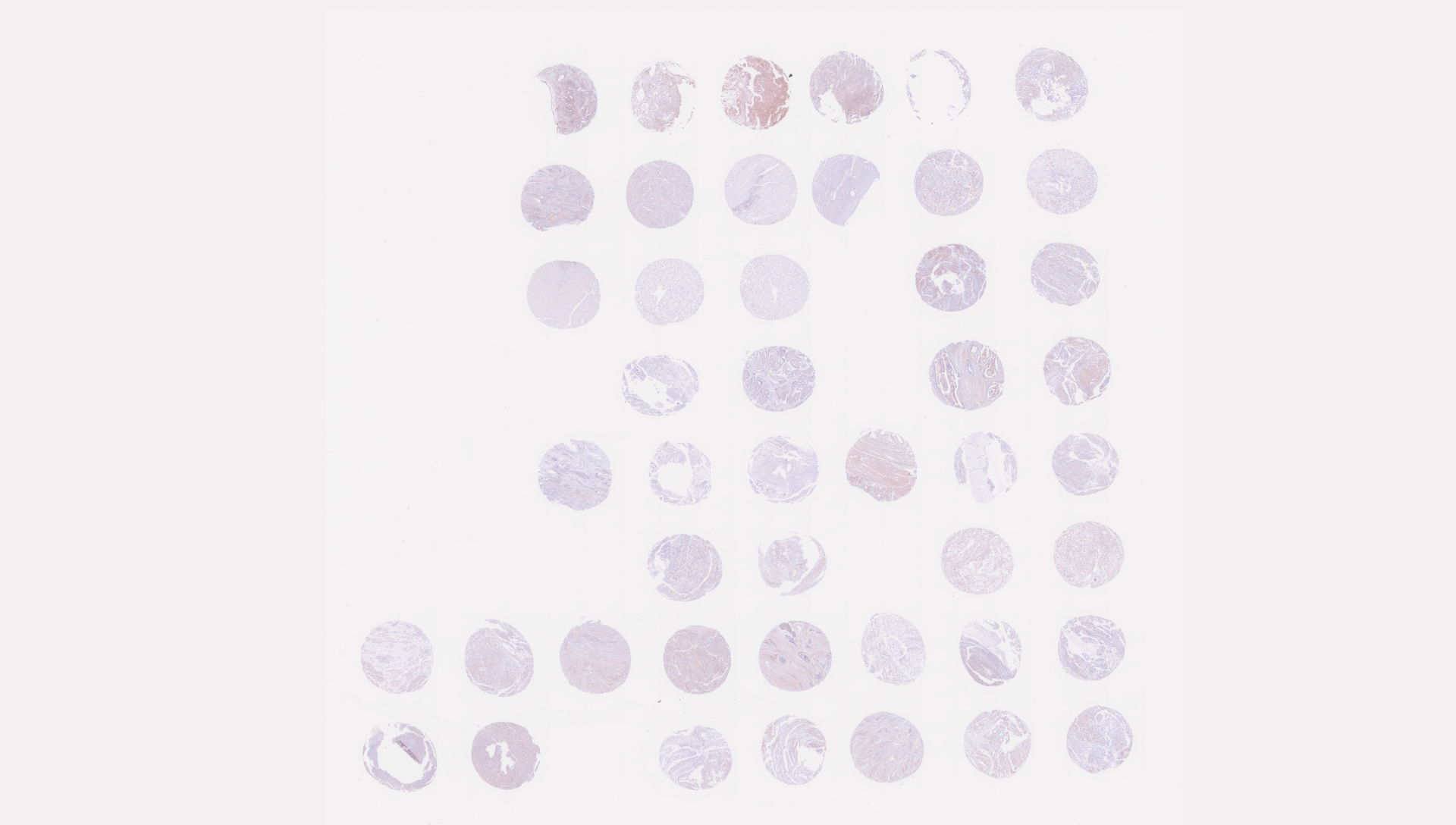

Supplement: Supplementary file 1 [file cancers-18-00198-s001.zip › Figure S1 and S2 Kurosu Original Images for Blots or Gels or Microscopy/Figure18_NF-kB(p65)7.tif]

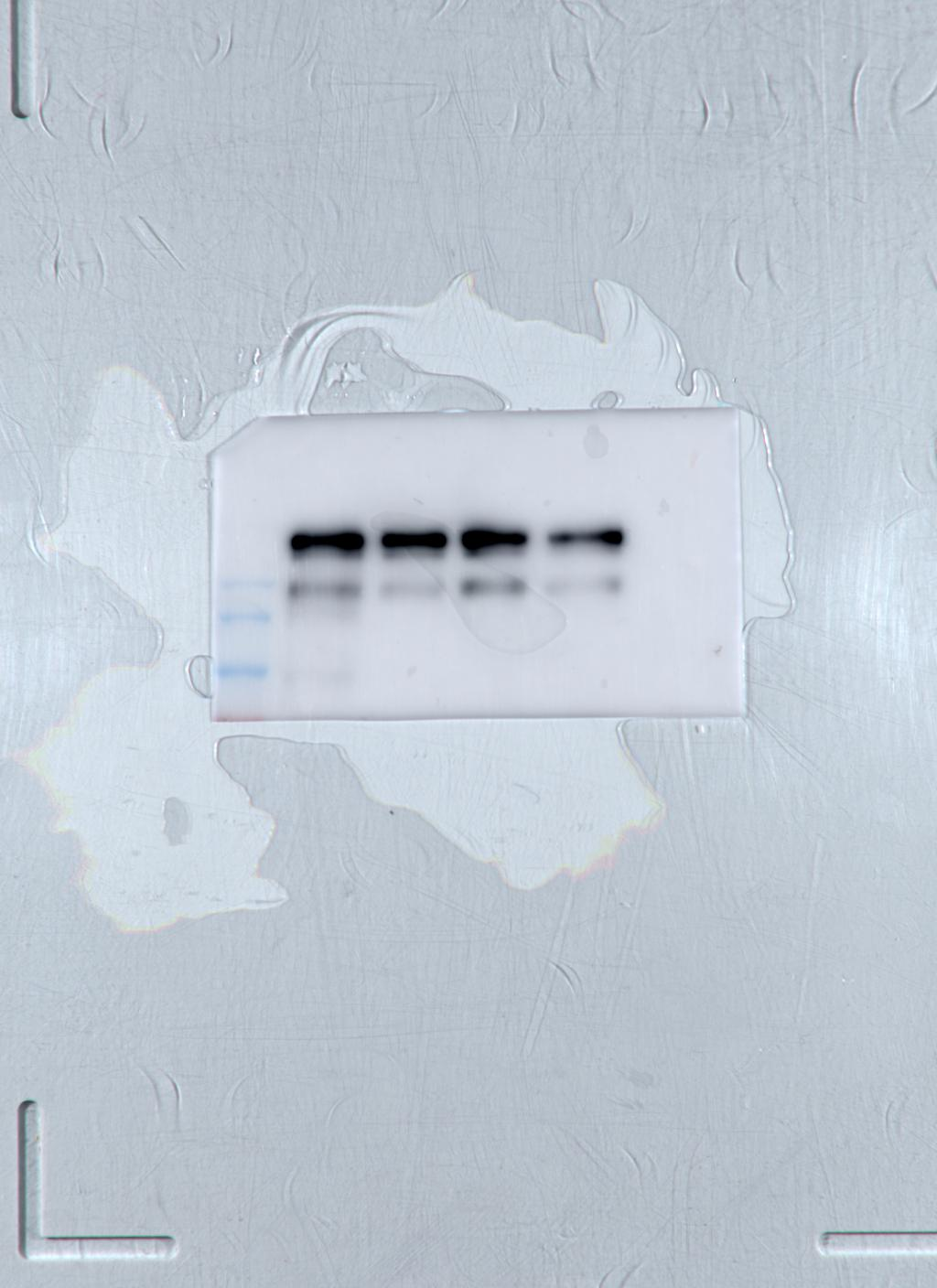

Supplement: Supplementary file 1 [file cancers-18-00198-s001.zip › Figure S1 and S2 Kurosu Original Images for Blots or Gels or Microscopy/Figure19 HHUA_L1CAM.tif]

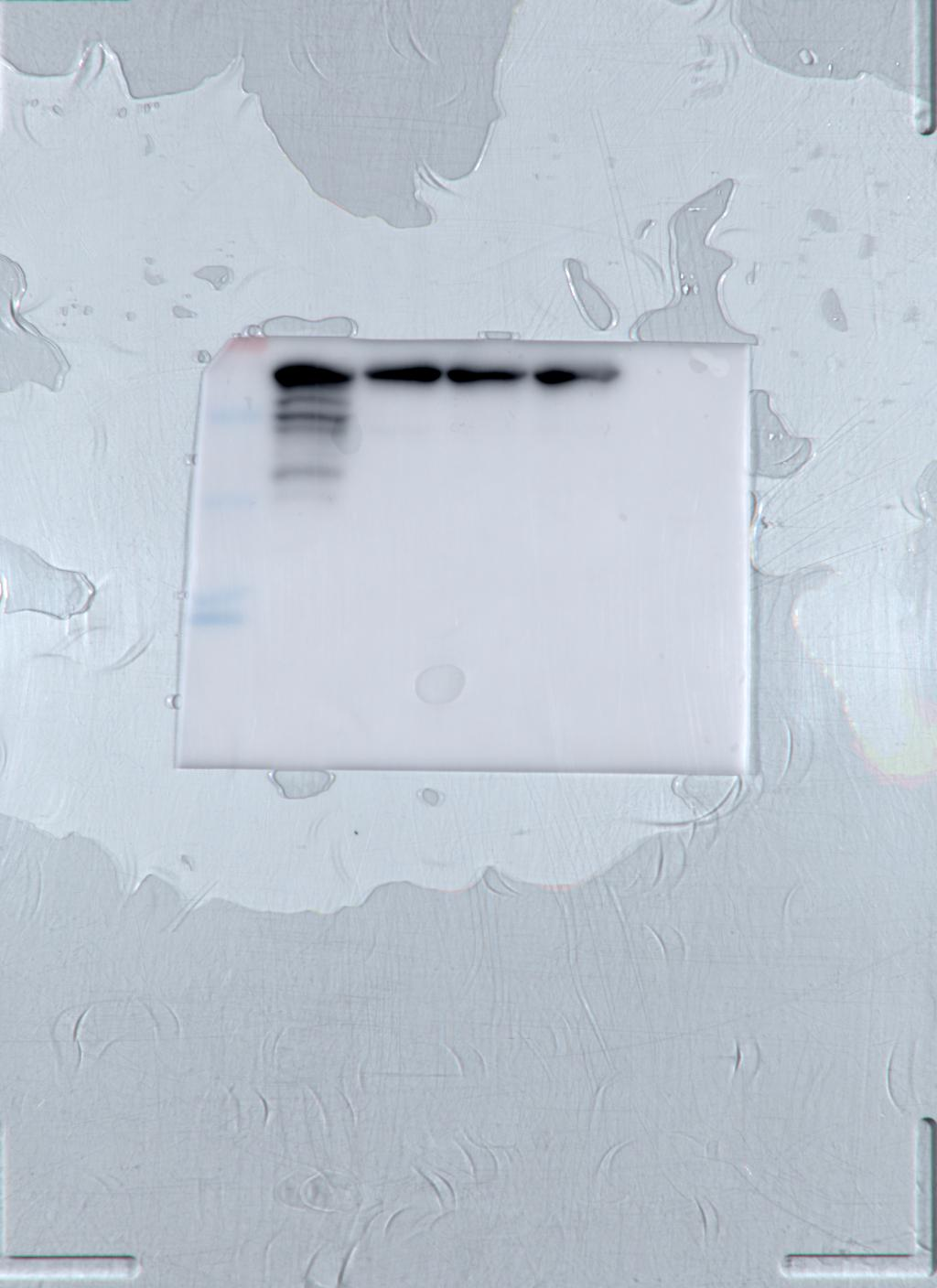

Supplement: Supplementary file 1 [file cancers-18-00198-s001.zip › Figure S1 and S2 Kurosu Original Images for Blots or Gels or Microscopy/Figure19 HHUA_NF-kB(p65).tif]

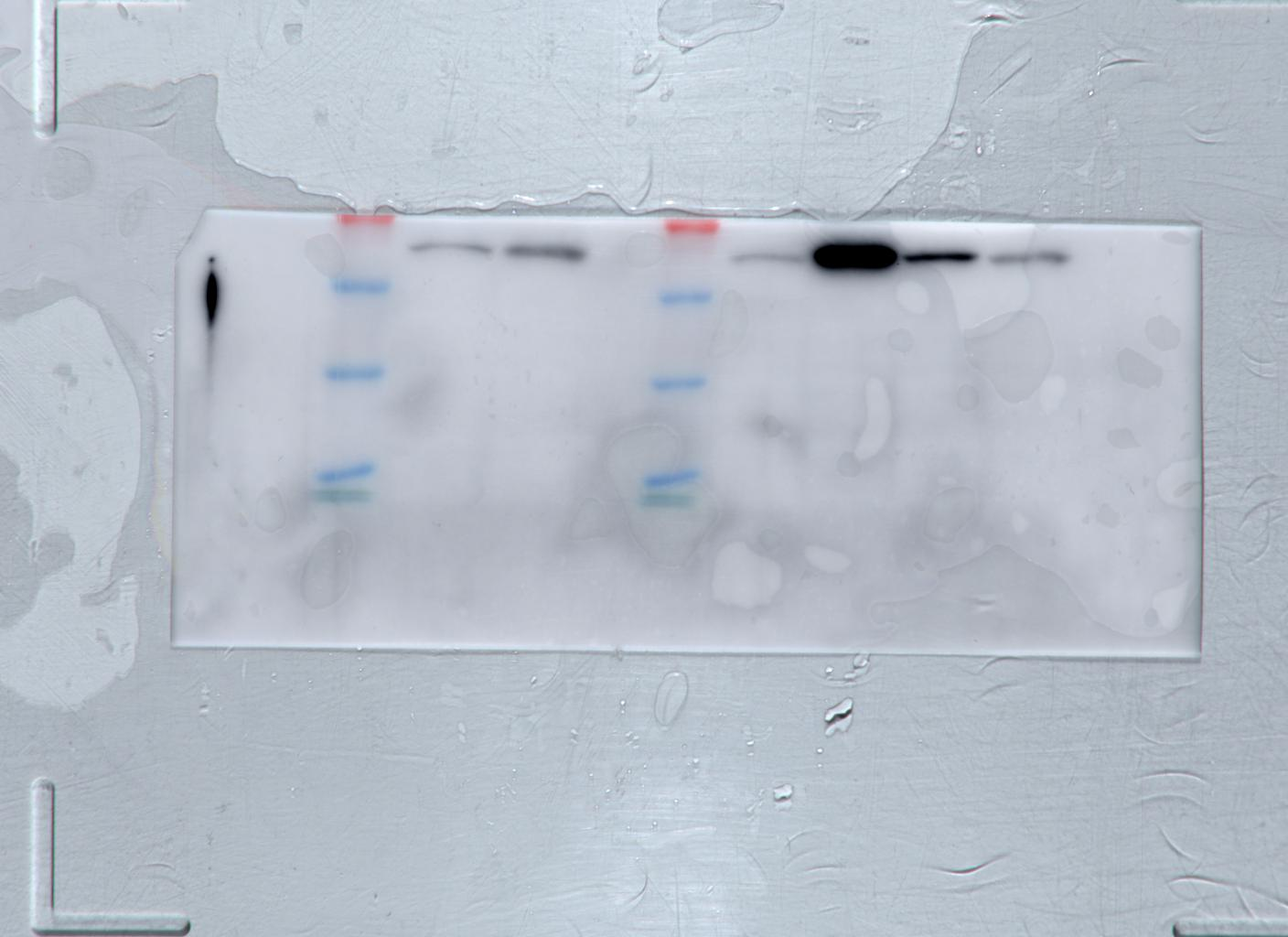

Supplement: Supplementary file 1 [file cancers-18-00198-s001.zip › Figure S1 and S2 Kurosu Original Images for Blots or Gels or Microscopy/Figure19 HHUA_pNF-kB(p65) (the 4 bands on the right).tif]

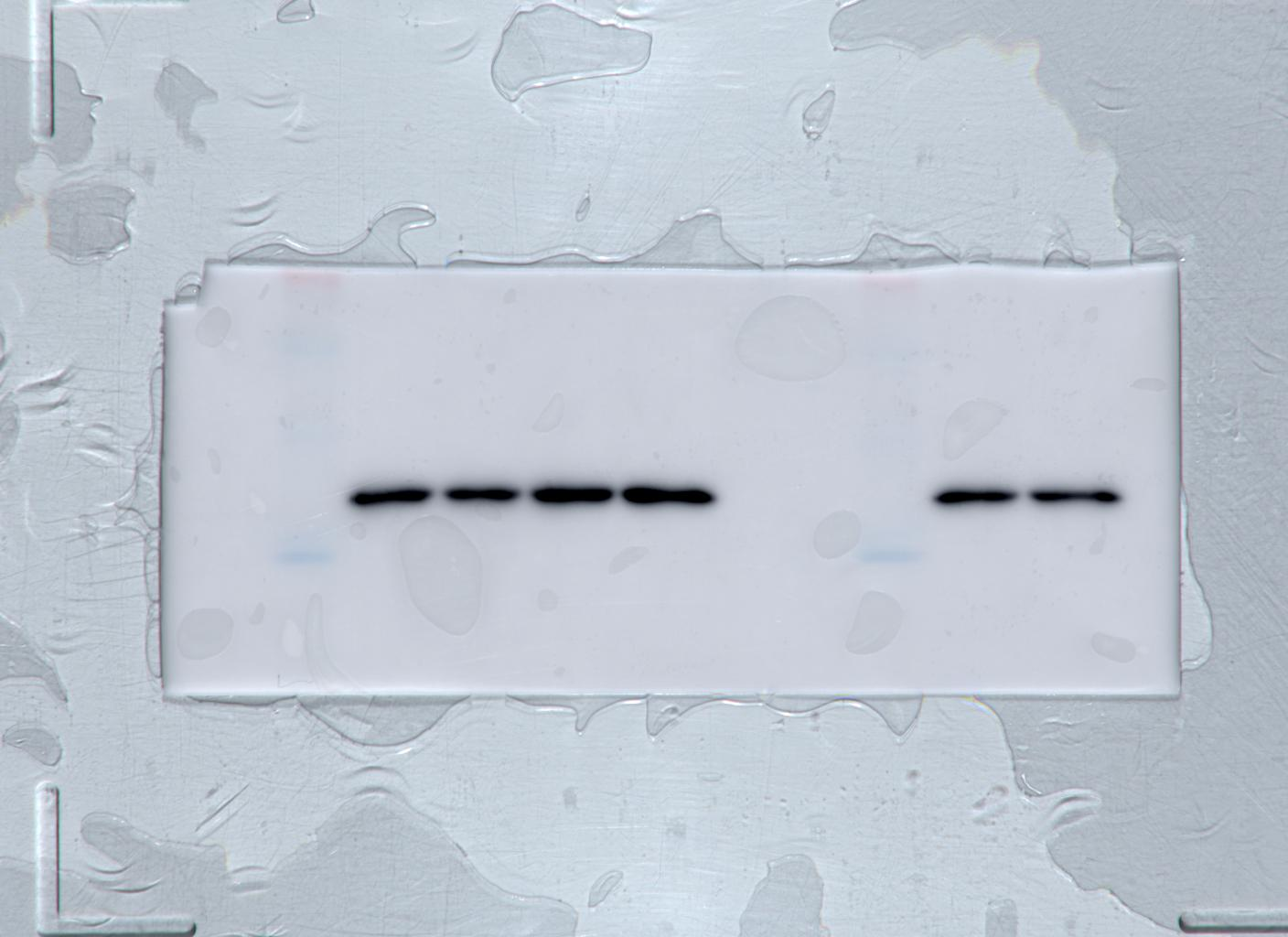

Supplement: Supplementary file 1 [file cancers-18-00198-s001.zip › Figure S1 and S2 Kurosu Original Images for Blots or Gels or Microscopy/Figure19 SPAC-1-L_GAPDH(p65) (the 4 bands on the left).tif]

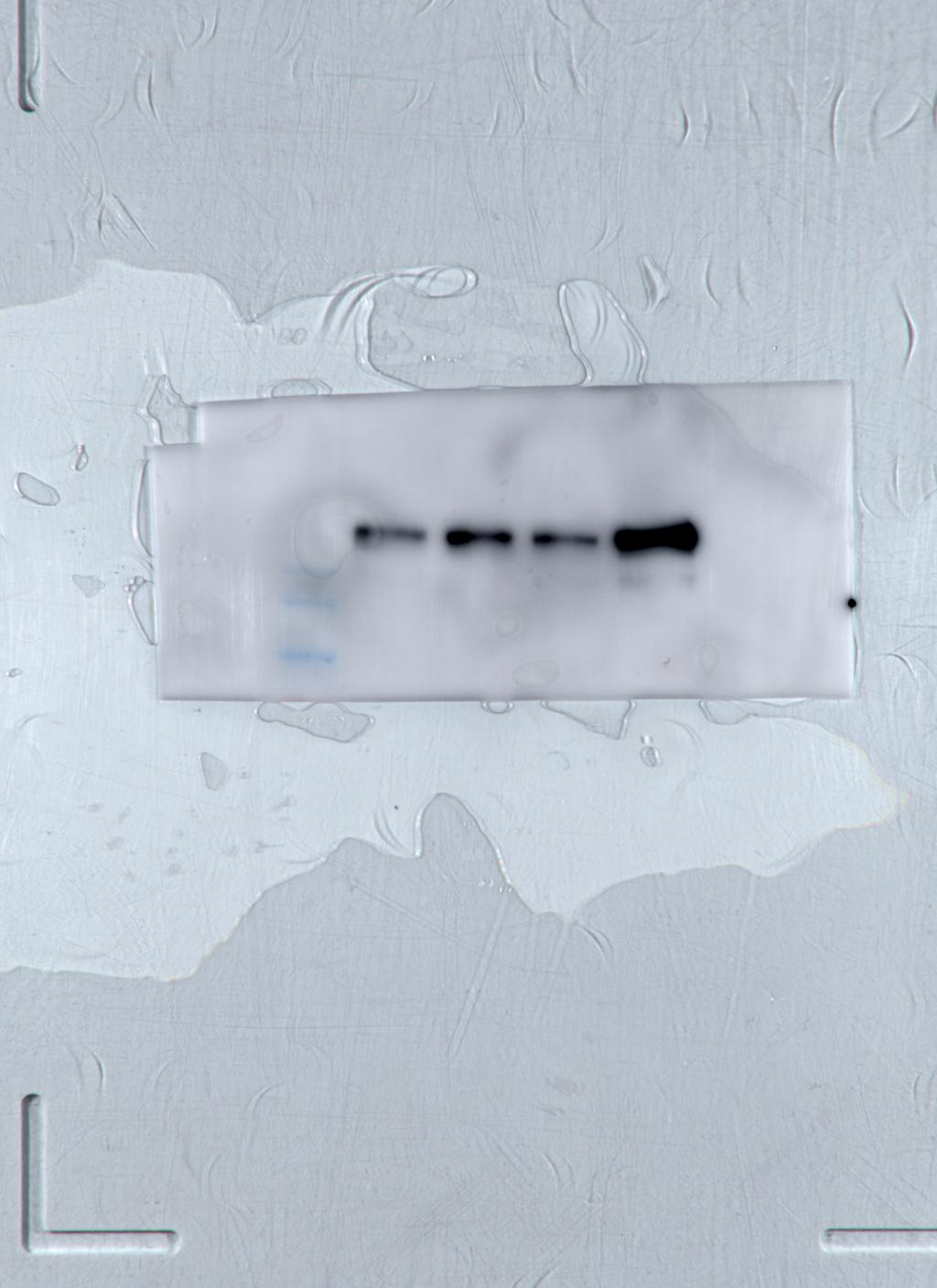

Supplement: Supplementary file 1 [file cancers-18-00198-s001.zip › Figure S1 and S2 Kurosu Original Images for Blots or Gels or Microscopy/Figure19 SPAC-1-L_L1CAM.tif]

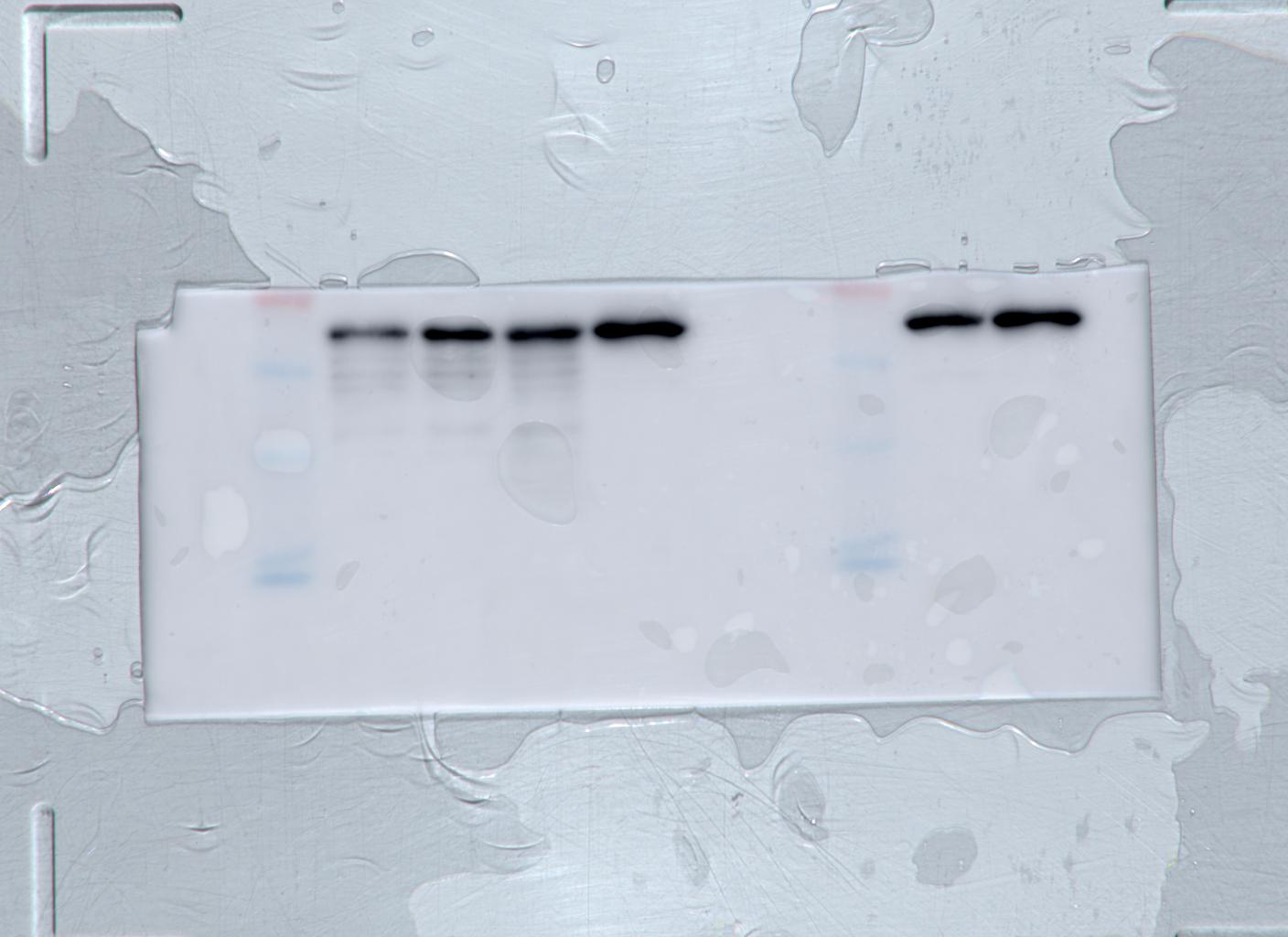

Supplement: Supplementary file 1 [file cancers-18-00198-s001.zip › Figure S1 and S2 Kurosu Original Images for Blots or Gels or Microscopy/Figure19 SPAC-1-L_NF-kB(p65) (the 4 bands on the left).tif]

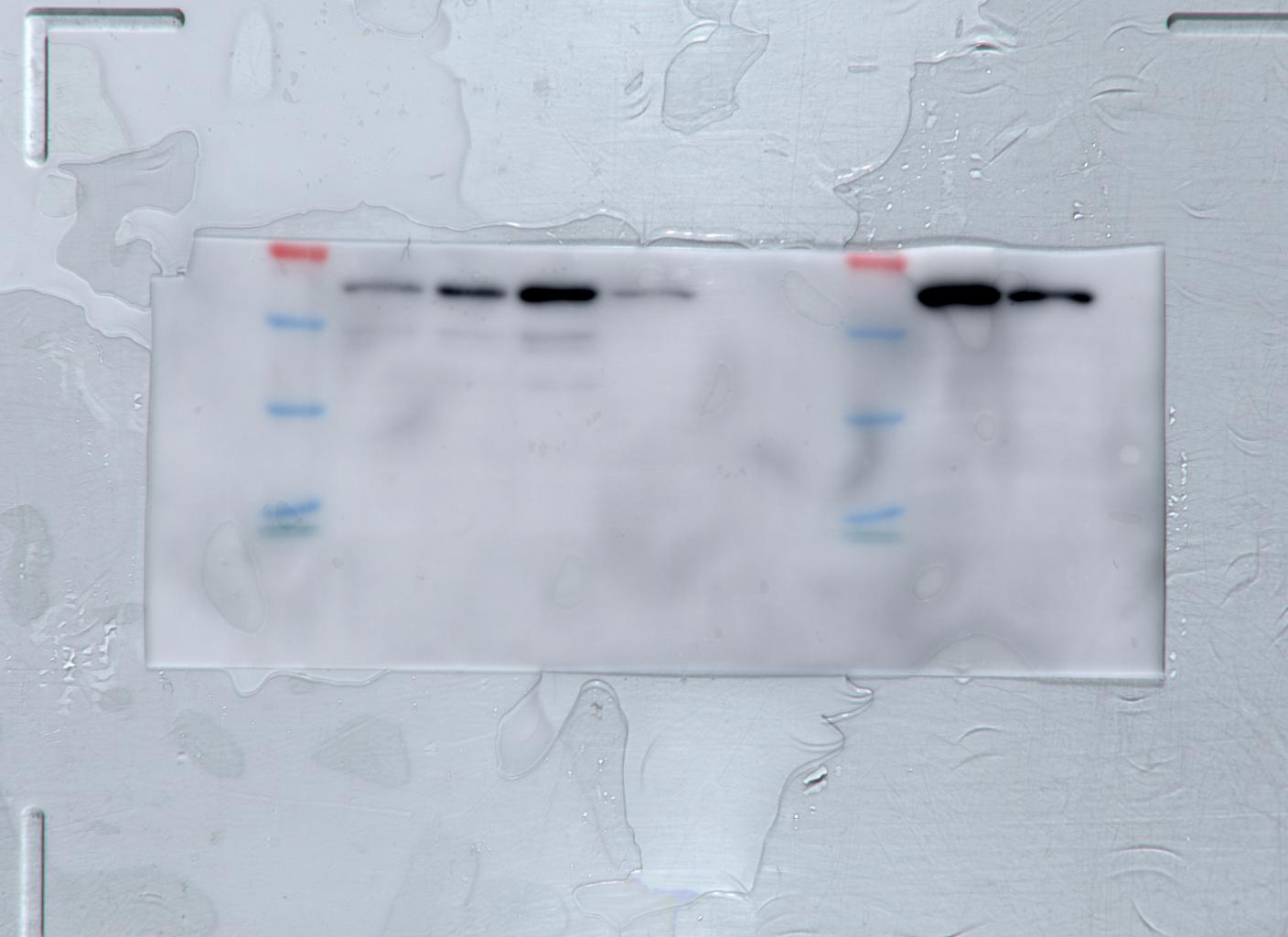

Supplement: Supplementary file 1 [file cancers-18-00198-s001.zip › Figure S1 and S2 Kurosu Original Images for Blots or Gels or Microscopy/Figure19 SPAC-1-L_pNF-kB(p65) (the 4 bands on the left).tif]

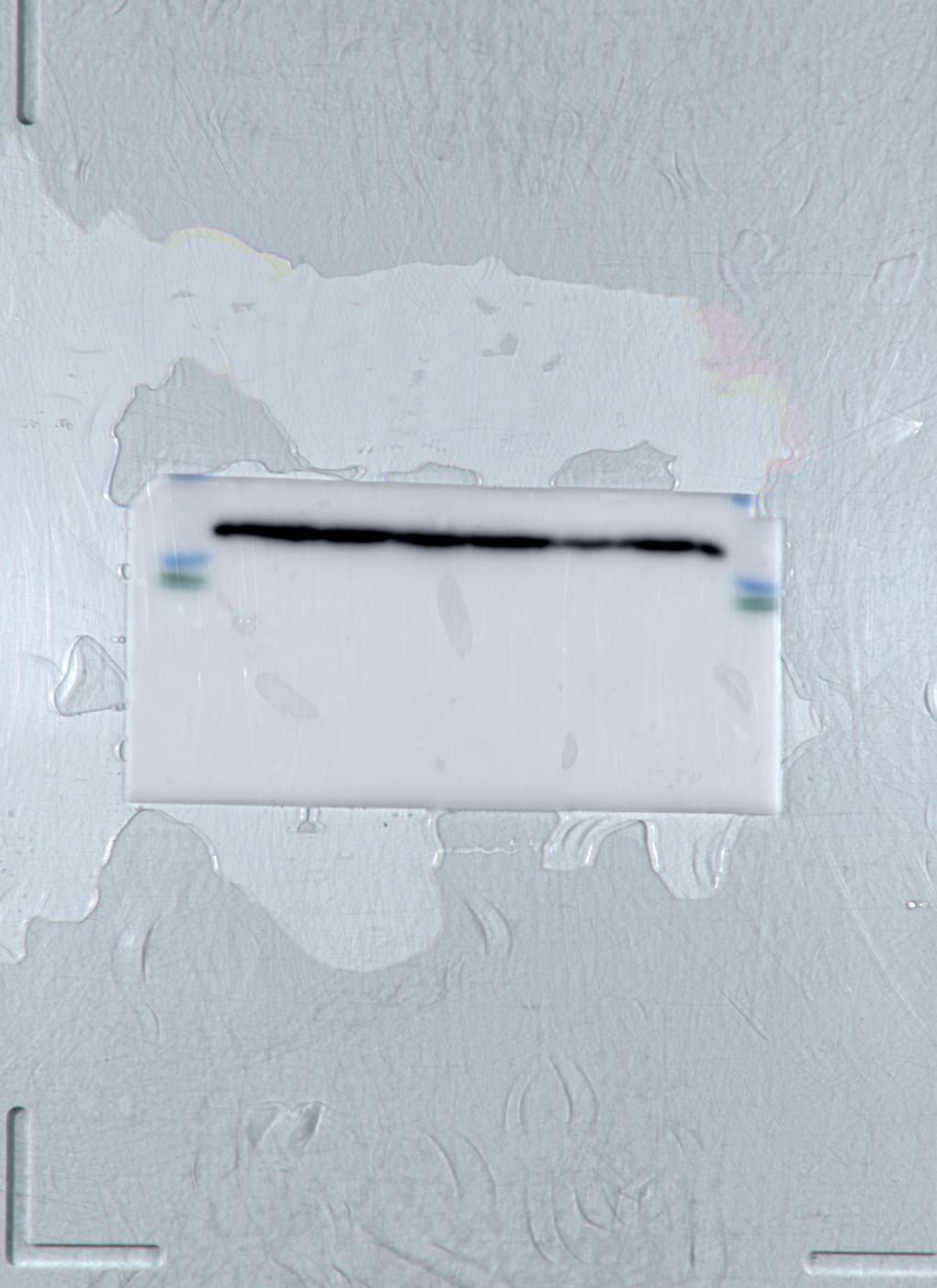

Supplement: Supplementary file 1 [file cancers-18-00198-s001.zip › Figure S1 and S2 Kurosu Original Images for Blots or Gels or Microscopy/Figure1B GAPDH.tif]

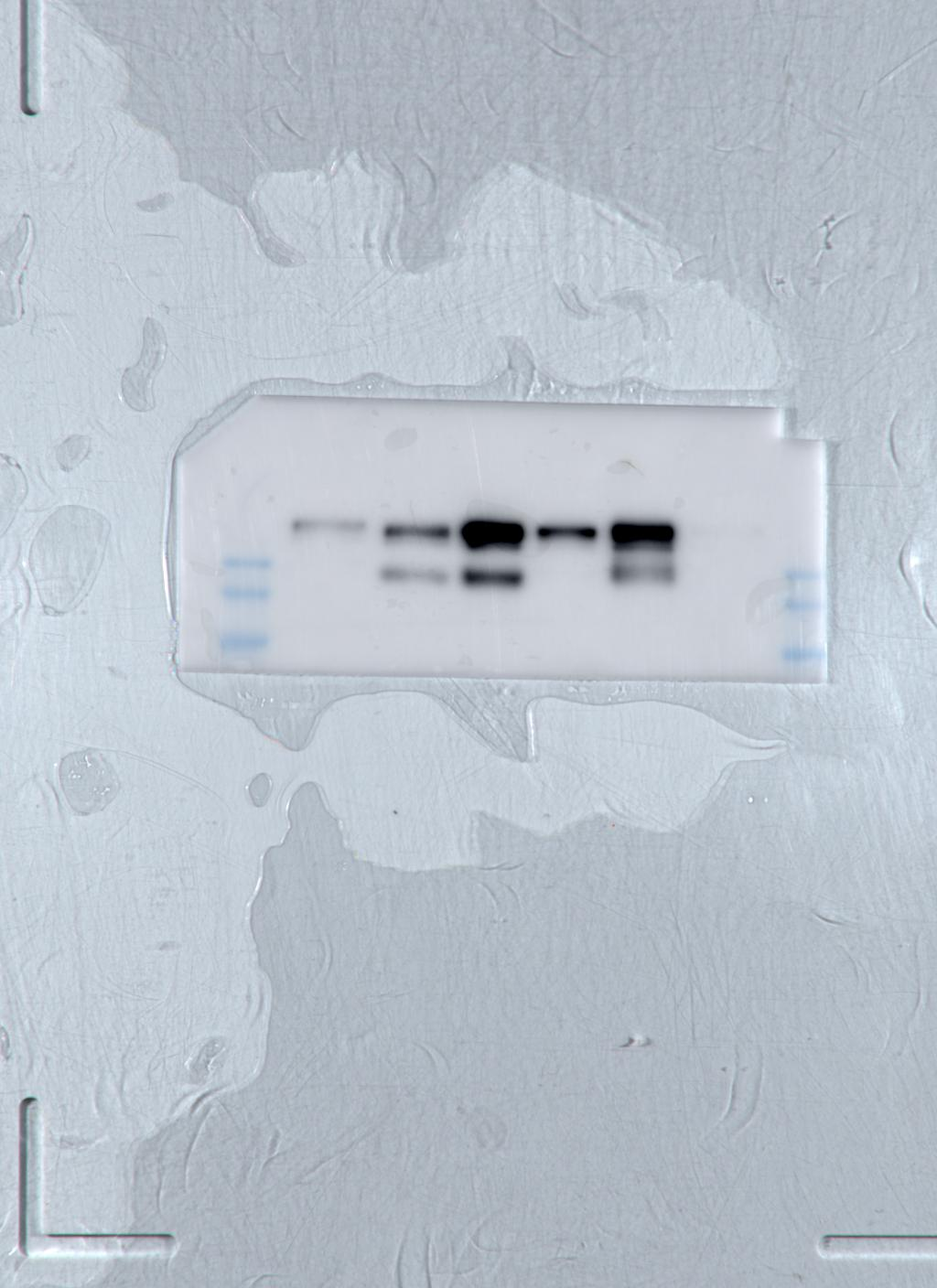

Supplement: Supplementary file 1 [file cancers-18-00198-s001.zip › Figure S1 and S2 Kurosu Original Images for Blots or Gels or Microscopy/Figure1B L1CAM.tif]

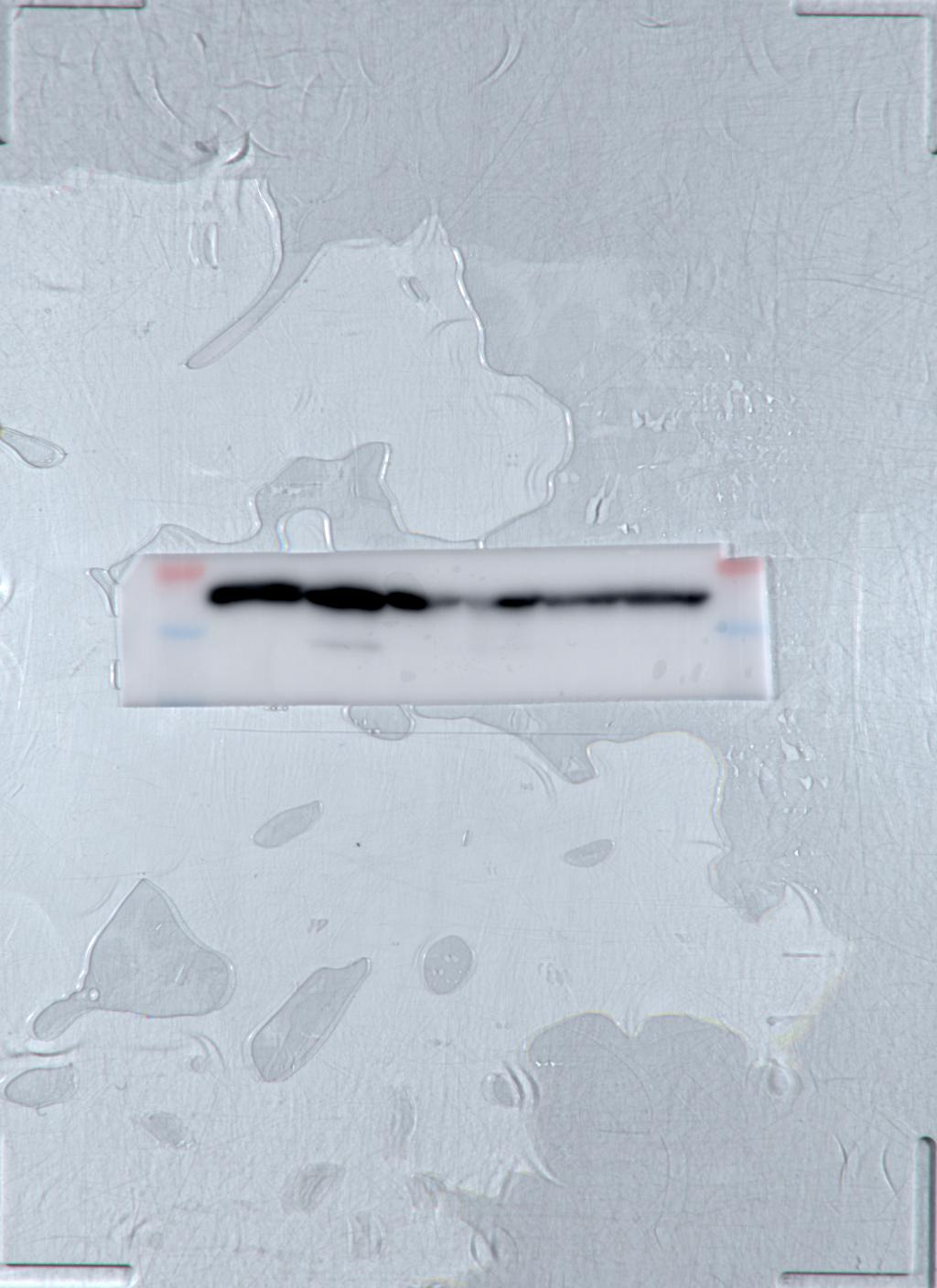

Supplement: Supplementary file 1 [file cancers-18-00198-s001.zip › Figure S1 and S2 Kurosu Original Images for Blots or Gels or Microscopy/Figure1B NF-kB(p65).tif]

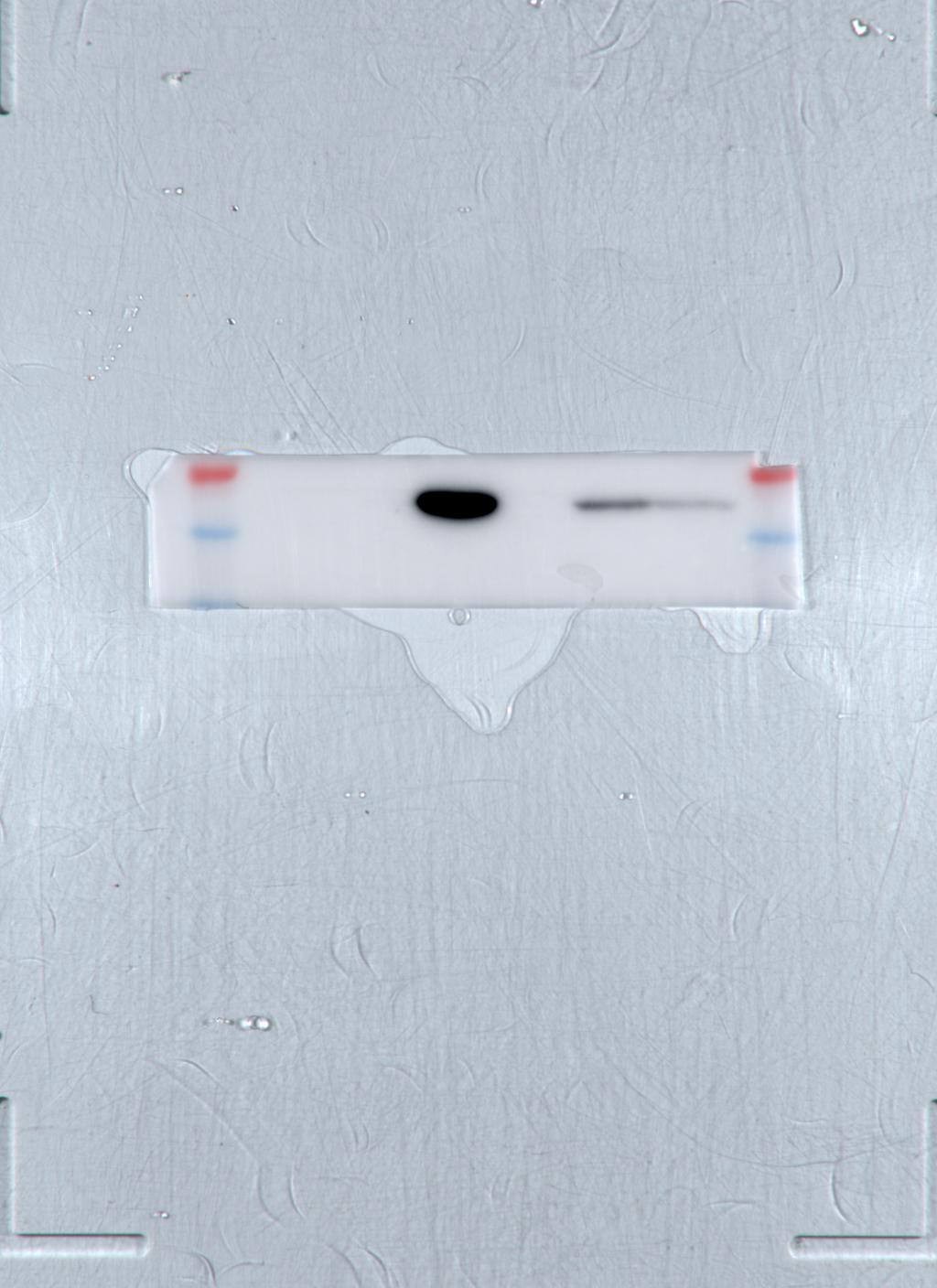

Supplement: Supplementary file 1 [file cancers-18-00198-s001.zip › Figure S1 and S2 Kurosu Original Images for Blots or Gels or Microscopy/Figure1B pNF-kB(p65).tif]

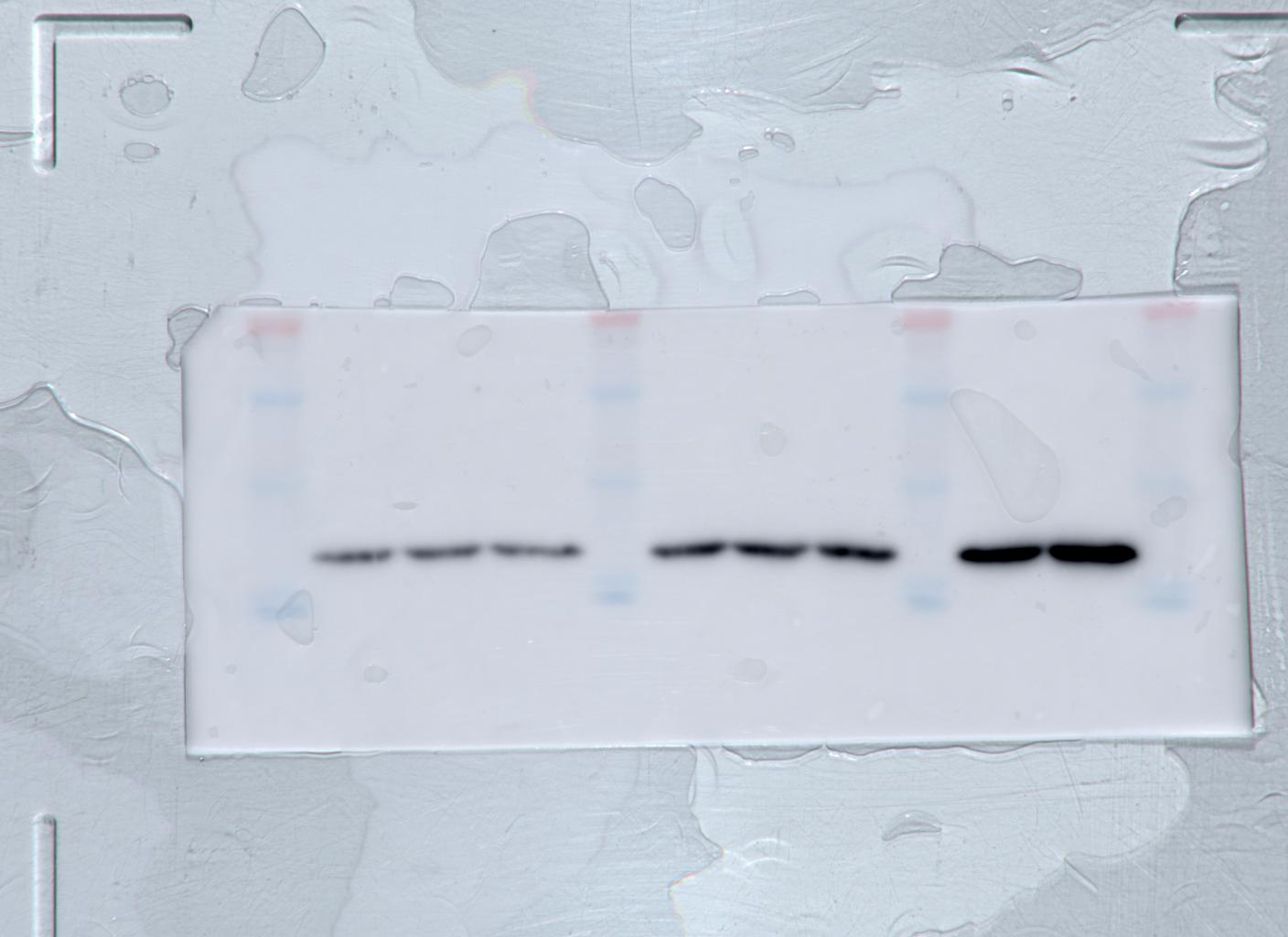

Supplement: Supplementary file 1 [file cancers-18-00198-s001.zip › Figure S1 and S2 Kurosu Original Images for Blots or Gels or Microscopy/Figure2B HHUA_GAPDH (the 3 central bands).tif]

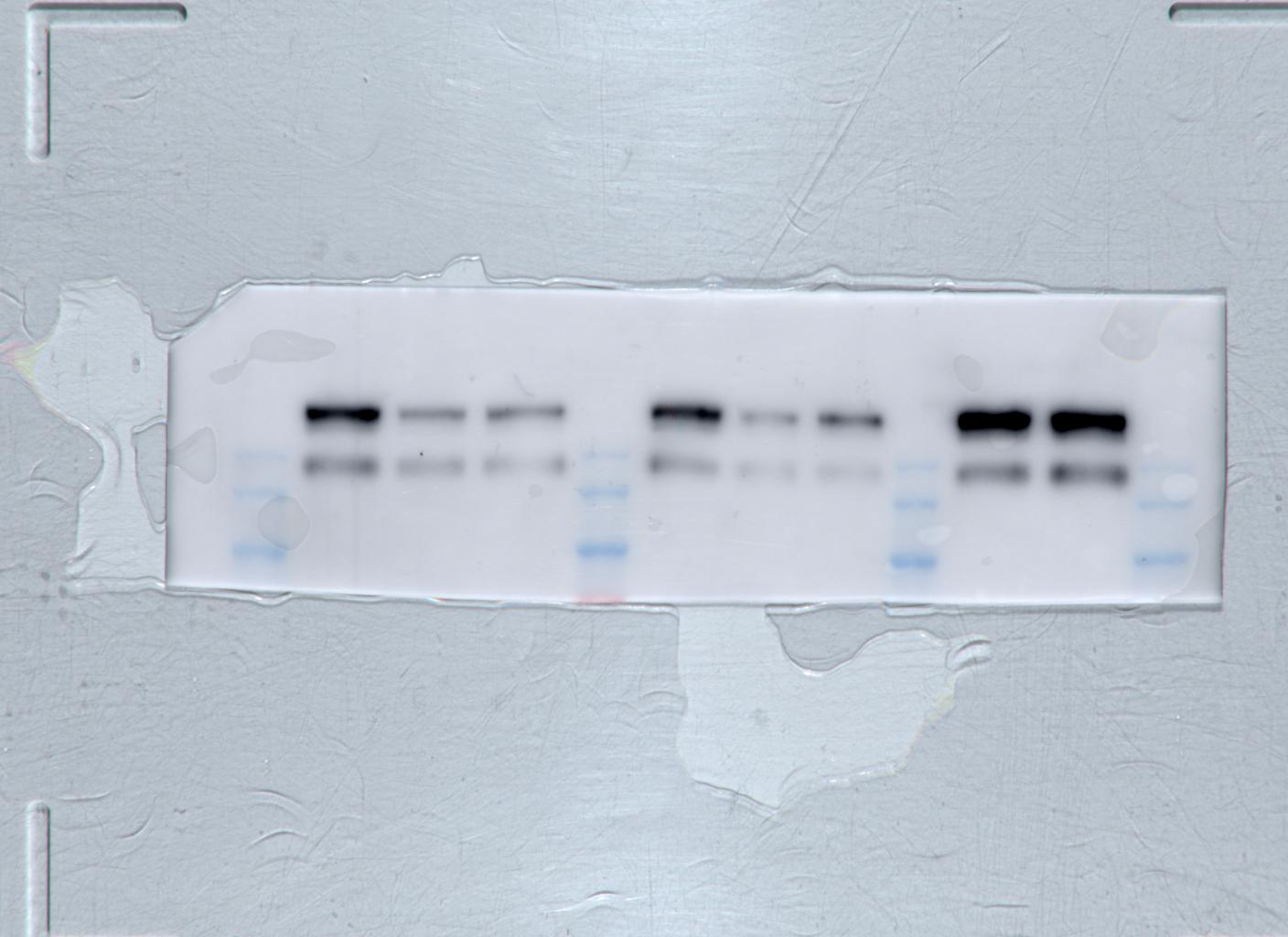

Supplement: Supplementary file 1 [file cancers-18-00198-s001.zip › Figure S1 and S2 Kurosu Original Images for Blots or Gels or Microscopy/Figure2B HHUA_L1CAM (the 3 central bands).tif]

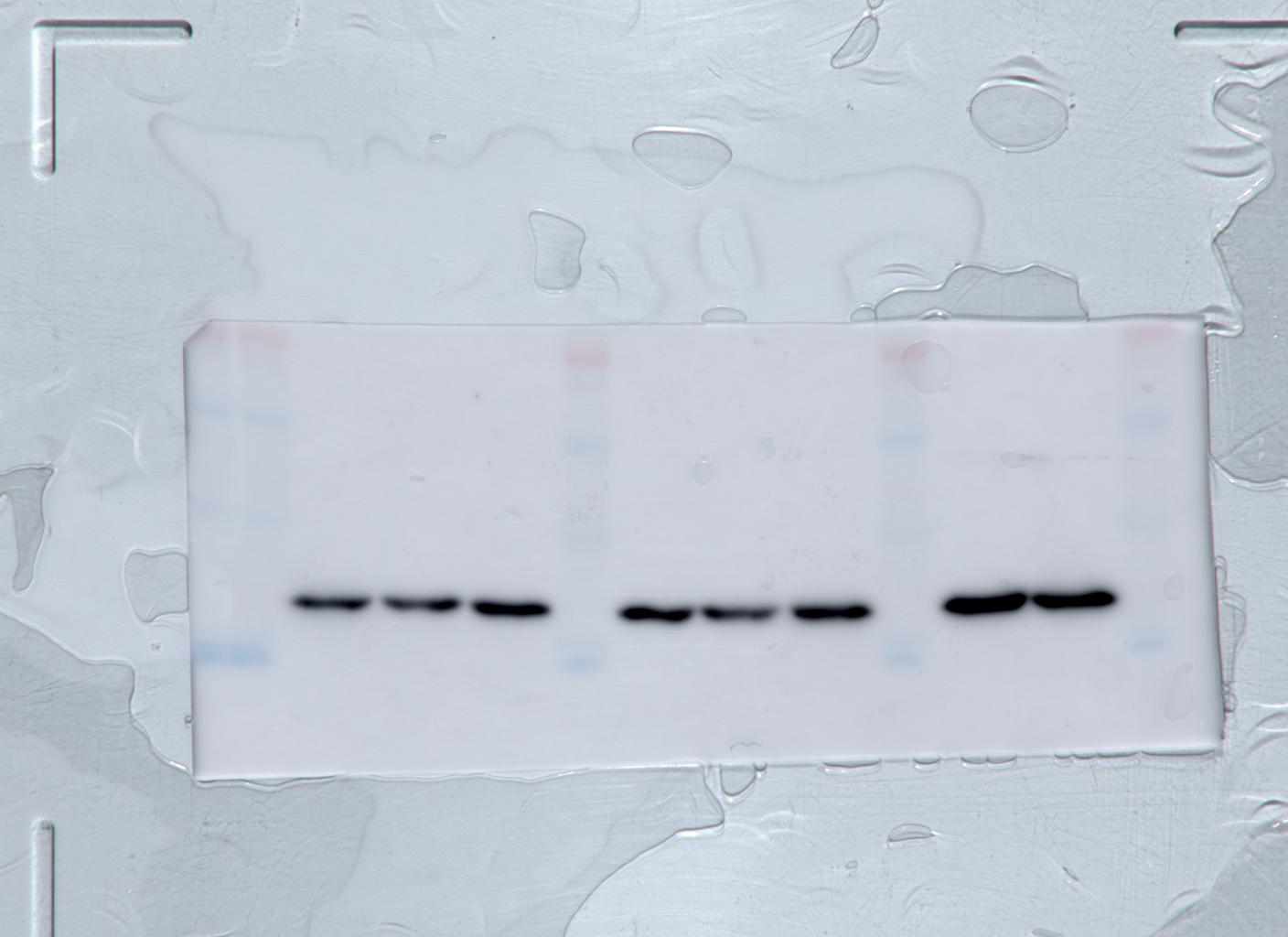

Supplement: Supplementary file 1 [file cancers-18-00198-s001.zip › Figure S1 and S2 Kurosu Original Images for Blots or Gels or Microscopy/Figure2B SPAC-1-L_GAPDH (the 3 central bands).tif]

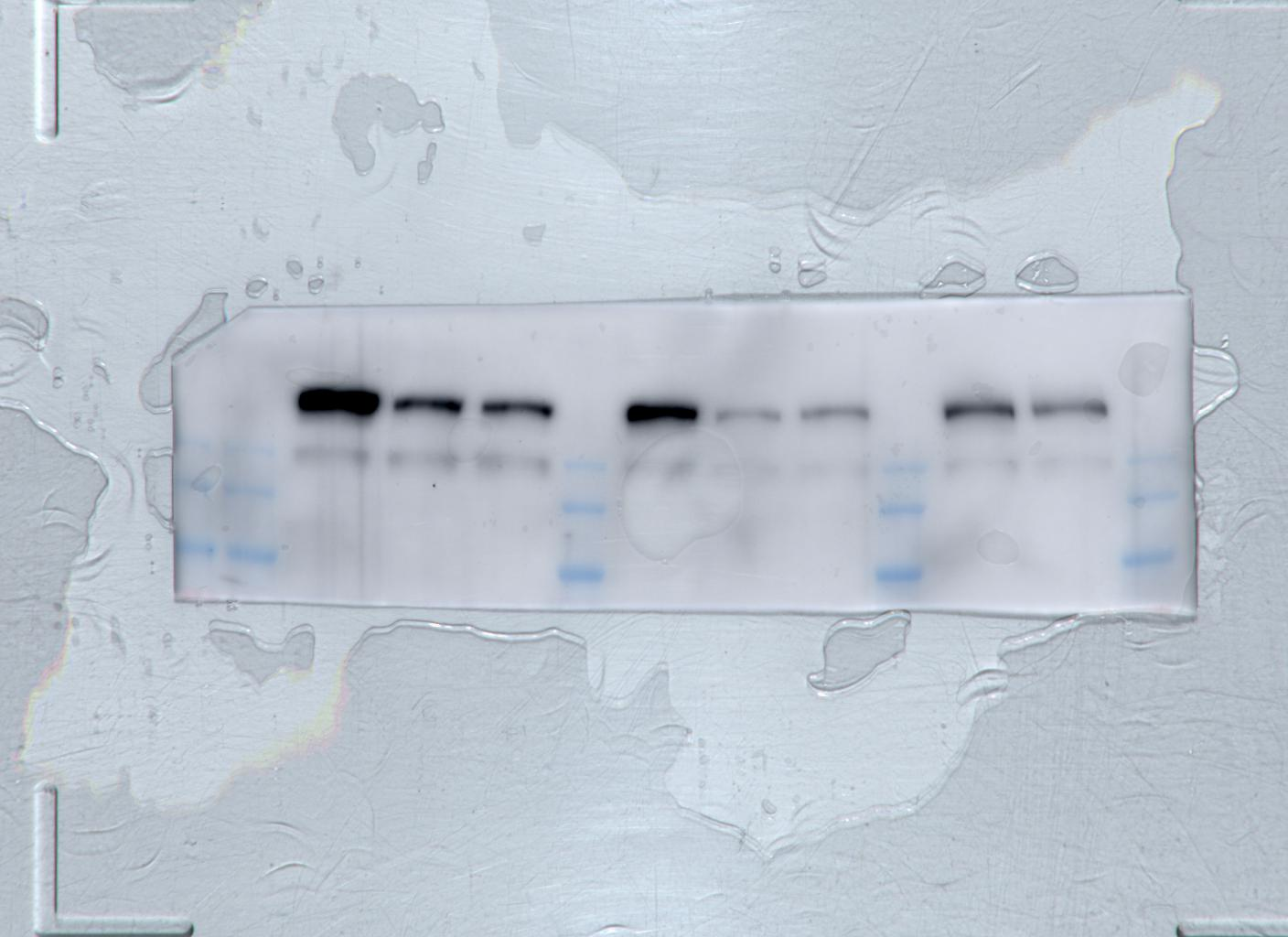

Supplement: Supplementary file 1 [file cancers-18-00198-s001.zip › Figure S1 and S2 Kurosu Original Images for Blots or Gels or Microscopy/Figure2B SPAC-1-L_L1CAM (the 3 central bands).tif]

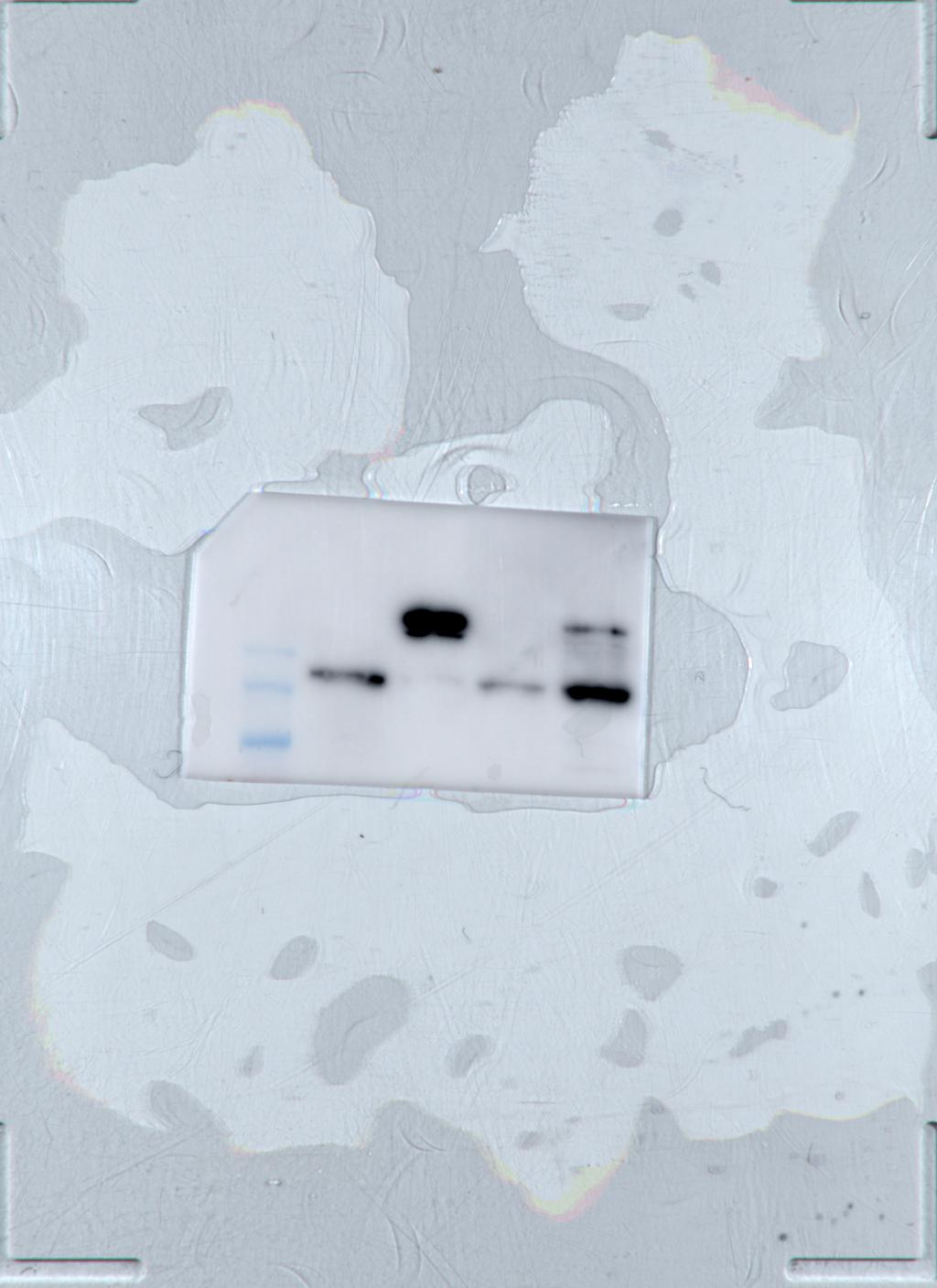

Supplement: Supplementary file 1 [file cancers-18-00198-s001.zip › Figure S1 and S2 Kurosu Original Images for Blots or Gels or Microscopy/Figure7B HHUA_Flag(L1CAM) (the 2 bands on the left).tif]

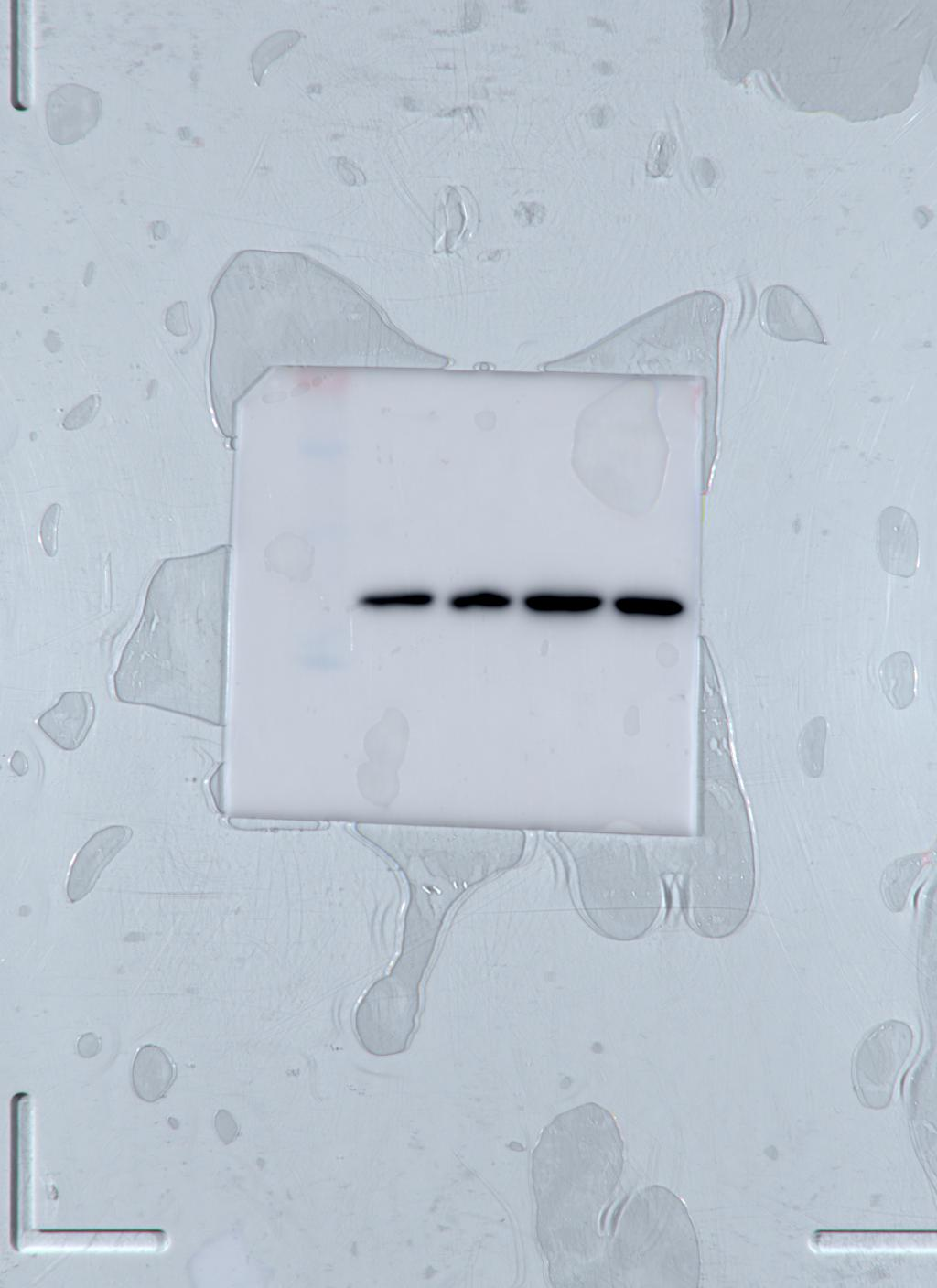

Supplement: Supplementary file 1 [file cancers-18-00198-s001.zip › Figure S1 and S2 Kurosu Original Images for Blots or Gels or Microscopy/Figure7B HHUA_GAPDH (the 2 bands on the left).tif]

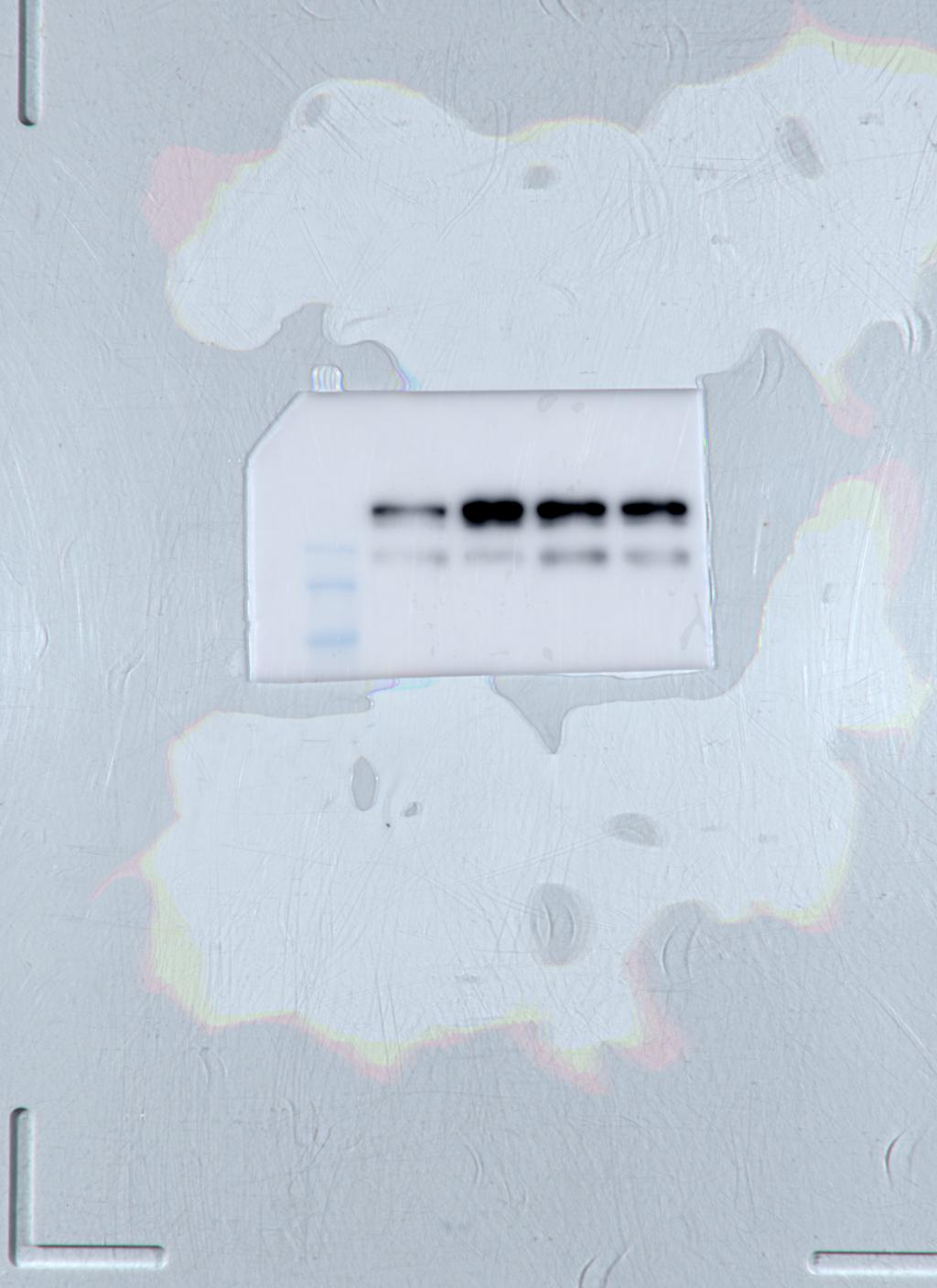

Supplement: Supplementary file 1 [file cancers-18-00198-s001.zip › Figure S1 and S2 Kurosu Original Images for Blots or Gels or Microscopy/Figure7B HHUA_L1CAM (the 2 bands on the left).tif]

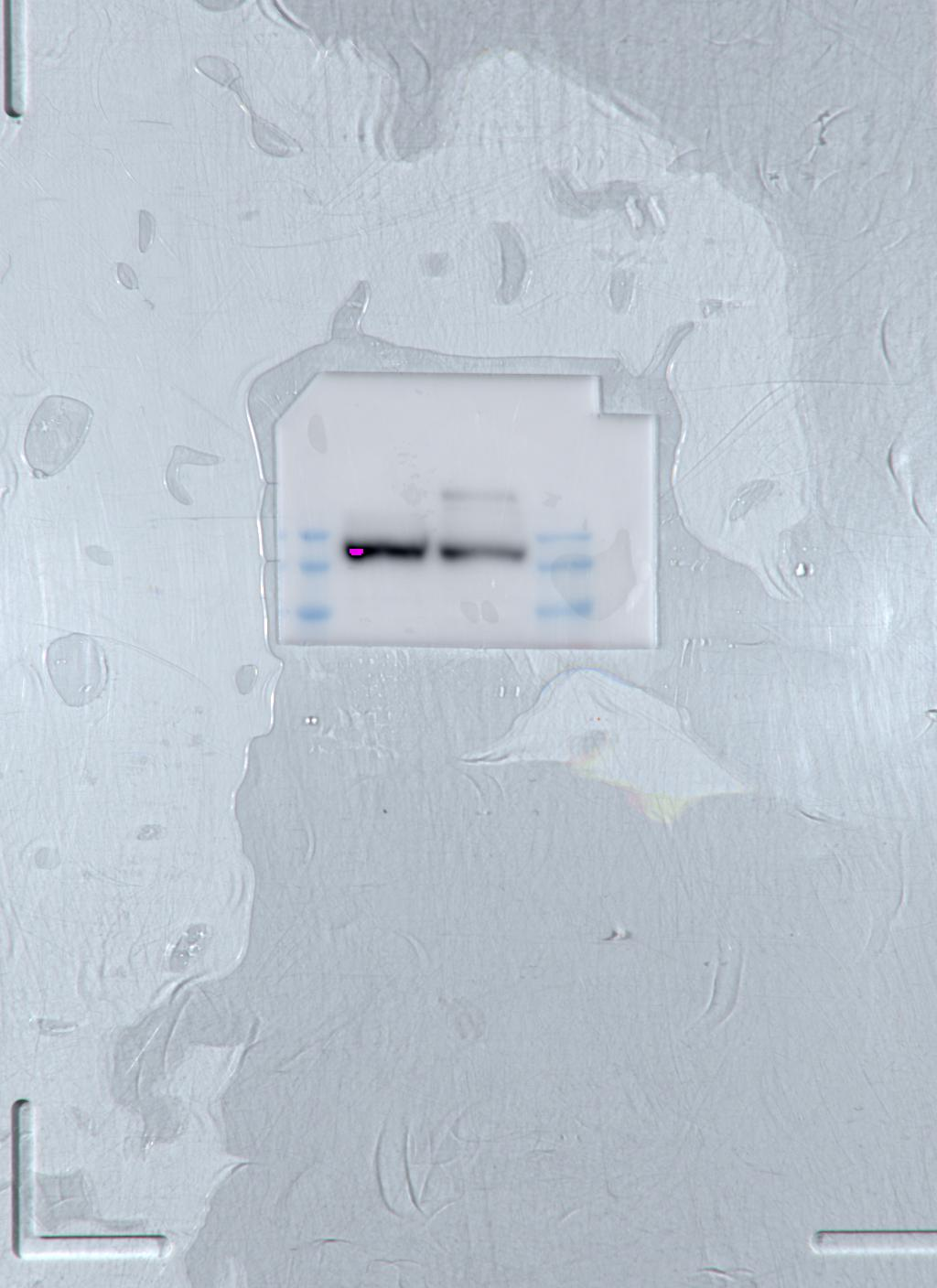

Supplement: Supplementary file 1 [file cancers-18-00198-s001.zip › Figure S1 and S2 Kurosu Original Images for Blots or Gels or Microscopy/Figure7B Ishikawa_Flag(L1CAM).tif]

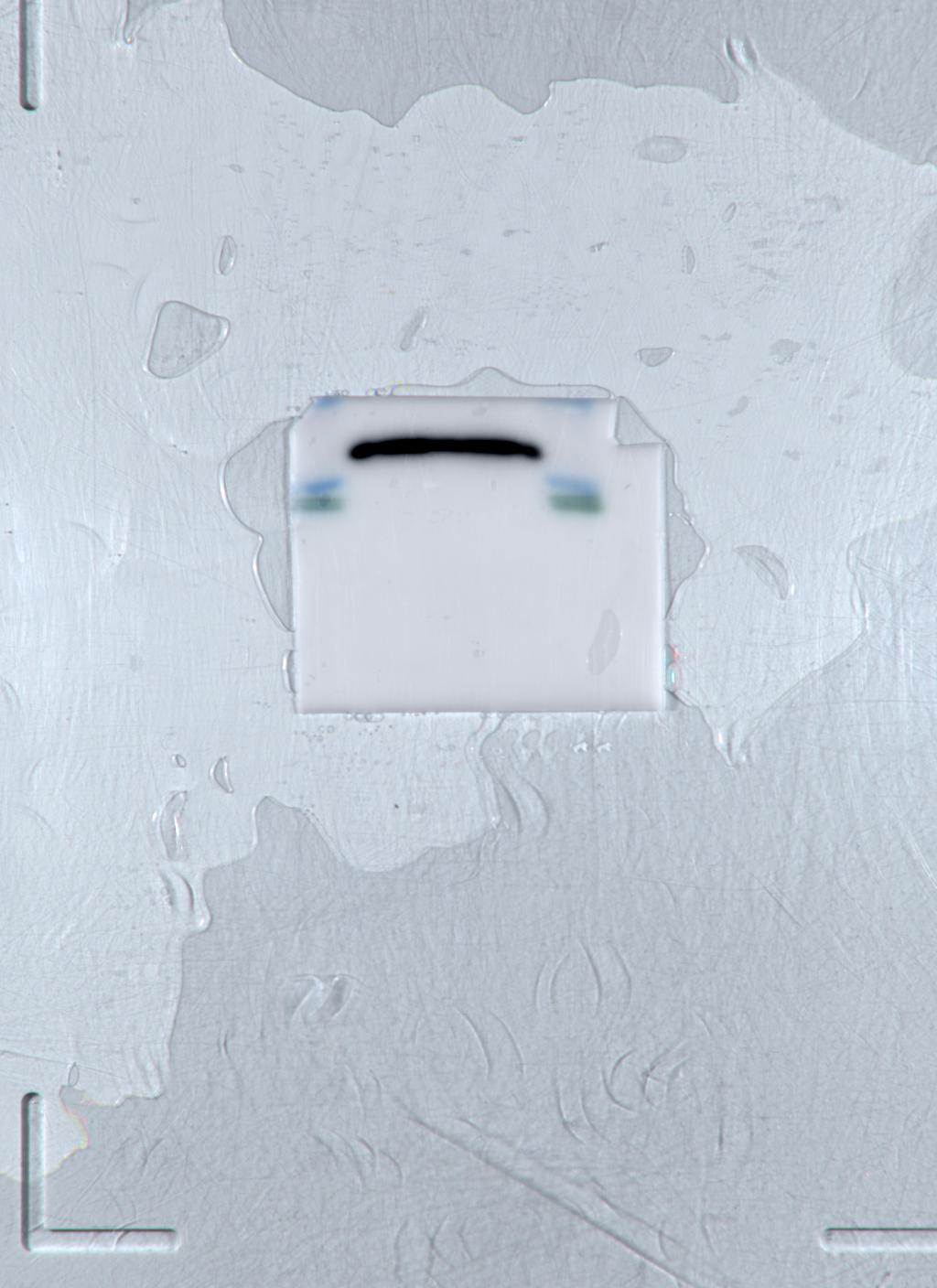

Supplement: Supplementary file 1 [file cancers-18-00198-s001.zip › Figure S1 and S2 Kurosu Original Images for Blots or Gels or Microscopy/Figure7B Ishikawa_GAPDH.tif]

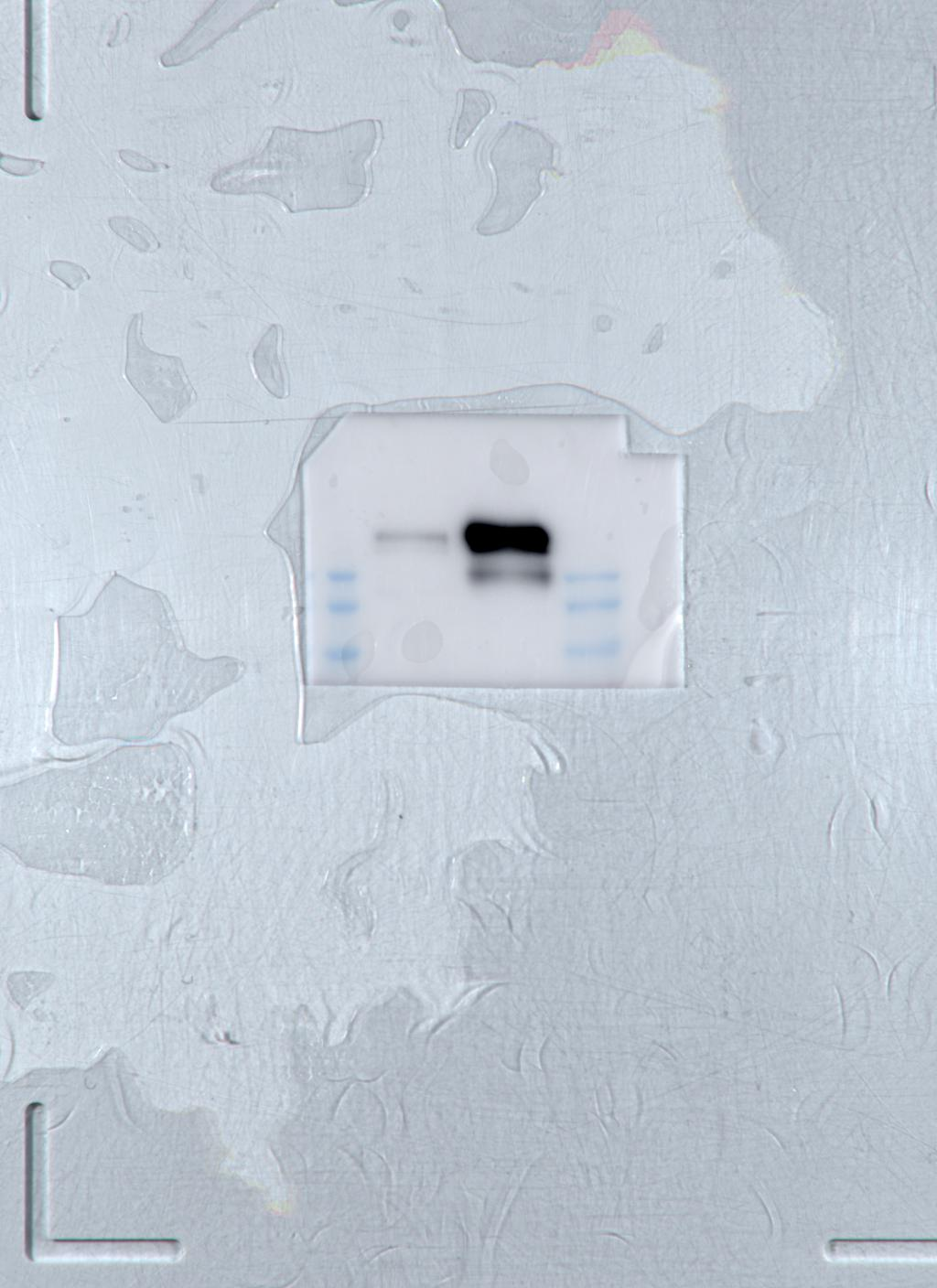

Supplement: Supplementary file 1 [file cancers-18-00198-s001.zip › Figure S1 and S2 Kurosu Original Images for Blots or Gels or Microscopy/Figure7B Ishikawa_L1CAM.tif]

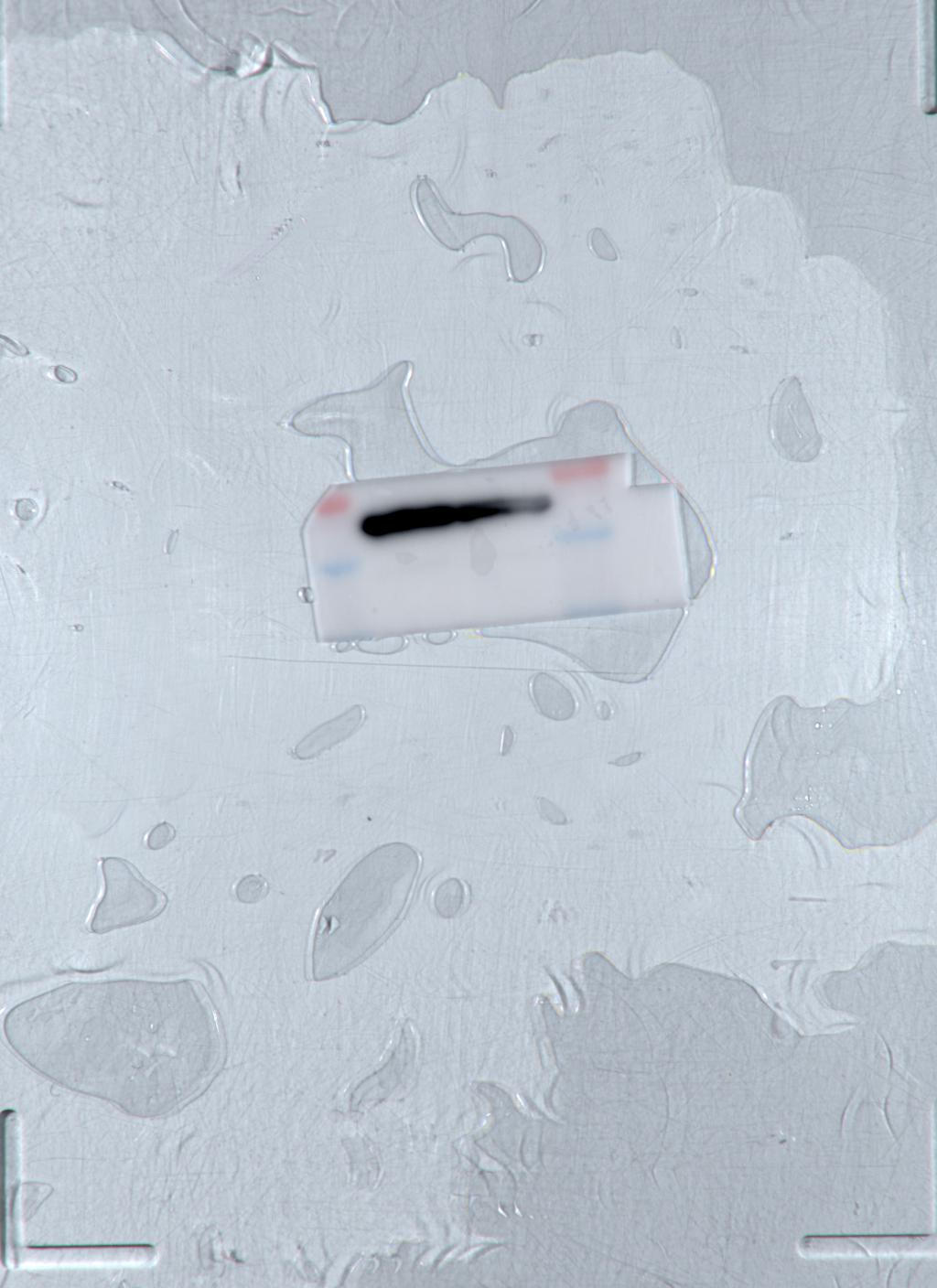

Supplement: Supplementary file 1 [file cancers-18-00198-s001.zip › Figure S1 and S2 Kurosu Original Images for Blots or Gels or Microscopy/Figure7B Ishikawa_Membrane between L1CAM and GAPDH (unused in figure7B).tif]

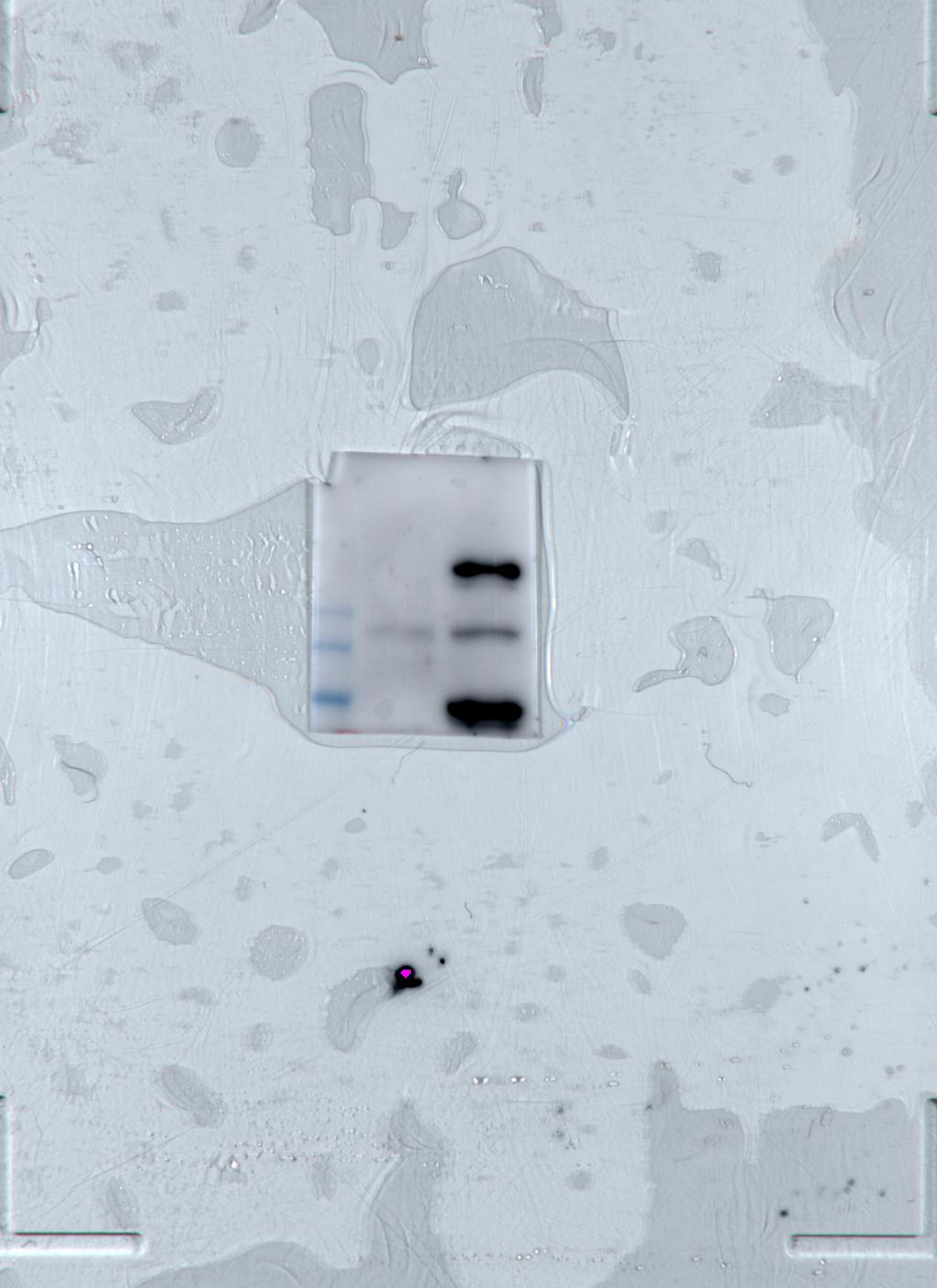

Supplement: Supplementary file 1 [file cancers-18-00198-s001.zip › Figure S1 and S2 Kurosu Original Images for Blots or Gels or Microscopy/Figure7B SPAC-1-L_Flag(L1CAM).tif]

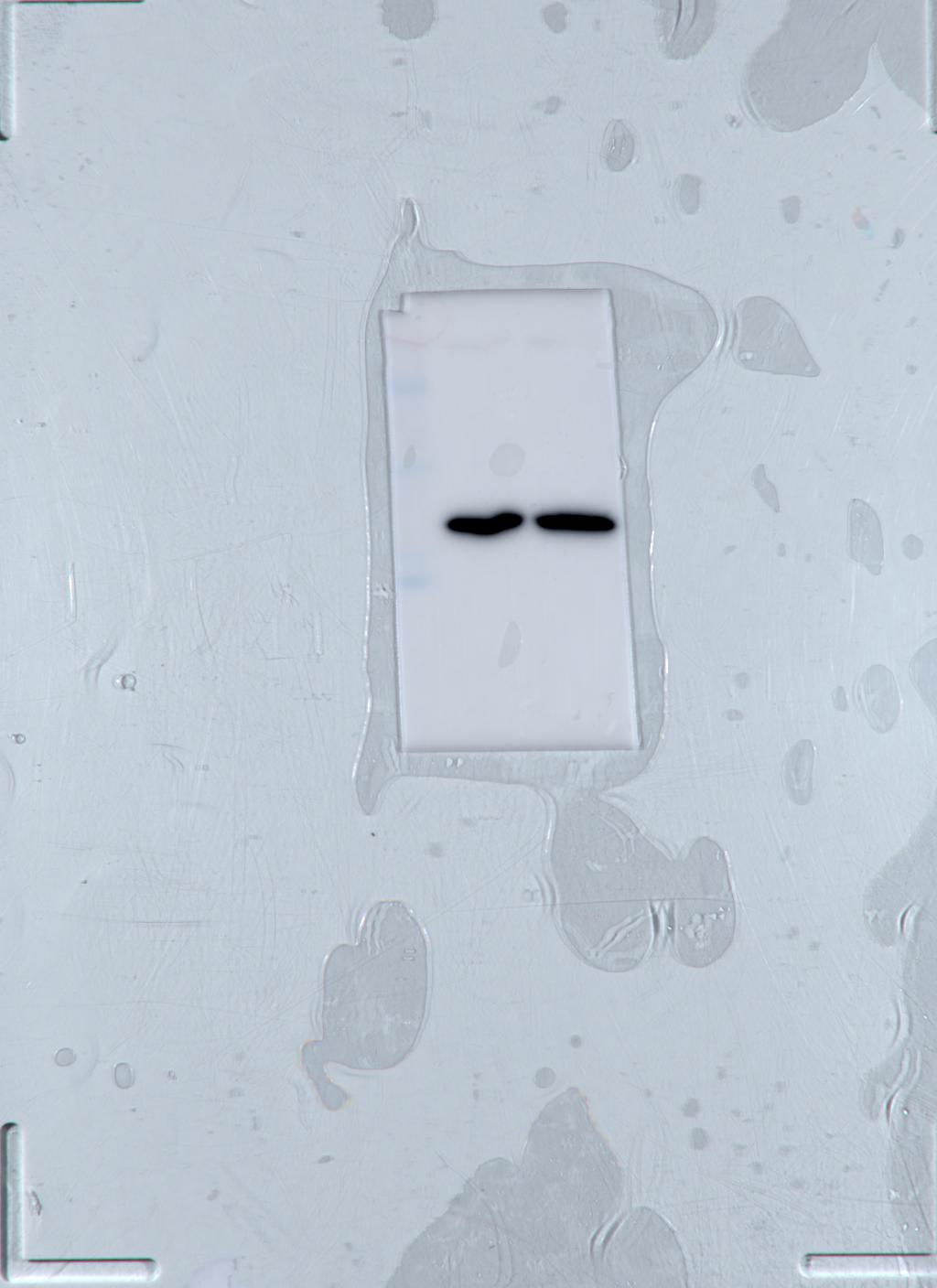

Supplement: Supplementary file 1 [file cancers-18-00198-s001.zip › Figure S1 and S2 Kurosu Original Images for Blots or Gels or Microscopy/Figure7B SPAC-1-L_GAPDH.tif]

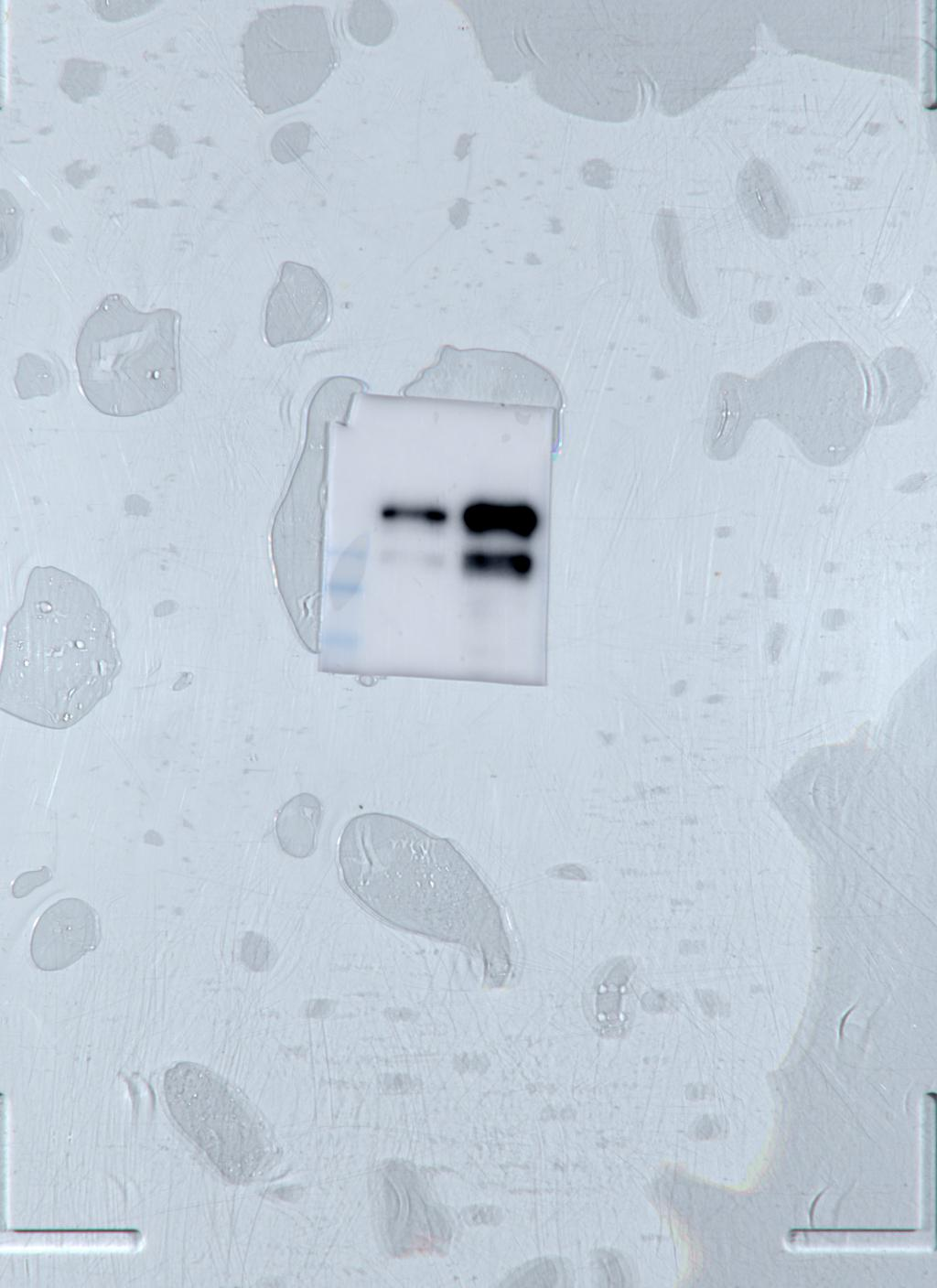

Supplement: Supplementary file 1 [file cancers-18-00198-s001.zip › Figure S1 and S2 Kurosu Original Images for Blots or Gels or Microscopy/Figure7B SPAC-1-L_L1CAM.tif]

Supplementary Figure S3

Cell cycle assay of HHUA cells with control or *L1CAM* knockdown (n=1)

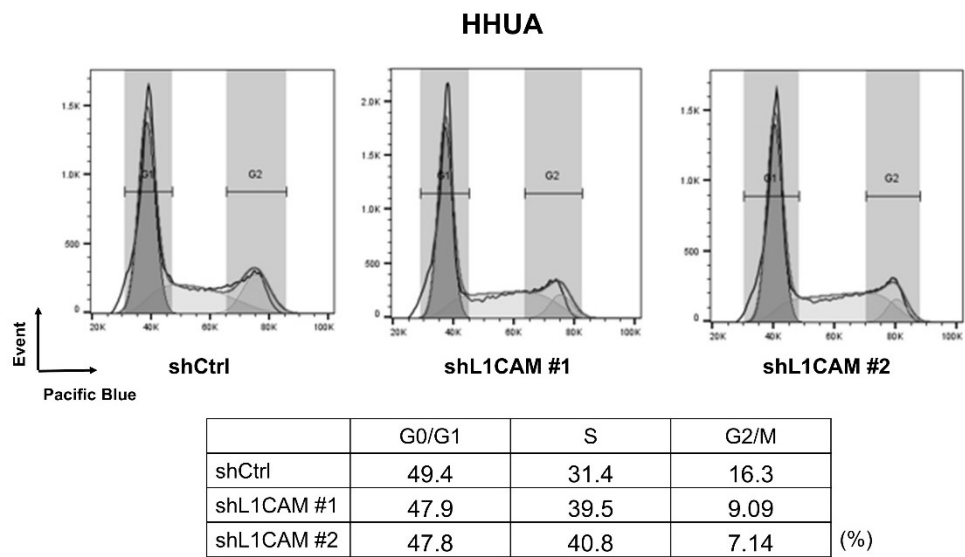

Supplement: Supplementary file 1 [file cancers-18-00198-s001.zip › Supplementary Figure S3 Cell cycle assay of HHUA with control or L1CAM knockdown.pdf]
